# Benchmarking long-read aligners and SV callers for structural variation detection in Oxford nanopore sequencing data

**DOI:** 10.1038/s41598-024-56604-2

**Published:** 2024-03-14

**Authors:** Asmaa A. Helal, Bishoy T. Saad, Mina T. Saad, Gamal S. Mosaad, Khaled M. Aboshanab

**Affiliations:** 1 Department of Bioinformatics, HITS Solutions Co., Cairo, 11765 Egypt; 2 Department of Microbiology and Immunology, Faculty of Pharmacy, Ain Shams University, Organization of African Unity St., Abassi, Cairo, 11566 Egypt

**Keywords:** Structural variations (SVs), Mutation detection, Human genome, Nanopore sequencing, Oxford nanopore technology, Biological techniques, Computational biology and bioinformatics

## Abstract

Structural variants (SVs) are one of the significant types of DNA mutations and are typically defined as larger-than-50-bp genomic alterations that include insertions, deletions, duplications, inversions, and translocations. These modifications can profoundly impact the phenotypic characteristics and contribute to disorders like cancer, response to treatment, and infections. Four long-read aligners and five SV callers have been evaluated using three Oxford Nanopore NGS human genome datasets in terms of precision, recall, and F1-score statistical metrics, depth of coverage, and speed of analysis. The best SV caller regarding recall, precision, and F1-score when matched with different aligners at different coverage levels tend to vary depending on the dataset and the specific SV types being analyzed. However, based on our findings, Sniffles and CuteSV tend to perform well across different aligners and coverage levels, followed by SVIM, PBSV, and SVDSS in the last place. The CuteSV caller has the highest average F1-score (82.51%) and recall (78.50%), and Sniffles has the highest average precision value (94.33%). Minimap2 as an aligner and Sniffles as an SV caller act as a strong base for the pipeline of SV calling because of their high speed and reasonable accomplishment. PBSV has a lower average F1-score, precision, and recall and may generate more false positives and overlook some actual SVs. Our results are valuable in the comprehensive evaluation of popular SV callers and aligners as they provide insight into the performance of several long-read aligners and SV callers and serve as a reference for researchers in selecting the most suitable tools for SV detection.

## Introduction

Structural variations (SVs) are one of the significant types of DNA mutation and are typically distinct as larger-than-50-bp genomic alterations that include insertions, deletions, duplications, inversions, and translocations^1,2^. Copy number variations (CNVs) are categorized as SVs, such as insertions, duplications, and deletions that include the addition or removal of genetic material and can therefore directly affect gene product. In humans, SVs account for most nucleotide distinctions between individuals^3,4^. The SVs have a significant influence on genome construction and are linked to several diseases, including inherited diseases^5^, neurological disorders^6^, cancer, evolution, and gene regulation^7^. Understanding the genomic architecture and associated genetic elements for various disorders requires a thorough understanding of SVs and their functional implications. Moreover, single nucleotide variants (SNVs) were believed to account for human beings’ mass of genomic changes^8,9^. Despite their importance, SVs have received far less attention than SNVs, particularly in low-complexity areas recognized as SV hotspots^4,10^. Indeed, it has been demonstrated that repeats cause uncertainties in short reads, introducing faults in calling chromosomal or DNA variations^11,12^.

In recent years, DNA sequencing has emerged as one of the primary methods for identifying SV^1,10,12^. Still, Array Comparative Genomic Hybridization (aCGH) was also used to detect structural variants. Microarray technology is used in aCGH, where probes are created to cover the entire genome. Unbalanced SVs can be detected by measuring two samples' relative copy number differences. Since 2005, next-generation sequencing (NGS) has been commonly utilized in genomic exploration^10,12^. Recently, third-generation sequencing technologies have enabled the generation of significantly longer reads, propelling advances in variant calling and genome assembly^13–15^. In addition, the Pacific Biosciences Long Reads and the ONT have recently appeared and demonstrated their value in detecting intractable DNA sequences^15–17^.

The long-range spanning information allows for more comprehensive detection of SVs at a higher resolution^10^. However, short-read-based SV calling approaches have been established to distinguish SVs^18,19^. Most of them employ discordant read-pairs^20^, local assembly^21^, split read alignments^22^, read-depths^18,23^, or pairing of these methods^24,25^. These methods were applied in large-scale genomics studies^26^. On the other hand, these tools being designed for short reads limited their ability to apply efficient SV detection, leading to many false positive results^27,28^. There are two approaches for structural variant calling: De novo assembly and Read alignment-based^2,29^. Assembly-based approaches are much more computationally expensive than alignment-based approaches and have several problems in reconstructing large genome haplotypes^19,27^. Several long-read alignment-based SV callers for reads generated from PacBio and ONT, including SMRT-SV https://github.com/EichlerLab/smrtsv2 (accessed on 3 September 2023), PBSV, SVIM, Sniffles, and CuteSV, as well as newly developed SV calling tools such as SVDSS and SVcnn, have been proposed. To detect SVs, they employ various analysis methods^29,30^. Furthermore, cutting-edge long-read aligners like long-read aligners (LRA)^31^, NGMLR, Minimap2, and Pbmm2 were typically used for the read alignment. Since the ONT sequencers were newly released, SV detection have not been deeply established, leaving room for improvement; our findings provide an estimate of variation content in a human genome to date, serve as a valuable resource of SVs for other studies, and emphasize the importance of employing multiple strategies for SV discovery.

Therefore, in this study, five common SV callers, including CuteSV, Sniffles, PBSV, SVDSS, and SVIM, and four common long read aligners (minimap2, LRA, NGMLR, and pbmm2) have been evaluated using a Human Reference dataset HG002 (NA24385), HG001 (NA12878), and a simulated data SI00001 to enable the accurate evaluation of the output for the SV callers. The evaluation of five SV callers in terms of precision, recall, and F1-score statistical metrics and the four aligners according to depth of coverage, speed of analysis, and efficiency of detection and data assessment.

## Materials and methods

### The selection of the validation datasets for SV calling

For benchmarking the existing structural variant calling methods, it is preferable to use multiple datasets, accordingly, three datasets have been used in this evaluation workflow. The first dataset was an ONT real dataset, in FASTQ format, sequenced on PromethION and released by the GIAB consortium for the NA24385 Ashkenazim individual in (https://ftp-trace.ncbi.nlm.nih.gov/giab/ftp/data/AshkenazimTrio/HG002_NA24385_son/Ultralong_OxfordNanopore/guppy-V3.4.5/ (accessed on 3 September 2023), the Genome in a Bottle (GIAB) Consortium created benchmark SV calls and benchmark regions (https://ftp.ncbi.nih.gov/giab/ftp/data/AshkenazimTrio/analysis/NIST_SVs_Integration_v0.6/HG002_SVs_Tier1_v0.6.vcf.gz) (accessed on 3 September 2023). This “Truth set” is considered a resource of highly curated and high-quality variants and was published to the research community. SV calling methods have been released based on the hg19 coordinates. The second dataset was an ONT real dataset, in FASTQ format, sequenced on MinION using a 1D ligation kit and obtained from the Nanopore repository (https://github.com/nanopore-wgs-consortium/NA12878/blob/master/nanopore-human-genome/rel34.md (accessed on 3 September 2023). The SV truth set, for this dataset, was generated by the Genome in a Bottle Consortium using the Pacific Biosciences (PacBio) platform and was used, in this manuscript, as the corresponding SV truth set for the NA12878 dataset. The analysis only included SV calls with a "PASS" flag in the "FILTER" field (https://ftp-trace.ncbi.nlm.nih.gov/giab/ftp/data/NA12878/NA12878_PacBio_MtSinai/NA12878.sorted.vcf.gz).

The last dataset was a synthetic ONT data, referred to as SI00001, generated using the SV simulator VarIant SimulatOR (VISOR) (https://github.com/davidebolo1993/VISOR) (accessed on 3 September 2023), as per the simulation instructions to generate the ONT long reads, and was simulated to 50X coverage^32^. The VISOR was also used to generate an SV truth set of variants that harbor deletions insertions, duplications, and translocations. The calls reported were the ones with PASS in the FILTER field and of SV length >=50bp.

### Read mapping and structural variant calling for datasets

The three datasets reads were aligned to the public human genome build GRCh37/UCSC hg19 using four long-read aligners “Minimap2”^33^ (v2.26), “NGMLR”^34^ (v.0.2.7), “LRA”^31^ (v1.3.7.2), and “pbmm2” https://github.com/PacificBiosciences/pbmm2 (v1.7.0) (Table 1). The reason for the alignment of the reads to the previous version of the human reference genome is that the “Benchmark set” for NA12878 and “Truth set” for NA24385, that will be later used as a benchmark reference for this evaluation process, was on the hg19. Also, the SV benchmark set simulated with VISOR was performed using the hg19 build to unify the reference genome build. After the completion of the alignment, a Sequence Alignment Map (SAM) file was generated, which was then converted to Binary Alignment Map (BAM) format using Samtools^35^. The resulting BAM file was sorted and indexed with Samtools to prepare the file for variant calling. Mosdepth was used to calculate the coverage after sorting and indexing the generated alignments^36^.

**Table 1:** Summary of the tools used for SV calling, annotation, and benchmarking.

Tool	Type	Version	GitHub repository
Minimap2	Aligner	2.26	https://github.com/lh3/minimap2
LRA	Aligner	1.3.7.2	https://github.com/ChaissonLab/LRA
NGMLR	Aligner	0.2.7	https://github.com/philres/ngmlr
Pbmm2	Aligner	1.7.0	https://github.com/PacificBiosciences/pbmm2
Sniffles2	SV caller	2.0.7	https://github.com/fritzsedlazeck/Sniffles
CuteSV	SV caller	1.0.10	https://github.com/tjiangHIT/CuteSV
SVIM	SV caller	2.0.0	https://github.com/eldariont/svim
Pbsv	SV caller	2.3.0	https://github.com/PacificBiosciences/pbsv
SVDSS	SV caller	1.0.5	https://github.com/Parsoa/SVDSS
NpINV	Sv caller	1.28	https://github.com/haojingshao/npInv
VISOR	SV simulator for short and long reads	1.1.2	https://github.com/davidebolo1993/VISOR
Samtools	File manipulation toolkit	1.17	https://github.com/samtools/
Mosdepth	BAM depth calculator	0.3.3	https://github.com/brentp/mosdepth
Bcftools	VCF manipulation toolkit	1.17	https://github.com/samtools/bcftools
Truvari	Benchmarking toolkit	4.0.0	https://github.com/ACEnglish/truvari

In addition, the impact of different coverage depths on the SV caller's ability to identify both genomic cutoff points and genotypes has been investigated. Samtools was used to generate different coverages 30X, 20X, and 10X for better evaluation of the SV callers to achieve down-sampling for the BAM file. To perform the evaluation, four SV callers were tested in parallel on each dataset to provide an insight into the SV callers’ performance (Table 1): (1) CuteSV (v1.0.10), (2) SVIM (v.2.0.0), (3), PBSV (v2.3.0) and (4) Sniffles2 (v2.0.7). Sniffles2 is a tool that is fully integrated into a Nextflow-based workflow “Epi2me-labs/ wf-human-variation”, provided by Nanopore, that uses Minimap2 as an aligner (https://github.com/epi2me-labs/wf-human-variation)(accessed on 3 September 2023). Two newly developed SV callers, SVDSS^37^ and SVcnn^38^ were included to explore their potential as candidates for the state-of-the-art SV callers (Table 1).

### Enhancing the SV calling accuracy

For enhancing the SV calling accuracy, a tandem repeat Browser Extensible Data (BED) file corresponding to the hg19 reference (https://raw.githubusercontent.com/PacificBiosciences/pbsv/master/annotations/human_hs37d5.trf.bed) (accessed on 3 September 2023), was downloaded and used during the variant calling process. Even though Sniffles, SVIM, CuteSV, and PBSV can find all kinds of SV, NpInv was designed to detect inversions accurately. Detection for Inversions (INV) was not in the scope of the current evaluation, but still, it was performed to lay the ground for the future assessment of SV callers on the level of accurate inversion detection.

### Length-based binning of reported SVs

The generated VCFs for each variant caller were divided into 7 groups based on their respective lengths: (A) <50 bp, (B) 50–250 bp, (C) 251–500 bp, (D) 501–750 bp, (E) 751–1000 bp, (F) 1001–5000 bp, and (G) > 5000 bp, to get insights about the performance of the variant callers across different SV sizes.

### Filtering for the SV callset

Numerous filtering was accomplished to generate comparable datasets. The SV calls from independent consensus sequences or contigs, and the mitochondrial genome was filtered out leaving only insertions, duplications, and deletions for each call set. For comparison, insertion and duplication calls were combined into one category ("insertions"). The SVs were then filtered for length >=50 bp, and only SV calls with a “PASS” flag in the “FILTER” column were filtered in for the next step of the analysis. The performance of SV detection tools was challenging to evaluate because there is no standard technique for precisely identifying SVs in the homo sapiens genome. The “Truth set”/ “Benchmark set” Variant Call Formats (VCFs) corresponding to the three datasets from GIAB and VISOR were used to address this limitation. The output VCFs of the five SV callers were then compared to this “Truth set”/”Benchmark set” VCF in terms of precision, recall, and F1-score statistical metrics using the toolkit “Truvari” (Table 1) to target the impact of sequencing settings on of the SVs generated from each tool and how close it is to the “Truth callset” where the candidate SVs missing from the truth were reflected false positives, and vice versa for false negatives.

## Results

### Alignment of ONT datasets using long-read aligners and corresponding truth SV call sets

**Figure 1:**
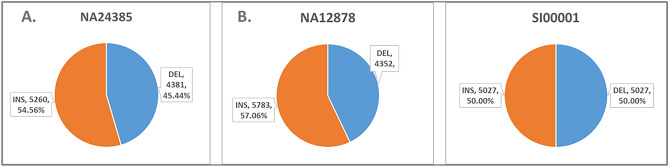
The number distribution of Deletions (DEL) and Insertions (INS) for the NA24385 Truth set, NA12878, and SI00001 benchmark sets.

For the NA24385 dataset, the GIAB consortium's ultra-long ONT FASTQ was used for the evaluation process after their retrieval from the NCBI repository. The initial total coverage was found to be 45X and was down-sampled to depths of coverage of 30X, 20X, and 10X. The truth callset has a great amount of deletions or insertions produced from various sequence lengths and visual charting for the same individual on GRCh37 genome. The NA24385 truth SV callset has 9641 SVs (with FILTER "PASS”), with 5260 insertions and 4381 deletions (Fig. 1). The FASTQ file generated by the nanopore whole-genome sequencing consortium was used for the alignment process. The reported and the calculated depth of coverage was found to be ~ 30X. Then, it was down-sampled to 20X and 10X coverage only. The SV call set is used as a corresponding created by the Genome in a Bottle Collaboration utilizing the Pacific Biosciences (PacBio) platform to generate the equivalent SV true set. There are 10,135 SVs in the NA12878 Benchmark callset (with FILTER "PASS"), with 5783 insertions and 4352 deletions (Fig. 1). The generated synthetic ONT dataset SI00001 was simulated using the SV simulator VISOR at a depth of coverage of 50X. The SV “Benchmark set” used for this dataset included 10,676 randomly generated SVs, which were then divided into 5,027 deletions and 5,027 insertions, and 300 inversions, among other types of structural variants such as duplications and translocations (Fig. 1). The SI00001 aligned bam file was down-sampled into 30X, 20X, and 10X depth of coverage. Generally, each aligner performed equally across the three datasets. In terms of time consumed, Minimap2 was the fastest of the four aligners (8 h), followed closely by LRA (14 h) and Pbmm2 (15 h), whereas NGMLR was the slowest (59 h). The alignment was done on a machine with 128 GB of RAM and 64 threads. The performance of the four aligners was represented in terms of the time taken by the tool to finish the alignment, the CPU time in hours, the wall clock, and the memory usage in gigabytes (Table 2). The metrics for the generated BAM following the four aligners were deposited into the GitHub repository (https://github.com/AnkhBioinformatics/SVcallers_Comparisons).

**Table 2:** Performance and resource consumption of Aligners regarding running time and memory usage.

GM24385		CPU time (hours)	CPU usage (%)	Wall clock (h:mm: ss or m: ss)	Memory usage (Gbytes)
Alignment	Minimap2	49.7	1137	8:25:15	21.7
	LRA	111.3	789	14:11:46	28.8
	NGMLR	535.2	909	59:46:42	14.4
	Pbmm2	57.8	1051	15:41:00	21.9

### Evaluation of the different SV callers’ performance in terms of precision, recall, and F-score values for SV calling of the NA24385, NA12878 and simulated SI00001 human genome datasets

The chosen four commonly used long-read sequencing SV callers (CuteSV, SVIM, Sniffles, and PBSV) were usually tested against publicly available ultra-long nanopore reads of truth set NA24385 at varying coverages. In addition to that dataset, the NA12878 and SI00001 datasets were added to enhance the power of the evaluation for the SV callers’ performance. It is worth mentioning that the SVcnn caller was previously considered for this evaluation but later rejected as it was extensively time-consuming (80 h and 27.8 GB memory) and crashed repeatedly, so it was not included in the evaluation.

All SV callers were pre-tuned to detect SV of 50 bp and above to unify the parameters for all the callers. As for the filtering of the output VCF generated from each tool, only SVs with “PASS” in the FILTER field and lay in the regions of the 1–22, X and Y chromosomes was regarded as a candidate for evaluating the results of the tools. Calls not matching any true variants are regarded as false positives. In contrast, false negatives were considered callset variants that are not present in the truth set. For combinations of the mentioned aligners and SV callers, we assessed the detected SVs' precision, recall, and F1-score. Each tool's SV calls were marked "true" or "false" according to whether they match with the matching Truth/Benchmark callset. The output of the comparison process was a report with the information generated, including the precision, recall, and F1-score of the obtained high-quality SV callsets. This helped us evaluate the quality of the SV calls for each tool as well as the performance of each tool in terms of CPU time in hours, wall clock, and memory usage in gigabytes, which is presented in Table 3.

**Table 3:** SV callers’ resource consumption and performance in terms of CPU time, wall clock, and memory usage.

Aligner	SV caller	CPU time (hours)	CPU usage (%)	Wall clock (Minutes )	Memory usage (Gbytes)
Minimap2	CuteSV	5.1	354	90.6	3.4
	Sniffles	4.5	568	110.6	4.0
	SVIM	1.3	47	165.2	0.9
	PBSV-discover	1.9	99	114.7	1.1
	PBSV-call	10.6	305	223.6	56.1
	SVDSS-smooth	2.2	164	86.4	32.0
	SVDSS-search and assemble	7.7	808	57.7	15.2
	SVDSS-call	3.2	182	121.7	17.0
LRA	CuteSV	4.1	433	58.8	3.5
	Sniffles	4.1	569	98.9	6.0
	SVIM	4.9	64	530.7	5.5
	PBSV-discover	3.7	82	271.2	3.3
	PBSV-call	11.8	455	170.8	51.5
	SVDSS-smooth	2.4	486	30.9	34.2
	SVDSS-search and assemble	14.6	840	104.6	15.4
	SVDSS-call	4.1	601	2776.1	38.3
NGMLR	CuteSV	5.7	247	208.5	3.4
	Sniffles	5.5	568	231.1	3.6
	SVIM	3.0	53	354.6	2.7
	PBSV-discover	2.8	75	223.1	11.0
	PBSV-call	9.5	355	183.8	54.0
	SVDSS-smooth	2.3	171	85.6	34.0
	SVDSS-search and assemble	15.2	833	110.1	16.3
	SVDSS-call	3.6	206	118.3	17.0
Pbmm2	CuteSV	2.2	643	21.3	3.4
	Sniffles	2.9	341	22.3	7.0
	SVIM	4.6	50	504.8	4.5
	PBSV-discover	3.2	79	244.9	12.5
	PBSV-call	3.8	399	62.7	38.0
	SVDSS-smooth	2.3	112	129.8	34.0
	SVDSS-search and assemble	5.2	762	41.5	15.2
	SVDSS-call	2.5	1007	17.3	15.0

*BAM* Binary Alignment Map, *LRA* Long Read Aligner, *NGMLR* CoNvex Gap-cost alignMents for Long Reads, *SV* Structural Variant, *SVIM* Structural Variant Identification Method, *PBSV* Pacific Biosciences Structural Variant, Sniffles, CuteSV, Structural Variant SVIM and PBSV (SV detection tools).

**Figure 2:**
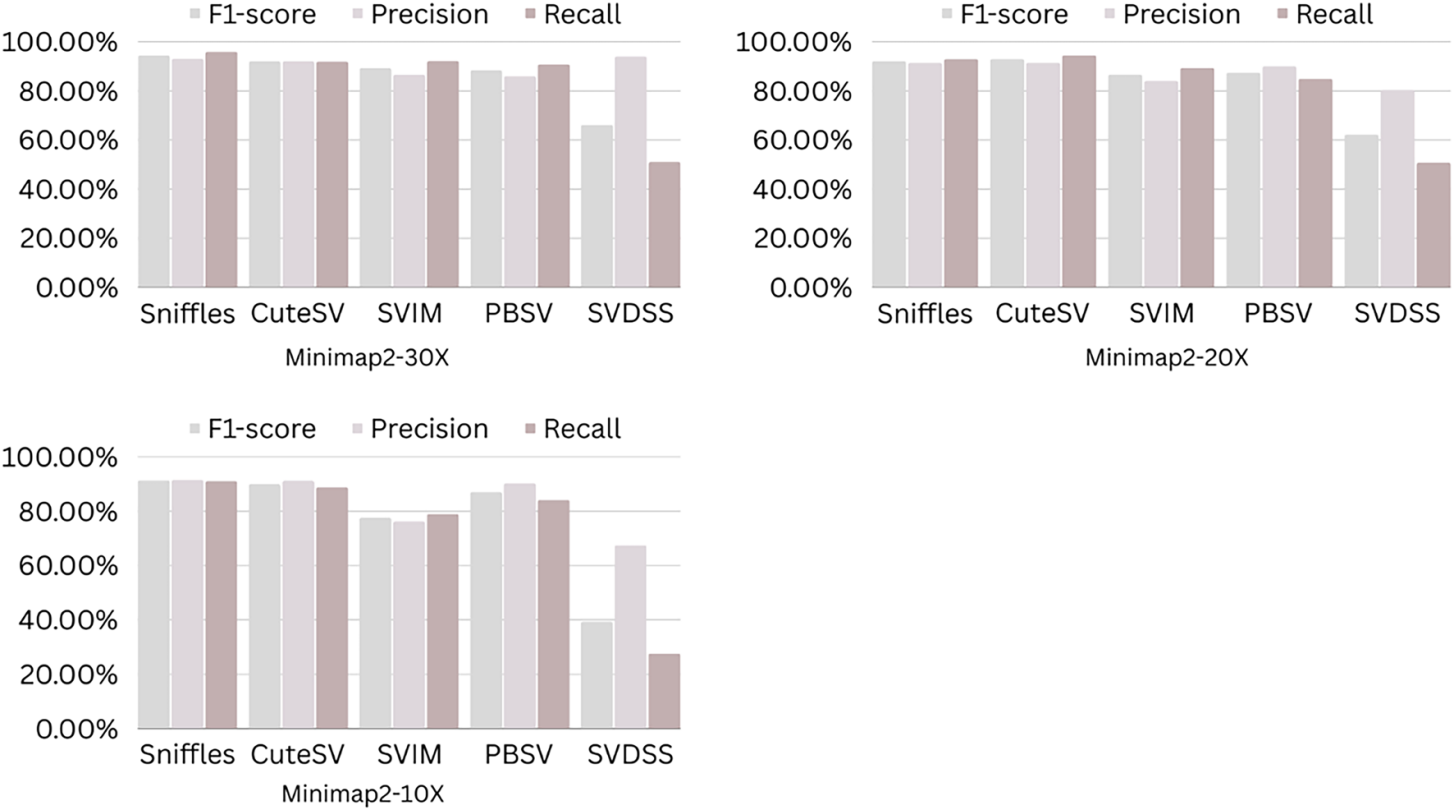
The F1-score, Precision and Recall (Y-axis) of different variant callers (Sniffles, CuteSV, SVIM, PBSV and SVDSS) with Minimap2 aligner for the NA 12,878 dataset at different sequencing coverages.

**Figure 3:**
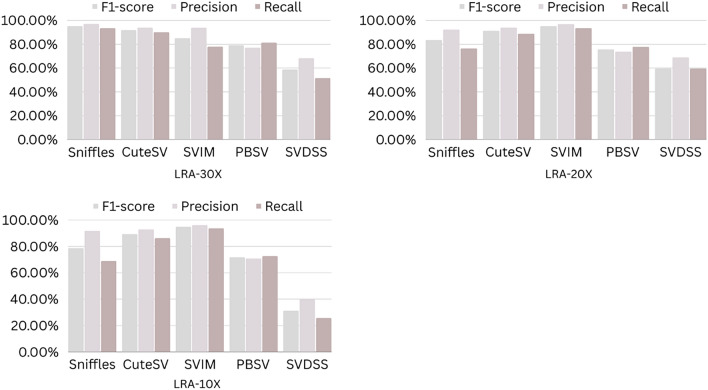
The F1-score, Precision and Recall (Y-axis) of different variant callers (Sniffles, CuteSV, SVIM, PBSV and SVDSS) with LRA aligner for the NA 12,878 dataset at different sequencing coverages.

**Figure 4:**
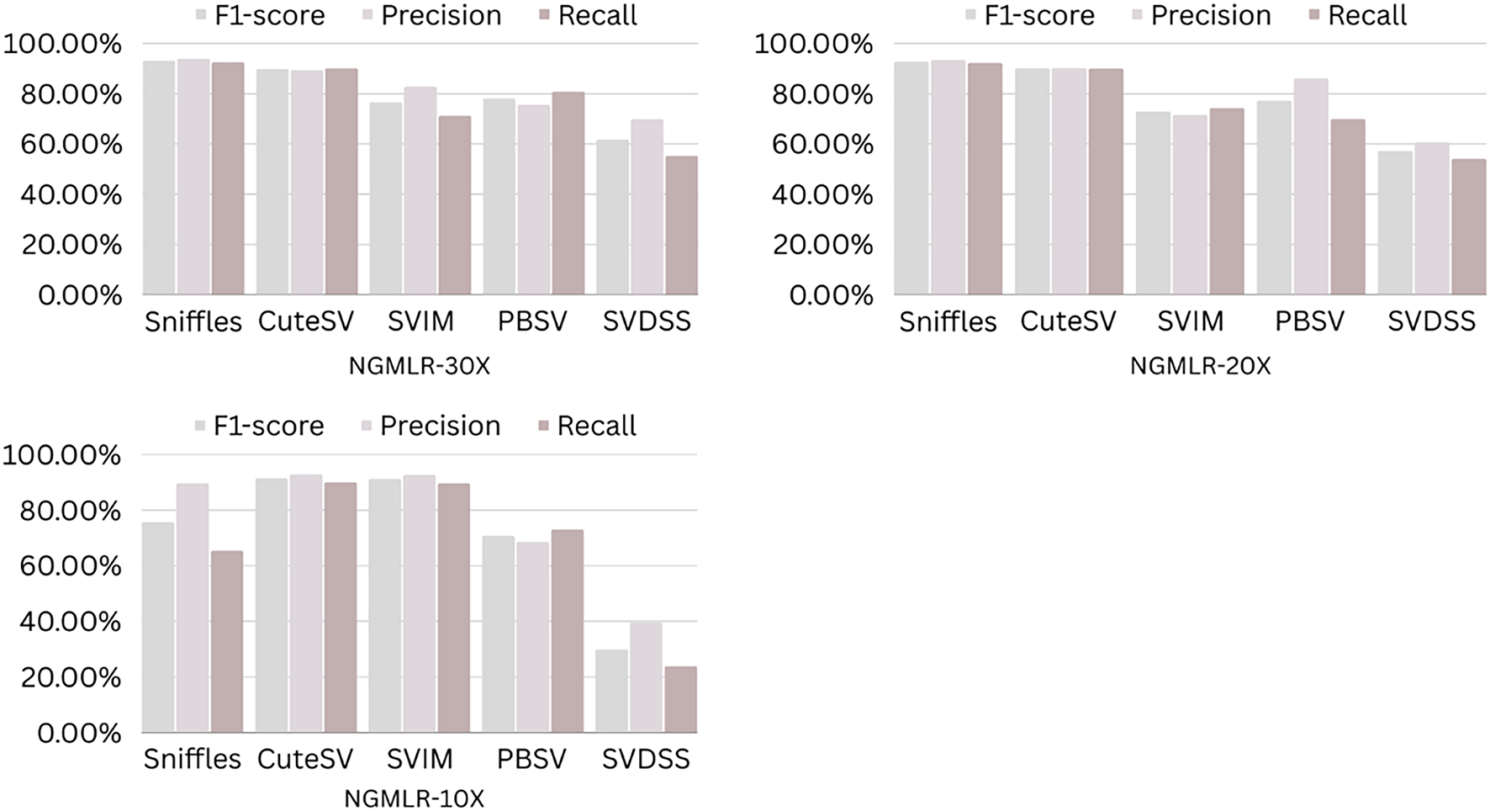
The F1-score, Precision and Recall (Y-axis) of different variant callers (Sniffles, CuteSV, SVIM, PBSV and SVDSS) with NGMLR aligner for the NA 12,878 dataset at different sequencing coverages.

**Figure 5:**
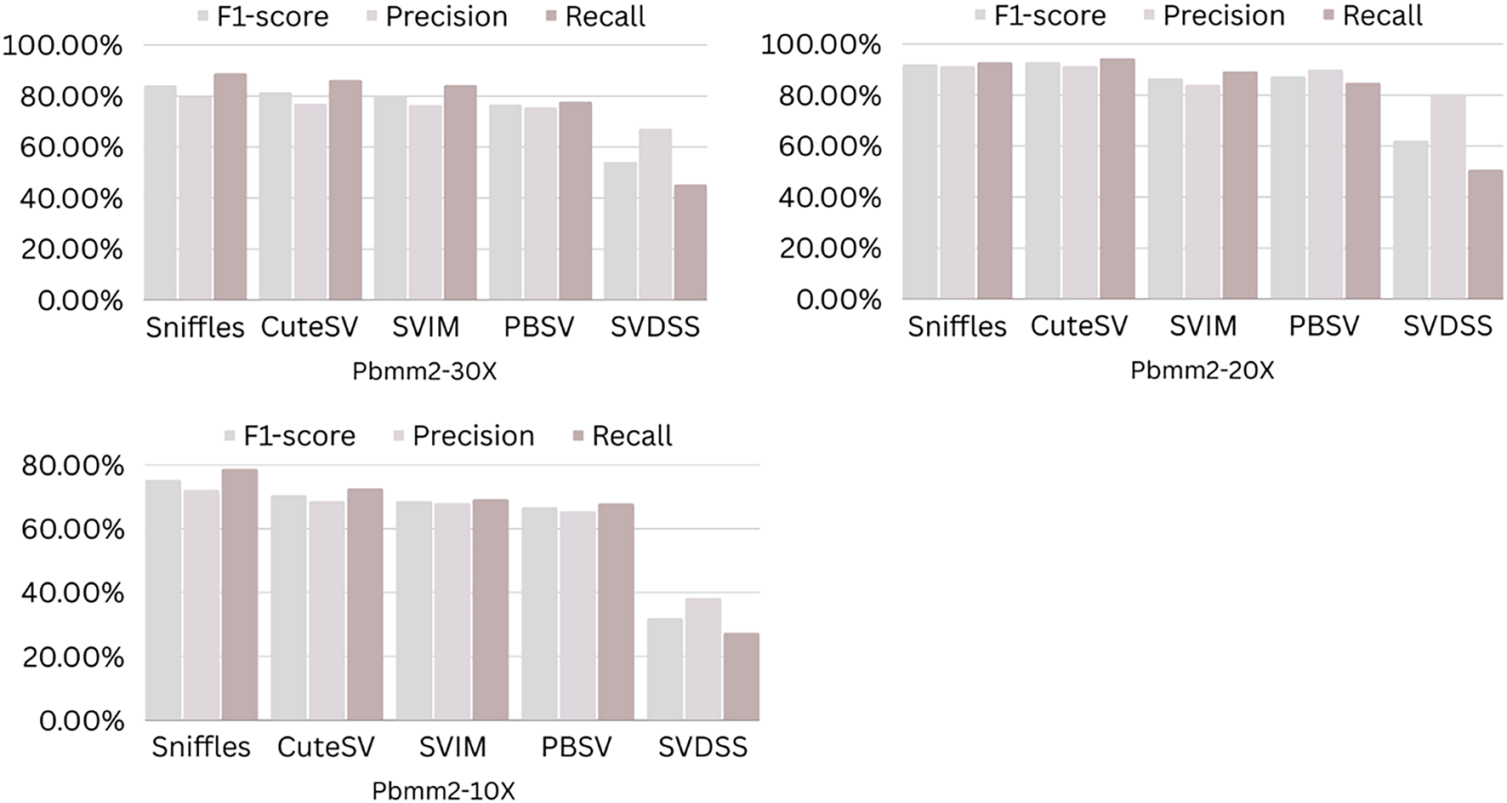
The F1-score, Precision and Recall (Y-axis) of different variant callers (Sniffles, CuteSV, SVIM, PBSV and SVDSS) with Pbmm2 aligner for the NA 12,878 dataset at different sequencing coverages.

**Figure 6:**
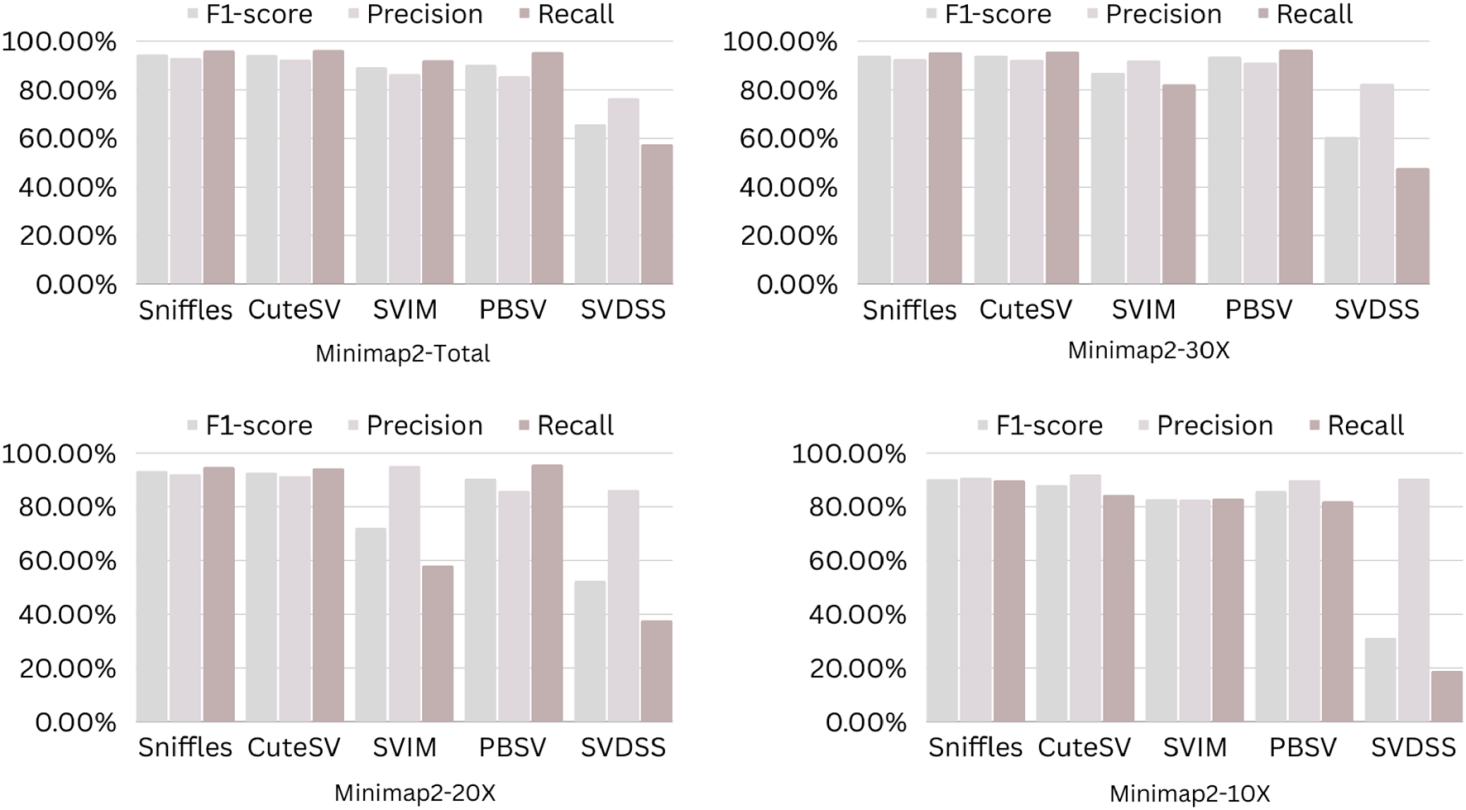
The F1-score, Precision and Recall (Y-axis) of different variant callers (Sniffles, CuteSV, SVIM, PBSV and SVDSS) with Minimap2 aligner for the NA24385 dataset at different sequencing coverages.

**Figure 7:**
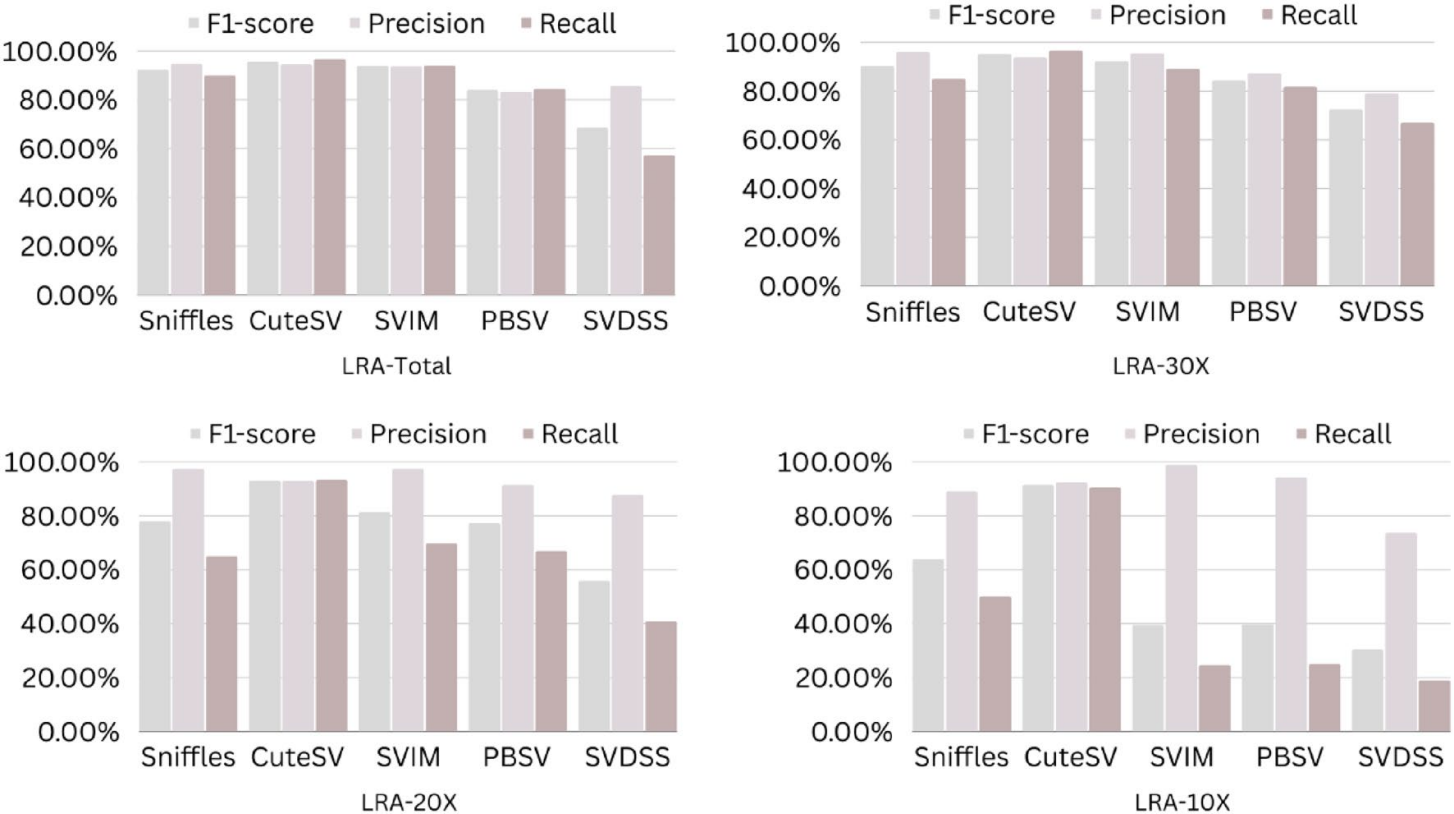
The F1-score, precision and recall (Y-axis) of different variant callers (Sniffles, CuteSV, SVIM, PBSV and SVDSS) with LRA aligner for the NA24385 dataset at different sequencing coverages.

**Figure 8:**
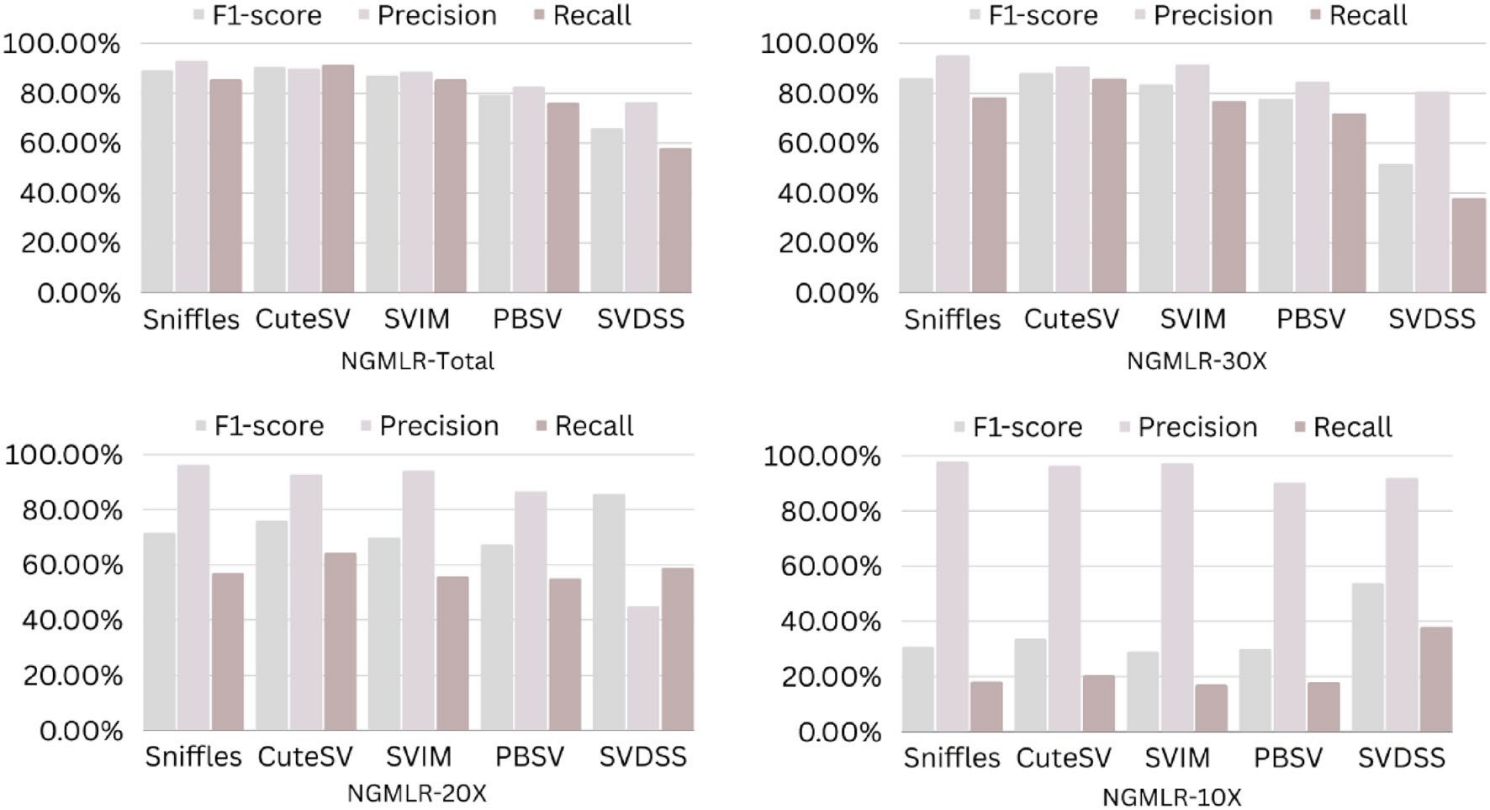
The F1-score, precision and recall (Y-axis) of different variant callers (Sniffles, CuteSV, SVIM, PBSV and SVDSS) with NGMLR aligner for the NA24385 dataset at different sequencing coverages.

**Figure 9:**
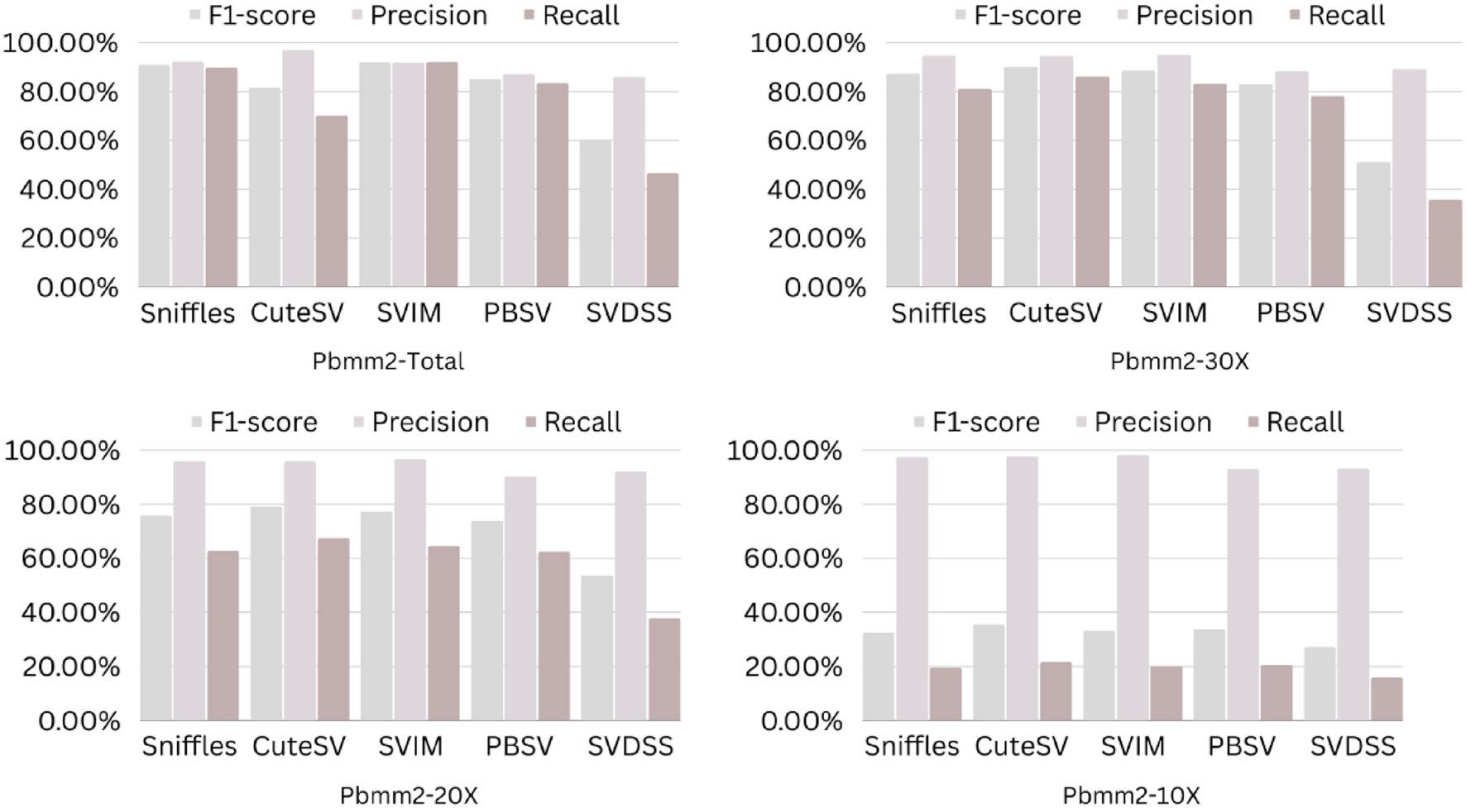
The F1-score, precision and recall (Y-axis) of different variant callers (Sniffles, CuteSV, SVIM, PBSV and SVDSS) with Pbmm2 aligner for the NA24385 dataset at different sequencing coverages.

**Figure 10:**
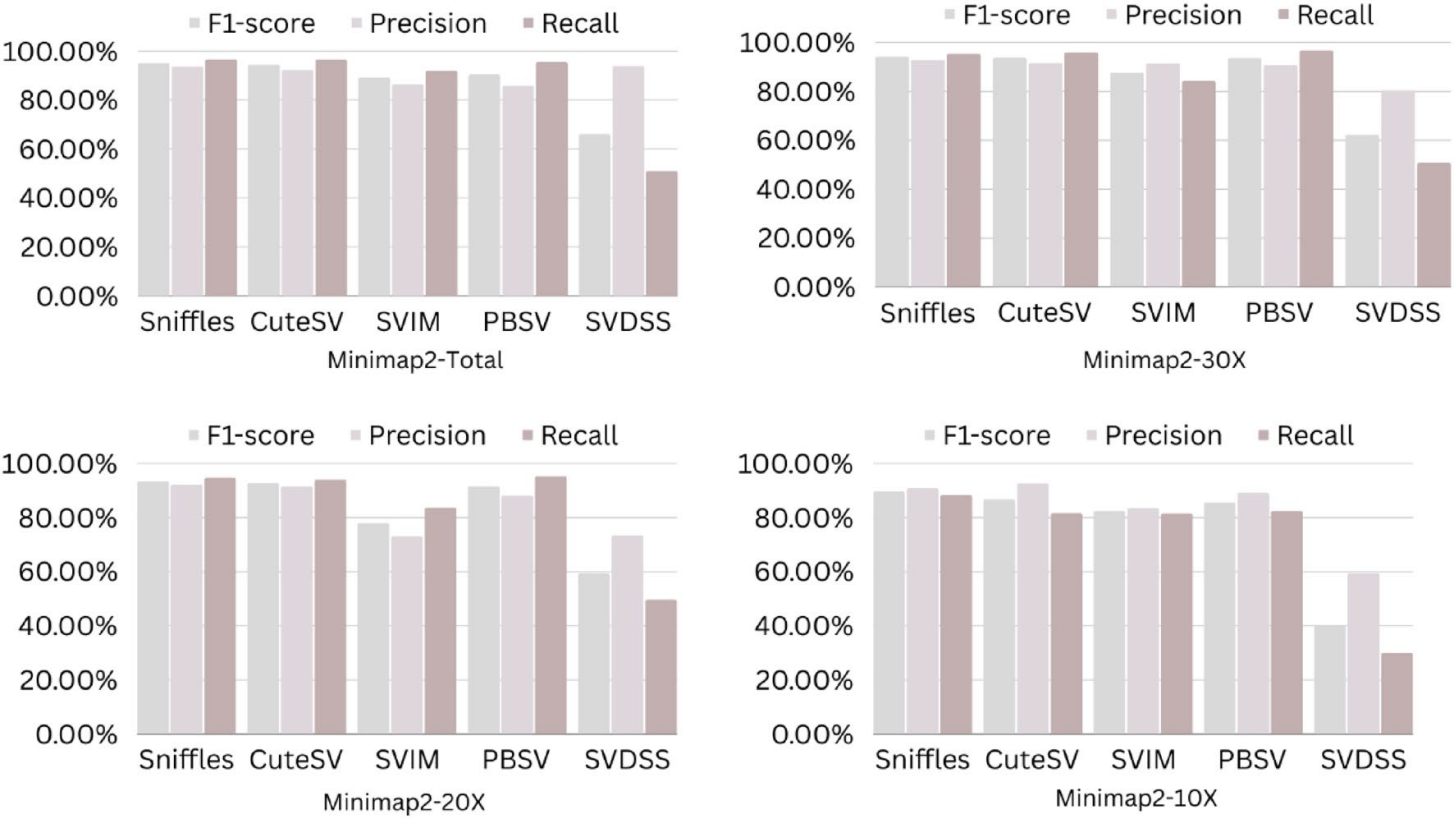
The F1-score, precision and recall (Y-axis) of different variant callers (Sniffles, CuteSV, SVIM, PBSV and SVDSS) with Minimap2 aligner for the SI00001 dataset at different sequencing coverages.

**Figure 11:**
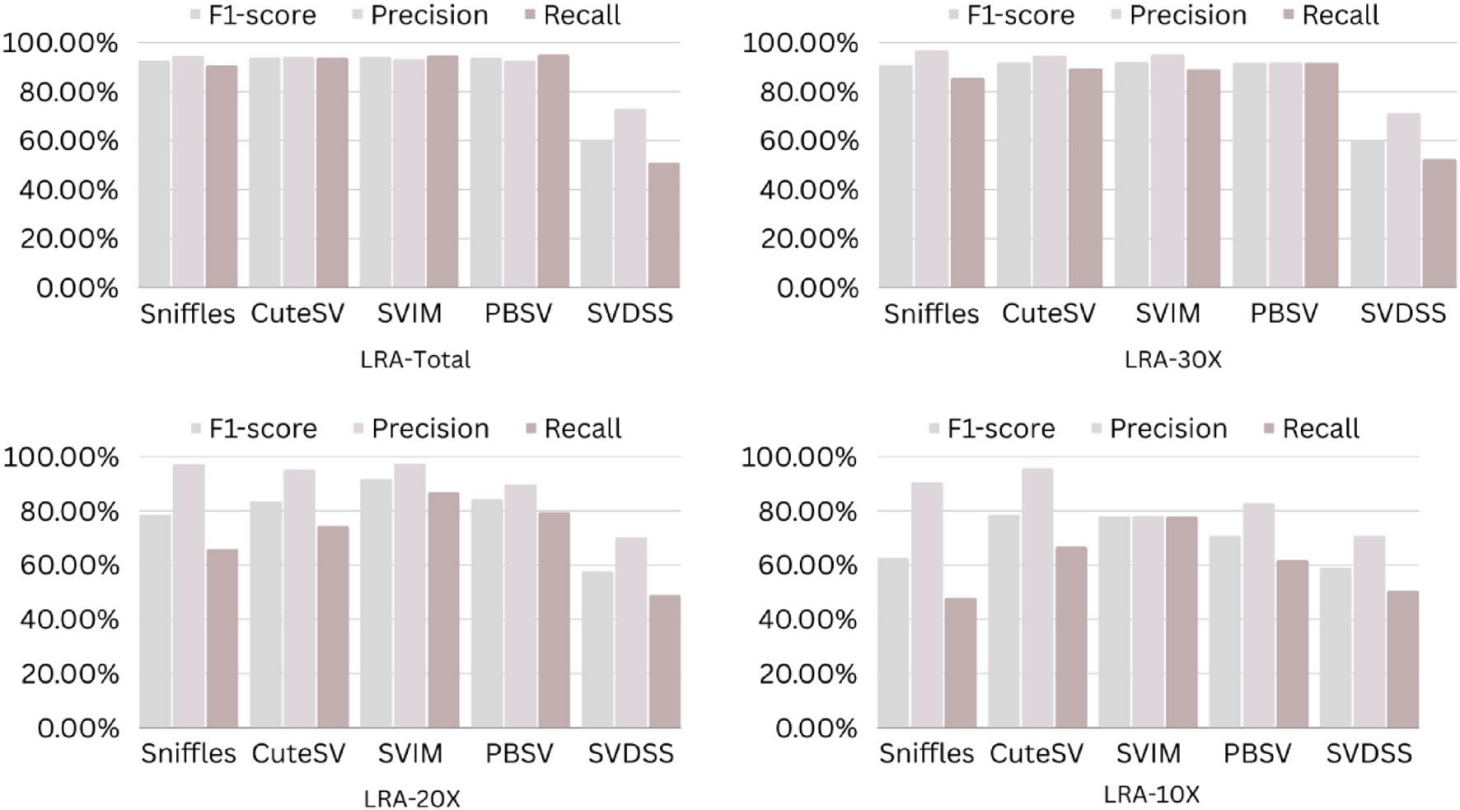
The F1-score, precision and recall (Y-axis) of different variant callers (Sniffles, CuteSV, SVIM, PBSV and SVDSS) with LRA aligner for the SI00001 dataset at different sequencing coverages.

**Figure 12:**
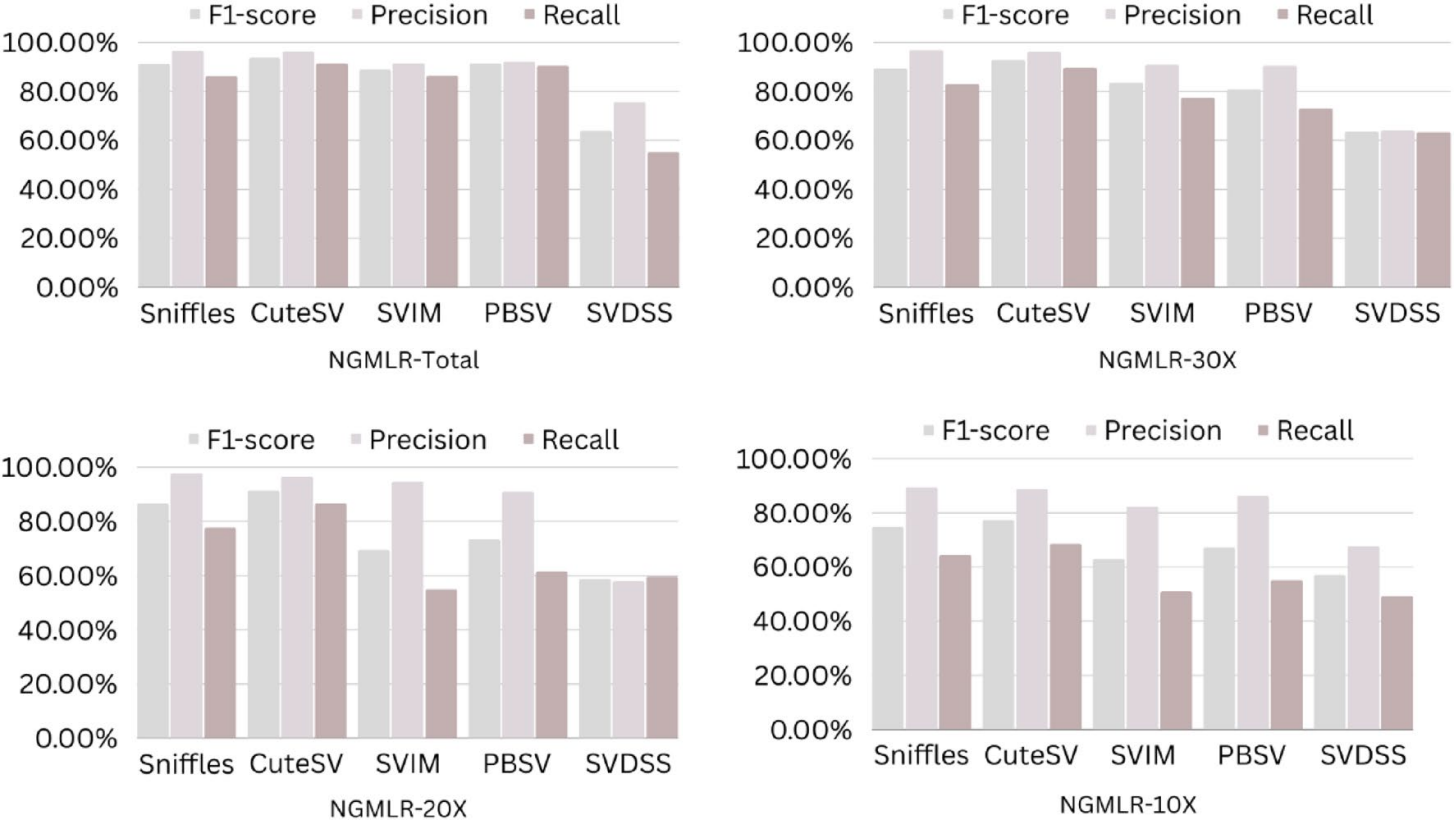
The F1-score, precision and recall (Y-axis) of different variant callers (Sniffles, CuteSV, SVIM, PBSV and SVDSS) with NGMLR aligner for the SI00001 dataset at different sequencing coverages.

**Figure 13:**
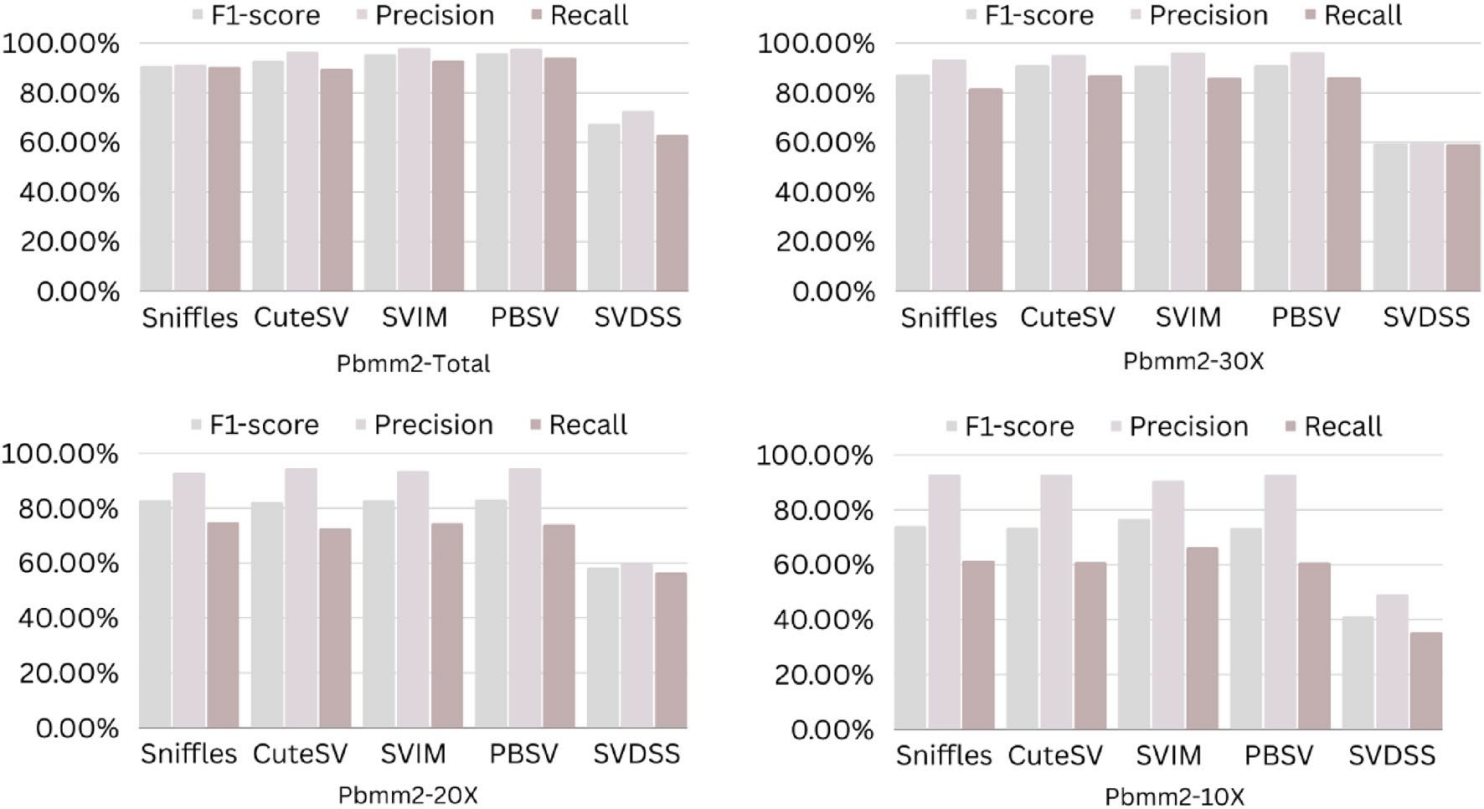
The F1-score, precision and recall (Y-axis) of different variant callers (Sniffles, CuteSV, SVIM, PBSV and SVDSS) with Pbmm2 aligner for the SI00001 dataset at different sequencing coverages.

The precision, recall, and F-score values for SV calling (Sniffles, SVIM, CuteSV, and PBSV) following Minimap2, LRA, NGMLR, and Pbmm2 alignments at different depths of coverages are displayed in Tables 4, 5 and 6; for the NA12878 (Figs. 2, 3, 4, 5), NA24385 (Figs. 6, 7, 8, 9) and simulated SI00001 (Figs. 10, 11, 12, 13) human genome datasets, respectively. The benchmarking results for the three reference datasets, combined with four different long-read aligners (Minimap2, LRA, pbmm2, and NGMLR) and four different structural variant callers (CuteSV, Sniffles, PBSV, and SVIM), revealed that the SV caller performance varies depending on the dataset and the specific SV types being analyzed. It was also revealed that the average F1 score increased with sequencing coverage, and that Sniffles and CuteSV tend to perform well across different aligners and coverage levels, followed by SVIM, PBSV, and SVDSS in last place. The CuteSV caller has the highest average F1 score (82.51%) and recall (78.50%) of the five SV callers. Also, CuteSV scored the second-highest average precision value (78.50%), showing that it can recognize a high percentage of actual SVs while minimizing false positives. The Sniffles caller closely follows the average scores of CuteSV; it has the highest average precision value (94.33%), the second-highest average F1-score (78.88%), and Recall (72.47%) of the five SV callers. The Sniffles caller may overlook actual SVs due to its lower average recall value than the CuteSV caller. In third place, after CuteSV and Sniffles, comes SVIM which has the third-highest average F1-score (75.02%) and precision (93.52%) among the five SV callers; however, the average Recall (68.10%) is lower than that of the CuteSV and Sniffles callers. The SVIM caller may overlook certain SVs but has a low false positive rate. Furthermore, PBSV has a lower average F1-score (73.55%), precision (88.30%), and recall (68.42%) than the top three SV callers. This shows that the PBSV caller may generate more false positives and overlook some actual SVs. The SVDSS caller's average F1-score (55.49%) and recall (42.28%) are the lowest of the five SV callers, suggesting it may miss a lot of actual SVs. The SVDSS caller has a high precision value (82.33%), indicating few false positives.

**Table 4:** The precision, recall, and F-score values for SV calling for the NA12878 sample with Sniffles, SVIM, CuteSV, PBSV and SVDSS following Alignment with the four evaluated aligners Minimap2, LRA, ngmlr and pbmm2 at different depths of coverage.

Aligner	Coverage	SV caller	Total No. of SV	F1-score (%)	Precision (%)	Recall (%)
Minimap2	30X	Sniffles	17,532	94.48	93.05	95.94
		CuteSV	14,266	92.03	92.09	91.96
		SVIM	17,699	89.35	86.62	92.26
		PBSV	10,251	88.37	85.99	90.89
		SVDSS	47,176	66.17	93.99	51.06
	20X	Sniffles	7761	92.17	91.37	92.97
		CuteSV	8224	92.96	91.44	94.53
		SVIM	7006	86.69	84.13	89.41
		PBSV	6829	87.40	90.01	84.93
		SVDSS	33,896	62.25	80.34	50.81
	10X	Sniffles	1261	91.29	91.51	91.06
		CuteSV	1260	89.96	91.18	88.77
		SVIM	1036	77.58	76.25	78.95
		PBSV	1130	87.11	90.24	84.18
		SVDSS	15,406	39.28	67.57	27.68
LRA	30X	Sniffles	18,875	95.19	97.06	93.40
		CuteSV	13,732	91.97	93.93	90.08
		SVIM	18,315	85.17	93.79	78.00
		PBSV	12,208	79.25	77.21	81.41
		SVDSS	57,893	58.87	68.33	51.71
	20X	Sniffles	8006	83.63	92.28	76.46
		CuteSV	7749	91.27	93.90	88.77
		SVIM	7338	95.19	96.92	93.52
		PBSV	7576	75.74	73.82	77.77
		SVDSS	45,597	59.67	69.09	59.67
	10X	Sniffles	1352	78.73	91.83	68.90
		CuteSV	1170	89.48	92.96	86.25
		SVIM	1052	94.86	96.08	93.68
		PBSV	1249	71.77	70.96	72.61
		SVDSS	23,935	31.34	40.05	25.74
NGMLR	30X	Sniffles	14,552	93.25	93.95	92.56
		CuteSV	16,821	89.80	89.46	90.14
		SVIM	15,399	76.59	82.83	71.23
		PBSV	9931	78.16	75.66	80.83
		SVDSS	49,919	61.81	69.95	55.36
	20X	Sniffles	5903	92.85	93.43	92.27
		CuteSV	6879	90.14	90.25	90.02
		SVIM	6076	72.98	71.66	74.36
		PBSV	5850	77.30	86.20	70.06
		SVDSS	38,452	57.27	60.82	54.12
	10X	Sniffles	734	75.70	89.69	65.49
		CuteSV	962	91.47	92.95	90.04
		SVIM	844	91.15	92.72	89.63
		PBSV	971	70.81	68.63	73.12
		SVDSS	19,215	29.92	39.70	24.00
Pbmm2	30X	Sniffles	17,319	84.19	79.90	88.97
		CuteSV	19,190	81.47	77.04	86.43
		SVIM	18,176	80.28	76.55	84.40
		PBSV	10,439	76.75	75.67	77.86
		SVDSS	49,919	54.23	67.33	45.40
	20X	Sniffles	7328	80.58	76.28	85.38
		CuteSV	7795	77.61	74.03	81.55
		SVIM	7087	76.96	73.81	80.38
		PBSV	6769	74.27	72.69	75.92
		SVDSS	38,452	50.84	57.80	45.37
	10X	Sniffles	1062	75.41	72.22	78.89
		CuteSV	1124	70.67	68.74	72.71
		SVIM	1018	68.76	68.15	69.38
		PBSV	1162	66.75	65.55	67.99
		SVDSS	19,215	32.04	38.46	27.46

**Table 5:** The precision, recall, and F-score values for SV calling for the NA24385 sample with Sniffles, SVIM, CuteSV, PBSV and SVDSS following Alignment with the four evaluated aligners Minimap2, LRA, ngmlr and pbmm2 at different depths of coverage.

Aligner	Coverage	SV caller	Total No. of SV	F1-score (%)	Precision (%)	Recall (%)
Minimap2	Total	Sniffles	22,524	94.70	93.21	96.25
		CuteSV	21,182	94.43	92.54	96.40
		SVIM	44,508	89.33	86.52	92.32
		PBSV	19,572	90.37	85.59	95.72
		SVDSS	58,345	65.82	76.63	57.68
	30X	Sniffles	18,330	94.17	92.74	95.64
		CuteSV	19,608	94.15	92.46	95.90
		SVIM	33,690	87.04	92.19	82.44
		PBSV	17,976	93.79	91.27	96.46
		SVDSS	44,195	60.69	82.67	47.95
	20X	Sniffles	13,088	93.51	92.26	94.80
		CuteSV	14,292	92.85	91.44	94.30
		SVIM	21,945	72.28	95.25	58.23
		PBSV	14,572	90.58	85.98	95.72
		SVDSS	41,901	52.55	86.27	37.78
	10X	Sniffles	4058	90.41	90.92	89.91
		CuteSV	2580	88.14	92.16	84.46
		SVIM	5701	82.97	82.72	83.22
		PBSV	6800	85.87	90.00	82.10
		SVDSS	32,374	31.30	90.65	18.92
LRA	Total	Sniffles	21,660	92.41	94.84	90.10
		CuteSV	21,222	95.72	94.63	96.83
		SVIM	21,592	94.02	93.85	94.19
		PBSV	20,695	83.97	83.37	84.58
		SVDSS	48,534	68.67	85.70	57.27
	30X	Sniffles	19,050	90.31	96.14	85.16
		CuteSV	19,535	95.18	93.72	96.69
		SVIM	19,043	92.30	95.55	89.26
		PBSV	18,836	84.49	87.38	81.79
		SVDSS	19,394	72.60	79.06	67.12
	20X	Sniffles	13,774	78.04	97.48	65.07
		CuteSV	14,234	93.19	93.02	93.36
		SVIM	13,493	81.34	97.53	69.75
		PBSV	14,145	77.34	91.49	66.98
		SVDSS	19,505	55.90	87.80	41.00
	10X	Sniffles	4455	64.10	88.97	50.09
		CuteSV	4573	91.45	92.40	90.53
		SVIM	4242	39.55	99.04	24.71
		PBSV	4750	39.76	94.40	25.18
		SVDSS	31,196	30.31	73.77	19.07
NGMLR	Total	Sniffles	18,215	89.24	93.19	85.60
		CuteSV	20,638	90.64	89.89	91.40
		SVIM	18,502	87.12	88.69	85.59
		PBSV	19,577	79.36	82.72	76.26
		SVDSS	62,482	66.05	76.46	58.13
	30X	Sniffles	15,483	85.97	95.10	78.44
		CuteSV	17,095	88.23	90.80	85.80
		SVIM	15,625	83.63	91.54	76.97
		PBSV	17,345	77.82	84.67	71.99
		SVDSS	37,876	51.84	80.67	38.20
	20X	Sniffles	10,655	71.63	96.29	57.03
		CuteSV	11,944	76.04	92.82	64.39
		SVIM	10,537	70.04	94.20	55.74
		PBSV	12,218	67.42	86.71	55.15
		SVDSS	42,354	85.80	44.96	59.00
	10X	Sniffles	3006	30.73	97.94	18.22
		CuteSV	3296	33.75	96.43	20.45
		SVIM	2860	29.06	97.28	17.08
		PBSV	3433	30.04	90.33	18.02
		SVDSS	22,160	53.78	92.01	37.99
Pbmm2	Total	Sniffles	21,198	90.98	92.27	89.73
		CuteSV	20,649	81.53	97.09	70.26
		SVIM	21,942	92.07	91.92	92.21
		PBSV	20,645	85.29	87.18	83.49
		SVDSS	24,017	60.50	85.98	46.66
	30X	Sniffles	17,736	87.36	94.66	81.10
		CuteSV	19,209	90.17	94.61	86.12
		SVIM	17,805	88.73	95.06	83.20
		PBSV	18,183	82.96	88.49	78.07
		SVDSS	30,740	51.20	89.20	35.90
	20X	Sniffles	13,037	75.92	95.97	62.79
		CuteSV	14,126	79.13	95.81	67.40
		SVIM	12,802	77.41	96.75	64.52
		PBSV	13,709	73.89	90.30	62.54
		SVDSS	21,532	53.65	92.10	37.82
	10X	Sniffles	3776	32.59	97.37	19.57
		CuteSV	4078	35.49	97.66	21.69
		SVIM	3606	33.37	98.23	20.10
		PBSV	4102	33.78	92.99	20.64
		SVDSS	8500	27.27	93.28	15.97

**Table 6:** The precision, recall, and F-score values for SV calling for the SI00001 sample with Sniffles, SVIM, CuteSV, PBSV and SVDSS following Alignment with the four evaluated aligners Minimap2, LRA, ngmlr and pbmm2 at different depths of coverages.

Aligner	Coverage	SV caller	Total No. of SV	F1-score (%)	Precision (%)	Recall (%)
minimap2	Total	Sniffles	9756	95.22	93.79	96.70
		CuteSV	8971	94.49	92.40	96.68
		SVIM	9164	89.23	86.60	92.01
		PBSV	4604	90.52	85.95	95.60
		SVDSS	6909	66.17	93.99	51.06
	30X	Sniffles	7092	94.14	92.94	95.37
		CuteSV	7266	93.76	91.64	95.98
		SVIM	6764	87.67	91.47	84.17
		PBSV	2932	93.68	90.80	96.75
		SVDSS	4223	62.25	80.34	50.81
	20X	Sniffles	3240	93.46	92.14	94.81
		CuteSV	3480	92.77	91.57	93.99
		SVIM	3063	78.00	73.06	83.66
		PBSV	1080	91.61	88.21	95.30
		SVDSS	2494	59.33	73.50	49.74
	10X	Sniffles	292	89.63	90.87	88.42
		CuteSV	328	86.75	92.66	81.56
		SVIM	242	82.45	83.43	81.49
		PBSV	110	85.70	89.26	82.41
		SVDSS	868	39.96	59.53	30.07
LRA	Total	Sniffles	10,122	92.61	94.58	90.72
		CuteSV	9215	93.96	94.11	93.80
		SVIM	9280	94.07	93.36	94.80
		PBSV	8550	93.80	92.55	95.10
		SVDSS	11,011	60.04	73.04	50.97
	30X	Sniffles	7633	90.89	96.95	85.55
		CuteSV	7043	92.00	94.66	89.49
		SVIM	7044	92.03	95.10	89.16
		PBSV	6789	91.91	91.94	91.87
		SVDSS	7595	60.41	71.25	52.44
	20X	Sniffles	2677	78.63	97.26	65.99
		CuteSV	3244	83.57	95.35	74.39
		SVIM	3252	91.80	97.39	86.81
		PBSV	3261	84.34	89.77	79.52
		SVDSS	5228	57.73	70.24	49.00
	10X	Sniffles	305	62.71	90.67	47.93
		CuteSV	362	78.66	95.74	66.76
		SVIM	255	78.02	78.14	77.90
		PBSV	294	70.78	82.81	61.81
		SVDSS	2184	59.10	70.97	50.64
NGMLR	Total	Sniffles	9256	91.16	96.67	86.25
		CuteSV	8972	93.77	96.31	91.36
		SVIM	8527	88.95	91.51	86.52
		PBSV	8431	91.43	92.24	90.62
		SVDSS	7480	63.96	75.70	55.38
	30X	Sniffles	7207	89.44	96.95	83.02
		CuteSV	7104	92.78	96.17	89.62
		SVIM	6286	83.65	90.93	77.45
		PBSV	6587	80.90	90.56	73.10
		SVDSS	4519	63.72	64.04	63.41
	20X	Sniffles	3591	86.63	97.89	77.69
		CuteSV	3548	91.43	96.66	86.74
		SVIM	2806	69.60	94.73	55.01
		PBSV	2438	73.44	90.95	61.59
		SVDSS	2835	58.80	57.92	59.70
	10X	Sniffles	163	74.88	89.49	64.37
		CuteSV	181	77.41	88.84	68.59
		SVIM	132	63.00	82.27	51.04
		PBSV	159	67.27	86.31	55.11
		SVDSS	1122	57.13	67.81	49.36
Pbmm2	Total	Sniffles	2470	90.88	91.25	90.52
		CuteSV	3973	93.00	96.61	89.65
		SVIM	3806	95.52	98.11	93.07
		PBSV	3102	95.95	97.80	94.16
		SVDSS	1895	67.59	72.71	63.14
	30X	Sniffles	1541	87.26	93.54	81.78
		CuteSV	2294	91.03	95.29	87.13
		SVIM	2047	90.90	96.28	86.09
		PBSV	2327	91.03	96.37	86.25
		SVDSS	1020	59.59	59.88	59.31
	20X	Sniffles	468	82.99	92.99	74.93
		CuteSV	706	82.26	94.67	72.73
		SVIM	571	82.94	93.54	74.50
		PBSV	722	83.14	94.65	74.13
		SVDSS	527	58.35	60.25	56.56
	10X	Sniffles	35	74.12	92.98	61.62
		CuteSV	53	73.63	92.84	61.01
		SVIM	39	76.75	90.74	66.50
		PBSV	59	73.53	92.89	60.84
		SVDSS	172	41.19	49.15	35.45

On average, the CuteSV caller has a CPU time of 4.044 h, a wall clock time of 102.3 min, and a memory usage of 3.4 GB across all aligners. The CuteSV caller relies on high-quality alignments to reliably call structural variations, which may affect its performance. It performs well across aligners and uses little CPU and memory. In addition, Sniffles has a CPU time of 4.227 h, a wall clock time of 121.3 min, and a memory usage of 5.1 GB across all aligners. Like CuteSV, Sniffles tends to perform relatively well across all aligners. SVIM's CPU time was 3.445 h, wall clock time was 463.4 min, and memory use was 3.405 GB. The two-step PBSV variant calling process has an average CPU time of 11.81 h and a wall clock time of 336.1 min, with a memory usage of 56.91 GB across all aligners. It is explicitly designed for PacBio long-read data and can be computationally intensive. The three-step SVDSS variant calling process takes an average of 16.183 h on the CPU and 4:01:15 on the wall clock, and memory usage of 70.723 GB across all aligners (Table 3).

### Evaluation of the different SV callers’ performance against the three datasets in terms of deletions and insertions

Each SV caller called different kinds of SVs in different numbers,, the most common types being deletions and insertions. Because only a small number of SV types other than insertions and deletions were called and some SV true sets only have insertions and deletions, the resulting SV calls from all SV callers were put into two main groups: deletions (DEL) and insertions (INS). The current evaluation did not use other types of SVs in the call sets, like inversions and translocations. The two callers, SVDSS and SVIM, consistently called a higher number of SVs than the other callers and tended to have a higher proportion of both deletions and insertions, and this may explain the F1-scores, precision, and recall values for these two tools. Sniffles and CuteSV tended to call fewer SVs than SVDSS and the SVIM. PBSV called the least number of SVs across all aligners and levels of coverage, which may be due to it being designed for analyzing PacBio long-read data. The results for using NpInv on the three datasets at different coverage degrees revealed that the number of inversions called by the NpInv tool increases with higher levels of coverage, which is expected given the increased sequencing depth and information available at higher coverage levels (Supplementary Table S1–S3). The results also suggest that the choice of aligner can impact the performance of NpInv. However, the differences in performance between the aligners are relatively small, and NpInv appeared to perform well with all the aligners tested. In terms of coverage level, the highest number of inversions was called at the 30X coverage level, followed by the 20X and 10X levels. The same trend in the three datasets indicated that the degree of coverage highly impacts NpInv (Supplementary Table S4–S6).

### Evaluation of different SV callers’ performance in terms of SV length and their performance in terms of precision, recall and F1-score

In order to comply with the definition of a structural variant, all the SVs that were less than 50 bp were disregarded and filtered-out in the filtration step. The SV count in each group was presented in detail with the demonstration for the SV distribution across different SV length ranges in supplementary tables (Supplementary S7–S9). In general, CuteSV detected a significant number of SVs in the 50–250 bp range but none in the < 50 bp range. SVIM detected a large number of SVs in the 50–250 bp range and also had substantial detection in the < 50 bp range. PBSV showed consistent detection in the 50–250 bp and 251–500 bp ranges. SVDSS had the highest total number of SVs detected, with a significant number in the < 50 bp and 50–250 bp ranges. At the Total coverage: Sniffles detected the lowest total number of SVs (< 50 bp) and the highest number of SVs in the 50–250 bp range. CuteSV detected a significant number of SVs in the 50–250 bp range but none in the < 50 bp range. SVIM detected a large number of SVs in the 50–250 bp range and also had substantial detection in the < 50 bp range. PBSV showed consistent detection in the 50–250 bp and 251–500 bp ranges. SVDSS had the highest total number of SVs detected, with a significant number in the < 50 bp and 50–250 bp ranges.

At 30X coverage: Sniffles has a high number of detected variants in the 50–250 bp range followed by 251–500 bp and 501–750 bp ranges. CuteSV detected more variants in the 50–250 bp range, with very few in other ranges. SVIM has a significant detection rate in the < 50 range, followed by the 50–250 bp range. PBSV also has most variants in the 50–250 bp range, with fewer detected as the length increases. SVDSS has a very high number in the < 50 bp range, followed by a substantial count in the 50–250 bp range. At 20X coverage: Sniffles, PBSV, CuteSV, and SVIM generally show similar patterns as seen in 30X coverage, with overall lower counts, SVDSS still remains notably high in the < 50 bp range and lower in higher ranges. At 10X coverage: Sniffles detected a significantly reduced number of variants in all ranges compared to 30X coverage. CuteSV detected fewer variants across all ranges, with zero in the < 50 bp range. SVIM detected a notably high count in the < 50 bp range with a steep drop-off in larger sizes. PBSV again shows a similar pattern with a preference towards the 50–250 bp range. SVDSS still detected a substantial number in the < 50 bp range, markedly more than other callers at this coverage (Supplementary S7–S9). The distribution and the count of the detected SVs in terms of SV length groups were charted into bar charts to give insights about the performance of the different variant callers’ vs number of SVs detected per length range for NA12878 (Supplementary Figures S1–S3), NA24385 (Supplementary Figures S4–S7) and SI00001 (Supplementary Figures S8–S11) datasets.

The accuracy metrics in terms of precision, recall and F1-score across the different SV length groups were applied to the most commonly studied reference sample NA24385 as this will be valuable towards future studies and evaluation. For Minimap2 Total Coverage: Sniffles showed varying performance across different SV length groups, with precision ranging from 47.01 to 72.80% and recall ranging from 38.14 to 77.21%. The F1-score ranged from 42.11 to 73.28%, indicating variability in its performance across different SV length categories.

CuteSV demonstrated consistently high precision, recall, and F1-score across all SV length groups, with values ranging from 82.98 to 94.73% for precision, 94.63–97.45% for recall, and 88.71–95.03% for F1-score. This indicates strong and consistent performance in detecting SVs across different length categories at this coverage level. SVIM showed varying performance, with precision ranging from 56.19 to 83.02%, recall ranging from 65.91 to 81.09%, and F1-score ranging from 60.66 to 76.24% across different SV length groups. PBSV demonstrated relatively high precision, recall, and F1-score across different SV length groups, indicating consistent performance in detecting SVs of varying lengths at this coverage level.

For Minimap2 at 30X Coverage: SVDSS demonstrated varying performance across different SV length groups, with precision ranging from 69.77 to 93.25%, recall ranging from 70.35 to 81.94%, and F1-score ranging from 70.06 to 87.23%. Sniffles showed varying performance, with precision ranging from 65.82 to 81.12%, recall ranging from 61.22 to 79.00%, and F1-score ranging from 58.77% to 77.52%. CuteSV demonstrated consistently high precision, recall, and F1-score across all SV length groups, with values ranging from 93.89 to 96.64% for precision, 95.61–97.67% for recall, and 94.74–97.15% for F1-score. SVIM showed varying performance, with precision ranging from 65.97 to 88.91%, recall ranging from 69.86 to 77.76%, and F1-score ranging from 67.86 to 82.96%. PBSV demonstrated relatively high precision, recall, and F1-score across different SV length groups, indicating consistent performance in detecting SVs of varying lengths at 30X coverage.

For Minimap2 at 20X Coverage: SVDSS demonstrated varying performance across different SV length groups, with precision ranging from 78.23 to 98.46%, recall ranging from 77.87 to 94.74%, and F1-score ranging from 78.05 to 96.57%. Sniffles showed varying performance, with precision ranging from 76.53 to 88.70%, recall ranging from 74.79 to 85.26%, and F1-score ranging from 75.65 to 84.94%. CuteSV demonstrated consistently high precision, recall, and F1-score across all SV length groups, with values ranging from 95.76 to 99.47% for precision, 96.75–97.67% for recall, and 96.25–99.20% for F1-score. SVIM showed varying performance, with precision ranging from 79.20 to 91.72%, recall ranging from 79.19 to 84.71%, and F1-score ranging from 79.20 to 88.08%. PBSV demonstrated relatively high precision, recall, and F1-score across different SV length groups, indicating consistent performance in detecting SVs of varying lengths at 20X coverage.

For Minimap2 at 10X Coverage: SVDSS demonstrated varying performance across different SV length groups, with precision ranging from 90.60 to 99.73%, recall ranging from 90.15 to 99.19%, and F1-score ranging from 90.37 to 99.46%. Sniffles showed varying performance, with precision ranging from 93.13 to 97.05%, recall ranging from 79.65 to 94.97%, and F1-score ranging from 86.09 to 95.63%. CuteSV demonstrated consistently high precision, recall, and F1-score across all SV length groups, with values ranging from 98.92 to 99.47% for precision, 99.12–98.94% for recall, and 99.02–99.20% for F1-score. SVIM showed varying performance, with precision ranging from 95.08 to 99.65%, recall ranging from 94.79 to 98.88%, and F1-score ranging from 94.93 to 99.26%. PBSV demonstrated relatively high precision, recall, and F1-score across different SV length groups, indicating consistent performance in detecting SVs of varying lengths at 10X coverage.

The SV callers’ performance with LRA, NGMLR, and Pbmm2 was the same as with Minimap2 where CuteSV demonstrated consistently high precision, recall, and F1-score across all SV length groups and coverage levels, indicating strong and consistent performance in detecting SVs. Sniffles showed varying performance across different SV length groups and coverage levels, with competitive precision and recall for detecting SVs of various lengths. SVIM demonstrated competitive performance in detecting SVs of various lengths at different coverage levels, while PBSV exhibited relatively high precision, recall, and F1-score across different SV length groups, indicating consistent performance in detecting SVs at different coverage levels. As for SVDSS, it exhibited very varying performance, with relatively low precision, recall, and F1-score (Supplementary Tables S10–S13).

## Discussion

Most previous studies focused on single-nucleotide polymorphisms (SNPs) detection because they are easier to track down using existing sequencing tools and algorithms^39^. A well thought of prevalence of SV over the last 20 years has shifted our viewpoint on its impact on genomic disorders^40^. Despite all these indications of SV importance, they have received far less attention than SNVs due to their difficulty in detection. In theory, each type of SV produces a distinct outline in plotting reads that can be employed to deduce the basic variations^40^. Multiple SVs can be overlaid or grouped together, resulting in more intricate plotting shapes than when they are viewed separately. Such complex patterns may impede mapping entirely, imposing investigators to rebuild such genomic trials and analysis from scratch^27,41^.

With the introduction of long-read sequencing technology, specifically Pacific Biosciences (PacBio) and ONT, it has become possible to produce reads of thousand base pairs^19,29^. Because of different DNA library preparations, various platforms produce diverse kinds of information^42,43^. As previously reported, the primary distinctions between these types of reads are their length and error rate^44^. Furthermore, assembly-based methods can be utilized for SV detection. It is difficult to assess the performance of SV detection tools because of the absence of a reference scheme for precisely identifying such SVs.

To address this limitation, the Genome in a Bottle (GIAB) recently released a sequence-resolved benchmark set for SV detection^45^. We used the long-read nanopore sequencing data results for sample NA24385 deposited in NCBI ftp to produce an accurate archetypal for the assessment of the SV detection algorithms and to create our pipeline that can help SV detection by choosing the aligner and the SV caller that fits the results of an existing benchmark set available from GIAB^44,45^. The NA24385 and NA12878 samples FASTQ, after their retrieval from the NCBI repository and nanopore whole-genome sequencing consortium repository, as well as the simulated dataset SI00001 FASTQ as per the instructions provided in this repository (https://github.com/davidebolo1993/EViNCe/tree/main/SI00001 (accessed on 3 September 2023) were aligned to GRCh37 reference genome using four of the most common long-read aligners Minimap2, LRA, NGMLR and Pbmm2 a SMRT wrapper for Minimap2 developed for PacBio data. To evaluate the impact of sequencing depth on SV calls, subsets were created by down-sampling of the original dataset; each dataset was achieved at 30X, 20X, and 10X sequencing coverages by using Samtools, and using Truvari, benchmarking tool, we calculated the F1 score, precision, and Recall for each of the four studied SV callers at each coverage level. We put five general-purpose SV callers to the test: Sniffles^39^, SVIM^4,19^, CuteSV^30^, PBSV, and SVDSS^37^ as they can detect all SV types from long-read alignments with an exception for the SVDSS, which was developed to detect insertions and deletions only and not yet costumed to detect inversions. Currently, ONT recommends Sniffles2 as the go-to SV caller, which was integrated as the SV caller of choice for the variant detection pipeline, along with Clair3 for SNV/Indels detection.

The Sniffles2 caller detects all types of SVs and can be used with any aligner, particularly with Minimap2. As per the recommendation of ONT, this combination was used as the base of the two Nextflow based workflows to manage compute and software resources in various workflows as previously reported^46,47^. After mapping reads to the reference genome, the program detects split-reads and read-pairs that span the potential SV breakpoints. Sniffles2 clusters breakpoint-spanning reads and utilizes a probabilistic algorithm to identify the most likely SV type and breakpoints^39^, while the CuteSV caller collects SV signatures using customized approaches and analyzes them using a clustering-and-refinement process to find sensitive SVs. The CuteSV caller outperformed state-of-the-art techniques in yield and scalability on PacBio and ONT datasets. Furthermore, the CuteSV caller uses split-read and read-pair information to detect SVs. After mapping reads to the reference genome, the tool groups split-reads and read-pairs that support SV breakpoints. The CuteSV caller then uses graphs to determine the most likely SV type and breakpoints^30^.

Meanwhile, SVIM calls structural variants in third-generation sequencing reads, identify, and classify most of the genetic mutations or changes by integrating genome-wide data. SVIM uses de novo assembly to generate contigs spanning potential SV breakpoints. It outperformed competing approaches on simulated and real PacBio and nanopore sequencing data. It combines split-read and read-pair information with de novo insertion event assembly to identify SVs. The SV breakpoints were identified by mapping reads to the reference genome. SVIM then generates contigs spanning these breakpoints using a de novo assembler and aligns them to the reference genome to determine the most likely SV type and breakpoints^19^. PBSV is a variant calling software developed by PacBio to detect structural variants in long-read PacBio sequencing data. It aligns long reads to a reference genome using a long-read aligner and identifies structural variants using split-read; discordant read pairs indicate an SV. PBSV clusters discordant read pairs and finds the most likely SV type and breakpoints using a graph-based technique. PBSV clusters these variants and filters out false positives to identify complex and large structural variants that are hard to distinguish using short-read sequencing data (PacificBiosciences/pbsv, 2022). It is the most useful SV caller for detection of insertions ranging from 20 to 10 kb, deletions ranging from 20 to 100 kb, 200 bp to 10 kb inversions, and duplications ranging from 20 to 10 kb^44^. On the other hand, SVIM employs a graph-based technique to discover signature clusters and final SVs, with each node representing an SV signature, and is known to perform best with PacBio HiFi reads^13^. The PBSV’s precision of calling the SVs was much better than the recall across the different coverage datasets. Still, overall, its Recall and Precision were much lower than those reported by other tools. However, in other studies, its performance was better than Sniffles^19^. This may be due to a difference in the dataset and the aligner used for benchmarking and the aligner.

SVDSS is designed to identify SVs in hard-to-call genomic regions using long-read sequencing data and sample-specific strings. SVDSS requires a FASTA format reference genome for sample genotyping. It involves building an FMD index, smoothing the input BAM file, extracting SFS, assembling SFS into superstrings, and calling SVDSS to genotype SVs. It incorporates both split-read and soft-clipping analysis, clustering, and machine learning algorithms to improve accuracy^37^. Regarding Inversions, Inversions are structural variations where a segment of DNA is flipped so the sequence is reversed compared to the reference genome. NpInv is the tool of choice for detecting inversions from long-read sequencing data. It works by analyzing the alignment of long-read sequencing data to a reference genome^48^. NpInv uses a unique approach to detect inversions; It first identifies regions where the long-read sequencing data spans two regions of the reference genome in an orientation inconsistent with the reference genome. Then, it looks for a breakpoint, which is a location where the sequence in the long-read data abruptly changes orientation. Finally, NpInv uses a statistical model to determine whether the orientation change is consistent with an inversion^48^. NpInv is better than other inversion detection tools, such as SVIM, Sniffles, and CuteSV in several ways. Firstly, NpInv is designed specifically for detecting inversions, whereas other tools are designed to detect a broader range of structural variations. This means that Npinv is optimized for detecting inversions and may be more sensitive and specific for this type of structural variation^4,48^. Secondly, NpInv is designed to work with long-read sequencing data, which is typically more informative than short-read sequencing data. Long-read sequencing data allows NpInv to span the breakpoints of inversions, which can be challenging to detect with short-read sequencing data^48^.

Based on the results of the performance of different SV callers with Minimap2 aligner at different coverage depths, we can see that both Sniffles and CuteSV have the highest F1-scores across all coverage depths. The PBSV caller also has a high F1-score but with lower precision. SVIM has a lower F1-score than the other callers, especially at lower coverage depths. SVDSS has the lowest F1-score, precision, and recall at all coverage depths. All callers perform relatively well at higher coverage depths (30X and 20X) with F1-scores above 90%. However, at lower coverage depths (10X), all callers except Sniffles have lower F1-scores, with SVDSS having the lowest F1-score of only 31.3%.

Regarding the performance of different SV callers with LRA aligner at different coverage depths, we see that the CuteSV caller has the highest F1-score and recall at all coverage depths. The Sniffles caller has the highest precision but lower recall compared to the CuteSV caller. SVIM performs well with an F1-score above 90% at all coverage depths. PBSV has a relatively low F1-score and recall compared to the other callers. SVDSS has the lowest F1-score, precision, and recall at all coverage depths. All callers perform relatively well at higher coverage depths (30X and 20X) with F1-scores above 75%. However, at lower coverage depths (10X), all callers except CuteSV have lower F1-scores, with SVDSS having the lowest F1-score of only 30.31%.

The performance of different SV callers with NGMLR aligner at different coverage depths shows that the CuteSV caller has the highest F1-score and recall at all coverage depths. Sniffles has the highest precision but lower recall compared to the CuteSV caller. SVIM performs well with an F1-score above 80% at all coverage depths. PBSV has a relatively low F1-score and recall compared to the other callers. SVDSS has the lowest F1-score, precision, and recall at all coverage depths. All callers perform relatively well at higher coverage depths (30X and 20X) with F1-scores above 70%. However, at lower coverage depths (10X), all callers except CuteSV have lower F1-scores, with SVDSS having the lowest F1-score of only 53.78%.

The performance of different SV callers with Pbmm2 aligner at different coverage depths shows that SVIM has the highest F1-score, precision, and recall at all coverage depths. The CuteSV caller has a relatively low F1-score at all coverage depths but still performs better than Sniffles and PBSV. SVDSS has the lowest F1-score, precision, and recall at all coverage depths. All callers perform relatively well at higher coverage depths (30X and 20X) with F1-scores above 70%. However, at lower coverage depths (10X), all callers have lower F1-scores, with SVDSS having the lowest F1-score of only 27.27%.

After analyzing the precision, recall, and F1-score data of different variant callers coupled with Minimap2, LRA, NGMLR, and Pbmm2 aligners and with respect to the SV length, several trends and patterns emerge. CuteSV consistently demonstrates high precision, recall, and F1-score across all aligners, indicating its robust performance in detecting structural variants (SVs) across different length groups and coverage levels. Sniffles exhibits competitive performance with varying precision and recall, especially for larger SVs even though this particular variant caller was a top performer when testing on the unbinned reference. SVDSS consistently shows strong performance across aligners, with relatively high precision, recall, and F1-score at each SV length group even though it showed very poor performance when testing on the unbinned reference which also lays the groud for future investigation to this behavior. SVIM demonstrates competitive performance in detecting SVs of various lengths at different coverage levels. PBSV exhibits relatively high precision, recall, and F1-score across different SV length groups, indicating consistent performance in detecting SVs. In conclusion, CuteSV emerges as a top performer across all aligners, demonstrating consistent and robust performance in detecting SVs. Sniffles shows competitive performance, especially for larger SVs. SVIM demonstrates competitive performance, while PBSV exhibits relatively high precision and recall. These findings suggest that the choice of aligner and variant caller can significantly impact the accuracy and sensitivity of SV detection.

The percentages for recall and precision fluctuate with coverages as low as 10X, indicating that low coverages should not be included in structural variations calling routines, where 20X coverage appears to be the minimum coverage required to maintain the tools' performance as determined by the F1 score. The comparison metrics results proved the usual tendencies for higher sequencing depth to increase recall and precision, though these can be disproportional depending on the tool itself. More flexible thresholds boost recall but decrease precision, whereas tougher cut-offs do the opposite. The precision and recall rates of each form of SV were studied. Each method worked best for deletions and insertions, which comprise most SVs in the human genome. Based on the results presented in the paper, both Sniffles and CuteSV consistently perform well across different aligners and coverage depths in terms of F1-score, precision, and recall. Sniffles should be preferred if high precision is required, while the CuteSV caller and Sniffles should be selected if a high recall is needed. The Minimap2 aligner and Sniffles are recommended for preliminary analysis due to their great rapidity and stable performance for both insertions and deletions.

In summary, the best-performing SV caller depends on the aligner and coverage depth used. The CuteSV caller consistently performs well across different aligners and coverage depths, with high F1-scores and recall. Sniffles has high precision, but lower recall compared to CuteSV. SVIM performs well with high F1-scores, precision, and recall at all coverage depths with Pbmm2 aligner. PBSV has a relatively low F1-score and recall compared to other callers. SVDSS consistently has the lowest F1-score, precision, and recall at all coverage depths. Researchers should select the appropriate SV caller based on their specific data and research question, considering the aligner and coverage depth used. Recently, it was proposed as a possible approach to enhance the performance of the available SV callers and syndicate reads from multiple pipelines, such as from Sniffles, CuteSV, and SVIM, which can help reduce the overall false positive rate^3^. Researchers should select the appropriate SV caller based on their specific data and research question, considering the aligner and coverage depth used. Moreover, various studies have investigated and evaluated the available variant calling tools for Oxford nanopore sequencing in breast cancer^4,49^ as well as in the metagenome discovery of various secondary metabolites of various microorganisms^50,51^ as well as for the detection of various plant pathogens^52^.

## Conclusions

The current study highlights how different aligners and coverage levels affect the performance of various SV callers, with their performance varying depending on the dataset being analyzed. The choice of aligner can significantly impact the performance of structural variant (SV) callers, with Minimap2 outperforming NGMLR and LRA in recall, precision, and F1-score percentages, likely due to its ability to handle long reads. The lower coverage levels decrease SV callers’ performance due to fewer available reads. The Sniffles and CuteSV caller perform well across different aligners and coverage levels, accurately identifying various SV types. Both SVIM and PBSV perform well in some cases but have more variable performance, with SVIM having a lower recall and F1-scores and PBSV having high recall but lower precision at lower coverage levels. SVDSS consistently has the lowest F1-score, precision, and recall at all coverage depths. Based on the findings, the usage of SV callers such as the Sniffles or CuteSV are recommended for the preliminary data assessment because they achieve significant correctness, particularly upon evaluating low-coverage data. The Minimap2 as an aligner and Sniffles as an SV caller were chosen and suggested aligners as bases of the pipeline for SV calling because of their high speed and reasonable accomplishment when applying genomic mutation such as insertions and deletions. Overall, our study provides a comprehensive evaluation of popular SV callers and aligners. It can serve as a reference for researchers in selecting the most suitable tools for their SV detection needs.

### Supplementary Information

Supplementary Information.

## Data Availability

Data supporting the reported results are available in the main manuscript and in the supplementary file as well as on the following data repository: NA12878: https://github.com/nanopore-wgs-consortium/NA12878/blob/master/nanopore-human-genome/rel_3_4.md, Variant Call Format (VCF) used as a benchmark set https://ftp-trace.ncbi.nlm.nih.gov/giab/ftp/data/NA12878/NA12878_PacBio_MtSinai/NA12878.sorted.vcf.gz, bed file was extracted from the VCF file; NA24385: https://ftp.ncbi.nih.gov/giab/ftp/data/AshkenazimTrio/HG002_NA24385_son/; The truth set VCF file https://ftp-trace.ncbi.nlm.nih.gov/giab/ftp/data/AshkenazimTrio/analysis/NIST_SVs_Integration_v0.6/HG002_SVs_Tier1_v0.6.vcf.gz; and the corresponding be file https://ftp-trace.ncbi.nlm.nih.gov/giab/ftp/data/AshkenazimTrio/analysis/NIST_SVs_Integration_v0.6/HG002_SVs_Tier1_v0.6.bed; SI00001: the Dataset was simulated following the instructions in the following (Bolognini and Magi, 2021) (https://www.frontiersin.org/articles/10.3389/fgene.2021.761791/full) Github repository (https://github.com/AnkhBioinformatics/SVcallers_Comparisons).

## Abbreviations

aCGH: Array comparative genomic hybridization
BAM: Binary alignment map
Bp: Base pair
CNVs: Copy number variations
GB: Gigabytes
GIAB: Genome in a bottle
INV: Inversions
LRA: Long read aligner
NGS: Next generation sequencing
ONT: Oxford Nanopore Technology
SAM: Sequence alignment map
SNVs: Single nucleotide variants
SVs: Structural variations
VCF: Variant call format
VISOR: Variant SimulatOR
FMD-index: Frequency domain error bidirectional text index
SFS: Sample-specific strings
