# Supplementary material for: Benchmarking long-read aligners and SV callers for structural variation detection in Oxford nanopore sequencing data
Source: Sci Rep. 2024 Mar 14;14:6160. doi: 10.1038/s41598-024-56604-2 (PMC10940726; doi:10.1038/s41598-024-56604-2)
Supplement: Supplementary file 1 — Supplementary Information. [file 41598_2024_56604_MOESM1_ESM.docx]

**Supplementary Information**

**Table S1.** Summary for the SV count in terms of deletions and insertions for the NA12878 sample with different SV callers following the alignment with the four evaluated aligners at different depths of coverages.

| Aligner | Coverage | SV caller | Total | DEL | INS |
| --- | --- | --- | --- | --- | --- |
| Minimap2 | 30X | Sniffles | 17532 | 7974 | 9558 |
|  |  | CuteSV | 14266 | 6386 | 7880 |
|  |  | SVIM | 17699 | 8385 | 9314 |
|  |  | PBSV | 10251 | 4867 | 5384 |
|  |  | SVDSS | 47176 | 29249 | 17927 |
|  | 20X | Sniffles | 7761 | 3596 | 4165 |
|  |  | CuteSV | 8224 | 3699 | 4525 |
|  |  | SVIM | 7006 | 3623 | 3383 |
|  |  | PBSV | 6829 | 3359 | 3470 |
|  |  | SVDSS | 33896 | 21398 | 12498 |
|  | 10X | Sniffles | 1261 | 616 | 645 |
|  |  | CuteSV | 1260 | 618 | 642 |
|  |  | SVIM | 1036 | 587 | 449 |
|  |  | PBSV | 1130 | 629 | 501 |
|  |  | SVDSS | 15406 | 9943 | 5463 |
| LRA | 30X | Sniffles | 18875 | 8671 | 9925 |
|  |  | CuteSV | 13732 | 5985 | 7643 |
|  |  | SVIM | 18315 | 8380 | 9907 |
|  |  | PBSV | 12208 | 5747 | 6447 |
|  |  | SVDSS | 57893 | 35404 | 22489 |
|  | 20X | Sniffles | 8006 | 3581 | 4316 |
|  |  | CuteSV | 7749 | 3404 | 4309 |
|  |  | SVIM | 7338 | 3481 | 3851 |
|  |  | PBSV | 7576 | 3569 | 4005 |
|  |  | SVDSS | 45597 | 28566 | 17031 |
|  | 10X | Sniffles | 1352 | 721 | 596 |
|  |  | CuteSV | 1170 | 598 | 571 |
|  |  | SVIM | 1052 | 571 | 477 |
|  |  | PBSV | 1249 | 666 | 583 |
|  |  | SVDSS | 23935 | 15431 | 8504 |
| NGMLR | 30X | Sniffles | 14552 | 7183 | 7206 |
|  |  | CuteSV | 16821 | 7916 | 8759 |
|  |  | SVIM | 15399 | 8000 | 7251 |
|  |  | PBSV | 9931 | 4977 | 4940 |
|  |  | SVDSS | 49919 | 33107 | 16812 |
|  | 20X | Sniffles | 5903 | 2966 | 2886 |
|  |  | CuteSV | 6879 | 3355 | 3472 |
|  |  | SVIM | 6076 | 3433 | 2592 |
|  |  | PBSV | 5850 | 3020 | 2827 |
|  |  | SVDSS | 38452 | 25997 | 12455 |
|  | 10X | Sniffles | 734 | 405 | 320 |
|  |  | CuteSV | 962 | 516 | 443 |
|  |  | SVIM | 844 | 533 | 305 |
|  |  | PBSV | 971 | 557 | 414 |
|  |  | SVDSS | 19215 | 13402 | 5813 |
| Pbmm2 | 30X | Sniffles | 17319 | 7689 | 9498 |
|  |  | CuteSV | 19190 | 8446 | 10627 |
|  |  | SVIM | 18176 | 8596 | 9505 |
|  |  | PBSV | 10439 | 4820 | 5589 |
|  |  | SVDSS | 49919 | 33107 | 16812 |
|  | 20X | Sniffles | 7328 | 3316 | 3976 |
|  |  | CuteSV | 7795 | 3503 | 4237 |
|  |  | SVIM | 7087 | 3565 | 3492 |
|  |  | PBSV | 6769 | 3195 | 3566 |
|  |  | SVDSS | 38452 | 25997 | 12455 |
|  | 10X | Sniffles | 1062 | 533 | 524 |
|  |  | CuteSV | 1124 | 574 | 548 |
|  |  | SVIM | 1018 | 568 | 445 |
|  |  | PBSV | 1162 | 608 | 554 |
|  |  | SVDSS | 19215 | 13402 | 5813 |

**Table S2.** Summary for the SV count in terms of deletions and insertions for the NA24385 sample with different SV callers following the alignment with the four evaluated aligners at different depths of coverages**.**

| Aligner | Coverage | SV caller | Total | DEL | INS |
| --- | --- | --- | --- | --- | --- |
| Minimap2 | total | Sniffles | 22524 | 10222 | 12302 |
|  |  | CuteSV | 21182 | 9173 | 11873 |
|  |  | SVIM | 44508 | 26494 | 17987 |
|  |  | PBSV | 19572 | 8320 | 11252 |
|  |  | SVDSS | 58345 | 35686 | 22659 |
|  | 30X | Sniffles | 18330 | 8430 | 9756 |
|  |  | CuteSV | 19608 | 8517 | 10974 |
|  |  | SVIM | 33690 | 20999 | 12668 |
|  |  | PBSV | 17976 | 7680 | 10296 |
|  |  | SVDSS | 44195 | 27436 | 16759 |
|  | 20X | Sniffles | 13088 | 6043 | 6958 |
|  |  | CuteSV | 14292 | 6223 | 7994 |
|  |  | SVIM | 21945 | 14537 | 7391 |
|  |  | PBSV | 14572 | 6320 | 8252 |
|  |  | SDVSS | 41901 | 28374 | 13527 |
|  | 10X | Sniffles | 4058 | 1923 | 2100 |
|  |  | CuteSV | 2580 | 1334 | 1230 |
|  |  | SVIM | 5701 | 4120 | 1575 |
|  |  | PBSV | 6800 | 5735 | 1065 |
|  |  | SVDSS | 32374 | 20318 | 12056 |
| LRA | total | Sniffles | 21660 | 9695 | 11697 |
|  |  | CuteSV | 21222 | 9173 | 11913 |
|  |  | SVIM | 21592 | 9507 | 12052 |
|  |  | PBSV | 20695 | 9733 | 9210 |
|  |  | SVDSS | 48534 | 28225 | 20309 |
|  | 30X | Sniffles | 19050 | 8520 | 10313 |
|  |  | CuteSV | 19535 | 8477 | 10941 |
|  |  | SVIM | 19043 | 8647 | 10371 |
|  |  | PBSV | 18836 | 8721 | 8613 |
|  |  | SVDSS | 19394 | 12370 | 7024 |
|  | 20X | Sniffles | 13774 | 6140 | 7509 |
|  |  | CuteSV | 14234 | 6183 | 7976 |
|  |  | SVIM | 13493 | 6290 | 7192 |
|  |  | PBSV | 14145 | 6540 | 6535 |
|  |  | SVDSS | 19505 | 12270 | 7235 |
|  | 10X | Sniffles | 4455 | 2013 | 2418 |
|  |  | CuteSV | 4573 | 1975 | 2574 |
|  |  | SVIM | 4242 | 2046 | 2192 |
|  |  | PBSV | 4750 | 2238 | 2243 |
|  |  | SVDSS | 31196 | 19772 | 11424 |
| NGMLR | total | Sniffles | 18215 | 8299 | 9730 |
|  |  | CuteSV | 20638 | 8738 | 10049 |
|  |  | SVIM | 18502 | 9133 | 9223 |
|  |  | PBSV | 19577 | 8137 | 7516 |
|  |  | SVDSS | 62482 | 40709 | 21773 |
|  | 30X | Sniffles | 15483 | 7240 | 8122 |
|  |  | CuteSV | 17095 | 8068 | 8896 |
|  |  | SVIM | 15625 | 8176 | 7337 |
|  |  | PBSV | 17345 | 7630 | 6705 |
|  |  | SVDSS | 37876 | 25129 | 12747 |
|  | 20X | Sniffles | 10655 | 5017 | 5559 |
|  |  | CuteSV | 11944 | 5697 | 6157 |
|  |  | SVIM | 10537 | 5792 | 4668 |
|  |  | PBSV | 12218 | 5676 | 4864 |
|  |  | SVDSS | 42354 | 28580 | 13774 |
|  | 10X | Sniffles | 3006 | 1452 | 1524 |
|  |  | CuteSV | 3296 | 1649 | 1620 |
|  |  | SVIM | 2860 | 1717 | 1123 |
|  |  | PBSV | 3433 | 1739 | 1379 |
|  |  | SVDSS | 22160 | 15344 | 6816 |
| Pbmm2 | total | Sniffles | 21198 | 9156 | 11887 |
|  |  | CuteSV | 20649 | 8738 | 10049 |
|  |  | SVIM | 21942 | 10042 | 11808 |
|  |  | PBSV | 20645 | 8928 | 9718 |
|  |  | SVDSS | 24017 | 8320 | 15697 |
|  | 30X | Sniffles | 17736 | 7769 | 9855 |
|  |  | CuteSV | 19209 | 8483 | 10585 |
|  |  | SVIM | 17805 | 8572 | 9169 |
|  |  | PBSV | 18183 | 8053 | 8519 |
|  |  | SVDSS | 30740 | 19683 | 11057 |
|  | 20X | Sniffles | 13037 | 5686 | 7285 |
|  |  | CuteSV | 14126 | 6174 | 7856 |
|  |  | SVIM | 12802 | 6298 | 6453 |
|  |  | PBSV | 13709 | 6138 | 6436 |
|  |  | SVDSS | 21532 | 13849 | 7683 |
|  | 10X | Sniffles | 3776 | 1673 | 2082 |
|  |  | CuteSV | 4078 | 1810 | 2231 |
|  |  | SVIM | 3606 | 1875 | 1716 |
|  |  | PBSV | 4102 | 1936 | 1891 |
|  |  | SVDSS | 8500 | 5426 | 3074 |

**Table S3.** Summary for the SV count in terms of deletions and insertions for the SI00001 sample with different SV callers following the alignment with the four evaluated aligners at different depths of coverage.

| Aligner | Coverage | SV caller | Total | DEL | INS |
| --- | --- | --- | --- | --- | --- |
| Minimap2 | total | Sniffles | 9756 | 5033 | 4723 |
|  |  | CuteSV | 8971 | 4478 | 4493 |
|  |  | SVIM | 9164 | 4871 | 4293 |
|  |  | PBSV | 4604 | 2547 | 2057 |
|  |  | SVDSS | 6909 | 3571 | 3338 |
|  | 30X | Sniffles | 7092 | 3941 | 3151 |
|  |  | CuteSV | 7266 | 4022 | 3244 |
|  |  | SVIM | 6764 | 3748 | 3016 |
|  |  | PBSV | 2932 | 1647 | 1285 |
|  |  | SVDSS | 4223 | 2162 | 2061 |
|  | 20X | Sniffles | 3240 | 1931 | 1309 |
|  |  | CuteSV | 3480 | 2035 | 1445 |
|  |  | SVIM | 3063 | 1787 | 1276 |
|  |  | PBSV | 1080 | 601 | 479 |
|  |  | SVDSS | 2494 | 1266 | 1228 |
|  | 10X | Sniffles | 292 | 181 | 111 |
|  |  | CuteSV | 328 | 190 | 138 |
|  |  | SVIM | 242 | 155 | 87 |
|  |  | PBSV | 110 | 45 | 65 |
|  |  | SVDSS | 868 | 438 | 430 |
| LRA | total | Sniffles | 10122 | 5503 | 4619 |
|  |  | CuteSV | 9215 | 4924 | 4291 |
|  |  | SVIM | 9280 | 4929 | 4351 |
|  |  | PBSV | 8550 | 4791 | 3759 |
|  |  | SVDSS | 11011 | 5822 | 5189 |
|  | 30X | Sniffles | 7633 | 4288 | 3345 |
|  |  | CuteSV | 7043 | 3900 | 3143 |
|  |  | SVIM | 7044 | 3908 | 3136 |
|  |  | PBSV | 6789 | 3854 | 2935 |
|  |  | SVDSS | 7595 | 3963 | 3632 |
|  | 20X | Sniffles | 2677 | 1573 | 1104 |
|  |  | CuteSV | 3244 | 1838 | 1406 |
|  |  | SVIM | 3252 | 1862 | 1390 |
|  |  | PBSV | 3261 | 1874 | 1387 |
|  |  | SVDSS | 5228 | 2728 | 2500 |
|  | 10X | Sniffles | 305 | 203 | 102 |
|  |  | CuteSV | 362 | 181 | 181 |
|  |  | SVIM | 255 | 156 | 99 |
|  |  | PBSV | 294 | 190 | 104 |
|  |  | SVDSS | 2184 | 1136 | 1048 |
| NGMLR | total | Sniffles | 9256 | 4810 | 4446 |
|  |  | CuteSV | 8972 | 4770 | 4202 |
|  |  | SVIM | 8527 | 4586 | 3941 |
|  |  | PBSV | 8431 | 4597 | 3834 |
|  |  | SVDSS | 7480 | 4169 | 3311 |
|  | 30X | Sniffles | 7207 | 3945 | 3262 |
|  |  | CuteSV | 7104 | 3943 | 3161 |
|  |  | SVIM | 6286 | 3494 | 2792 |
|  |  | PBSV | 6587 | 3734 | 2853 |
|  |  | SVDSS | 4519 | 2555 | 1964 |
|  | 20X | Sniffles | 3591 | 2053 | 1538 |
|  |  | CuteSV | 3548 | 2046 | 1502 |
|  |  | SVIM | 2806 | 1591 | 1215 |
|  |  | PBSV | 2438 | 1418 | 1020 |
|  |  | SVDSS | 2835 | 1625 | 1210 |
|  | 10X | Sniffles | 163 | 100 | 63 |
|  |  | CuteSV | 181 | 107 | 74 |
|  |  | SVIM | 132 | 88 | 44 |
|  |  | PBSV | 159 | 88 | 71 |
|  |  | SVDSS | 1122 | 648 | 474 |
| Pbmm2 | total | Sniffles | 2470 | 1347 | 1123 |
|  |  | CuteSV | 3973 | 2167 | 1806 |
|  |  | SVIM | 3806 | 2106 | 1700 |
|  |  | PBSV | 3102 | 1867 | 1235 |
|  |  | SVDSS | 1895 | 954 | 941 |
|  | 30X | Sniffles | 1541 | 851 | 690 |
|  |  | CuteSV | 2294 | 1352 | 942 |
|  |  | SVIM | 2047 | 1185 | 862 |
|  |  | PBSV | 2327 | 1383 | 944 |
|  |  | SVDSS | 1020 | 531 | 489 |
|  | 20X | Sniffles | 468 | 264 | 204 |
|  |  | CuteSV | 706 | 436 | 270 |
|  |  | SVIM | 571 | 336 | 235 |
|  |  | PBSV | 722 | 450 | 272 |
|  |  | SVDSS | 527 | 277 | 250 |
|  | 10X | Sniffles | 35 | 17 | 18 |
|  |  | CuteSV | 53 | 31 | 22 |
|  |  | SVIM | 39 | 21 | 18 |
|  |  | PBSV | 59 | 36 | 23 |
|  |  | SVDSS | 172 | 85 | 87 |

**Table S4.** The performance of “npInv” SV caller in detection of inversions for the NA12878 sample following the alignment of the four aligners under evaluation Minimap2, LRA, ngmlr and pbmm2 at various degrees of coverage

| Aligner | Coverage | SV caller | INV |
| --- | --- | --- | --- |
| Minimap2 | 30X | NpInv | 19 |
|  | 20X | NpInv | 6 |
|  | 10X | NpInv | 0 |
| LRA | 30X | NpInv | 35 |
|  | 20X | NpInv | 6 |
|  | 10X | NpInv | 0 |
| ngmlr | 30X | NpInv | 27 |
|  | 20X | NpInv | 5 |
|  | 10X | NpInv | 0 |
| pbmm2 | 30X | NpInv | 20 |
|  | 20X | NpInv | 6 |
|  | 10X | NpInv | 0 |

**Table S5.** The performance of “NpInv” SV caller in detection of inversions for the NA24385 sample following the alignment of the four aligners under evaluation Minimap2, LRA, NGMLR and pbmm2 at various degrees of coverages

| Aligner | Coverage | SV caller | INV |
| --- | --- | --- | --- |
| Minimap2 | total | NpInv | 43 |
|  | 30X | NpInv | 29 |
|  | 20X | NpInv | 12 |
|  | 10X | NpInv | 4 |
| LRA | total | NpInv | 53 |
|  | 30X | NpInv | 35 |
|  | 20X | NpInv | 16 |
|  | 10X | NpInv | 2 |
| ngmlr | total | NpInv | 44 |
|  | 30X | NpInv | 31 |
|  | 20X | NpInv | 15 |
|  | 10X | NpInv | 1 |
| pbmm2 | total | NpInv | 37 |
|  | 30X | NpInv | 24 |
|  | 20X | NpInv | 14 |
|  | 10X | NpInv | 2 |

**Table S6.** The performance of “npInv” SV caller in detection of inversions for the NA12878 sample following the alignment of the four aligners under evaluation Minimap2, LRA, ngmlr and pbmm2 at various degrees of coverages

| Aligner | Coverage | SV caller | INV |
| --- | --- | --- | --- |
| Minimap2 | total | NpInv | 185 |
|  | 30X | NpInv | 76 |
|  | 20X | NpInv | 15 |
|  | 10X | NpInv | 3 |
| LRA | total | NpInv | 201 |
|  | 30X | NpInv | 82 |
|  | 20X | NpInv | 23 |
|  | 10X | NpInv | 3 |
| ngmlr | total | NpInv | 218 |
|  | 30X | NpInv | 93 |
|  | 20X | NpInv | 18 |
|  | 10X | NpInv | 1 |
| pbmm2 | total | NpInv | 85 |
|  | 30X | NpInv | 24 |
|  | 20X | NpInv | 6 |
|  | 10X | NpInv | 0 |

**Table. S7** The distribution of SV count in different length groups for different aligners (Minimap2, LRA, Ngmlr and Pbmm2) with different SV callers (CuteSV, Sniffles, SVIM, PBSV and SVDSS) across various sequencing coverages for sample NA12878

| Aligner | Coverage | SV caller | Total No. | <50 | 50-250 | 251-500 | 501-750 | 751-1000 | 1000-5000 | >5000 |
| --- | --- | --- | --- | --- | --- | --- | --- | --- | --- | --- |
| Minimap2 | Total | Sniffles | 17532 | 32 | 8144 | 3247 | 620 | 305 | 745 | 219 |
|  |  | CuteSV | 14266 | 0 | 11776 | 4496 | 891 | 433 | 1345 | 359 |
|  |  | SVIM | 17699 | 0 | 7916 | 3209 | 549 | 260 | 534 | 109 |
|  |  | PBSV | 10251 | 33 | 5999 | 2073 | 392 | 195 | 554 | 98 |
|  |  | SVDSS | 47176 | 29056 | 11595 | 4018 | 751 | 434 | 1127 | 195 |
|  | 20X | Sniffles | 7761 | 14 | 4706 | 2056 | 396 | 157 | 376 | 122 |
|  |  | CuteSV | 8224 | 0 | 5021 | 2149 | 431 | 153 | 471 | 141 |
|  |  | SVIM | 7006 | 0 | 4409 | 1901 | 310 | 113 | 257 | 52 |
|  |  | PBSV | 6829 | 27 | 4456 | 1559 | 272 | 129 | 341 | 63 |
|  |  | SVDSS | 33896 | 21167 | 8121 | 2871 | 525 | 303 | 776 | 133 |
|  | 10X | Sniffles | 1261 | 15 | 763 | 378 | 47 | 8 | 54 | 27 |
|  |  | CuteSV | 1260 | 0 | 777 | 364 | 49 | 9 | 76 | 27 |
|  |  | SVIM | 1036 | 0 | 654 | 321 | 28 | 3 | 35 | 5 |
|  |  | PBSV | 1130 | 1 | 765 | 267 | 46 | 10 | 38 | 8 |
|  |  | SVDSS | 15406 | 9616 | 3592 | 1391 | 236 | 131 | 373 | 67 |
| LRA | Total | Sniffles | 18875 | 5 | 7059 | 3429 | 862 | 479 | 1412 | 574 |
|  |  | CuteSV | 13732 | 0 | 10963 | 4346 | 965 | 567 | 1563 | 330 |
|  |  | SVIM | 18315 | 0 | 7458 | 3300 | 731 | 412 | 1057 | 173 |
|  |  | PBSV | 12208 | 28 | 6290 | 2564 | 571 | 335 | 828 | 173 |
|  |  | SVDSS | 57893 | 33351 | 15164 | 5405 | 1170 | 658 | 1796 | 349 |
|  | 20X | Sniffles | 8006 | 1 | 3987 | 2126 | 539 | 296 | 752 | 337 |
|  |  | CuteSV | 7749 | 0 | 8094 | 3385 | 806 | 451 | 1303 | 253 |
|  |  | SVIM | 7338 | 0 | 4059 | 1972 | 432 | 254 | 524 | 95 |
|  |  | PBSV | 7576 | 22 | 4412 | 1861 | 408 | 242 | 515 | 114 |
|  |  | SVDSS | 45597 | 26560 | 11740 | 4283 | 883 | 485 | 1375 | 271 |
|  | 10X | Sniffles | 1352 | 0 | 566 | 345 | 109 | 46 | 194 | 104 |
|  |  | CuteSV | 1170 | 0 | 628 | 342 | 78 | 23 | 82 | 17 |
|  |  | SVIM | 1052 | 0 | 569 | 322 | 52 | 23 | 64 | 20 |
|  |  | PBSV | 1249 | 8 | 729 | 317 | 64 | 25 | 78 | 28 |
|  |  | SVDSS | 23935 | 14039 | 5954 | 2335 | 441 | 281 | 717 | 168 |
| NGMLR | Total | Sniffles | 14552 | 29 | 6490 | 2890 | 624 | 331 | 595 | 241 |
|  |  | CuteSV | 16821 | 0 | 7919 | 3125 | 668 | 325 | 812 | 204 |
|  |  | SVIM | 15399 | 0 | 7416 | 2450 | 562 | 280 | 480 | 123 |
|  |  | PBSV | 9931 | 204 | 5530 | 1565 | 469 | 284 | 759 | 136 |
|  |  | SVDSS | 49919 | 34721 | 9102 | 4649 | 468 | 297 | 638 | 1 |
|  | 20X | Sniffles | 5903 | 17 | 8826 | 1643 | 334 | 160 | 281 | 131 |
|  |  | CuteSV | 6879 | 0 | 4439 | 1790 | 352 | 167 | 346 | 103 |
|  |  | SVIM | 6076 | 0 | 4128 | 1397 | 289 | 142 | 206 | 68 |
|  |  | PBSV | 5850 | 64 | 4076 | 1120 | 278 | 160 | 387 | 50 |
|  |  | SVDSS | 38452 | 25291 | 9540 | 2468 | 450 | 274 | 429 | 0 |
|  | 10X | Sniffles | 734 | 4 | 414 | 227 | 27 | 9 | 49 | 24 |
|  |  | CuteSV | 962 | 0 | 598 | 277 | 34 | 8 | 49 | 14 |
|  |  | SVIM | 844 | 0 | 572 | 214 | 22 | 9 | 27 | 4 |
|  |  | PBSV | 971 | 4 | 709 | 187 | 26 | 13 | 41 | 1 |
|  |  | SVDSS | 19215 | 12785 | 4677 | 1224 | 220 | 132 | 177 | 0 |
| Pbmm2 | total | Sniffles | 17319 | 22 | 7884 | 3240 | 687 | 322 | 614 | 169 |
|  |  | CuteSV | 19190 | 0 | 8826 | 3335 | 644 | 308 | 750 | 218 |
|  |  | SVIM | 18176 | 0 | 8066 | 3111 | 599 | 294 | 551 | 157 |
|  |  | PBSV | 10439 | 56 | 5886 | 2110 | 368 | 159 | 450 | 155 |
|  |  | SVDSS | 49919 | 31669 | 11462 | 4006 | 713 | 370 | 833 | 104 |
|  | 20X | Sniffles | 7328 | 5 | 4568 | 1987 | 380 | 135 | 209 | 58 |
|  |  | CuteSV | 7795 | 27 | 4502 | 1589 | 232 | 99 | 242 | 85 |
|  |  | SVIM | 7087 | 0 | 4515 | 1850 | 302 | 116 | 230 | 89 |
|  |  | PBSV | 6769 | 27 | 4502 | 1589 | 232 | 99 | 242 | 85 |
|  |  | SVDSS | 38452 | 16473 | 5705 | 2097 | 335 | 155 | 330 | 43 |
|  | 10X | Sniffles | 1062 | 2 | 659 | 304 | 55 | 7 | 25 | 13 |
|  |  | CuteSV | 1124 | 0 | 731 | 312 | 30 | 2 | 36 | 21 |
|  |  | SVIM | 1018 | 0 | 660 | 292 | 26 | 2 | 31 | 11 |
|  |  | PBSV | 1162 | 7 | 805 | 276 | 37 | 5 | 31 | 5 |
|  |  | SVDSS | 19215 | 6797 | 2362 | 920 | 130 | 63 | 124 | 13 |

**Table. S8** The distribution of SV count in different length groups for different aligners (Minimap2, LRA, Ngmlr and Pbmm2) with different SV callers (CuteSV, Sniffles, SVIM, PBSV and SVDSS) across various sequencing coverages for sample NA24385

| Aligner | Coverage | SV caller | Total No. | <50 | 50-250 | 251-500 | 501-750 | 751-1000 | 1000-5000 | >5000 |
| --- | --- | --- | --- | --- | --- | --- | --- | --- | --- | --- |
| inimap2 | total | Sniffles | 22524 | 530 | 14341 | 4814 | 1013 | 511 | 1637 | 742 |
|  |  | CuteSV | 21182 | 0 | 2120 | 821 | 160 | 99 | 301 | 180 |
|  |  | SVIM | 44508 | 22374 | 14752 | 4611 | 878 | 449 | 1296 | 310 |
|  |  | PBSV | 19572 | 39 | 6564 | 2324 | 441 | 220 | 632 | 258 |
|  |  | SVDSS | 58345 | 35837 | 14588 | 4846 | 1014 | 509 | 1322 | 229 |
|  | 30X | Sniffles | 18330 | 386 | 11396 | 4154 | 791 | 417 | 1246 | 510 |
|  |  | CuteSV | 19608 | 0 | 1798 | 736 | 130 | 77 | 251 | 158 |
|  |  | SVIM | 33690 | 16571 | 11207 | 3772 | 671 | 351 | 986 | 230 |
|  |  | PBSV | 17976 | 204 | 5530 | 1565 | 469 | 284 | 759 | 136 |
|  |  | SVDSS | 44195 | 3043 | 10588 | 3708 | 711 | 380 | 1005 | 168 |
|  | 20X | Sniffles | 13088 | 147 | 7962 | 3191 | 618 | 273 | 837 | 334 |
|  |  | CuteSV | 14292 | 0 | 1268 | 563 | 104 | 54 | 181 | 143 |
|  |  | SVIM | 21945 | 11105 | 6945 | 2544 | 453 | 200 | 614 | 139 |
|  |  | PBSV | 21945 | 27 | 4456 | 1559 | 272 | 129 | 341 | 63 |
|  |  | SVDSS | 41901 | 20373 | 7573 | 2772 | 507 | 270 | 761 | 118 |
|  | 10X | Sniffles | 4058 | 90 | 2363 | 1083 | 240 | 91 | 225 | 98 |
|  |  | CuteSV | 2580 | 0 | 331 | 174 | 22 | 15 | 39 | 80 |
|  |  | SVIM | 5701 | 3043 | 1692 | 690 | 114 | 40 | 111 | 28 |
|  |  | PBSV | 6800 | 1 | 765 | 267 | 46 | 10 | 38 | 8 |
|  |  | SVDSS | 32374 | 9133 | 3279 | 1297 | 229 | 127 | 353 | 49 |
| LRA | total | Sniffles | 21660 | 20 | 11430 | 4598 | 1218 | 668 | 2517 | 1229 |
|  |  | CuteSV | 21222 | 0 | 12253 | 4672 | 1114 | 648 | 2048 | 489 |
|  |  | SVIM | 21592 | 3 | 12488 | 4770 | 1174 | 699 | 2043 | 405 |
|  |  | PBSV | 20695 | 55 | 11445 | 4336 | 1040 | 668 | 2576 | 398 |
|  |  | SVDSS | 48534 | 29811 | 12136 | 4031 | 843 | 423 | 1099 | 190 |
|  | 30X | Sniffles | 19050 | 12 | 9947 | 4283 | 1077 | 637 | 2178 | 928 |
|  |  | CuteSV | 19535 | 0 | 11323 | 4404 | 1029 | 618 | 1824 | 411 |
|  |  | SVIM | 19043 | 4 | 10866 | 4374 | 1031 | 622 | 1798 | 341 |
|  |  | PBSV | 18836 | 55 | 10628 | 4118 | 949 | 596 | 2032 | 364 |
|  |  | SVDSS | 19394 | 10827 | 4654 | 1673 | 324 | 206 | 579 | 131 |
|  | 20X | Sniffles | 13774 | 6 | 6960 | 3301 | 870 | 514 | 1562 | 567 |
|  |  | CuteSV | 14234 | 0 | 8094 | 3385 | 806 | 451 | 1303 | 253 |
|  |  | SVIM | 13493 | 0 | 7503 | 3275 | 764 | 466 | 1279 | 206 |
|  |  | PBSV | 14145 | 34 | 8100 | 3202 | 760 | 443 | 1318 | 249 |
|  |  | SVDSS | 19505 | 0 | 32 | 4 | 57 | 190 | 213 | 31 |
|  | 10X | Sniffles | 4455 | 0 | 2017 | 1173 | 360 | 189 | 565 | 173 |
|  |  | CuteSV | 4573 | 0 | 2426 | 1211 | 307 | 156 | 436 | 68 |
|  |  | SVIM | 4242 | 0 | 2191 | 1137 | 288 | 171 | 399 | 57 |
|  |  | PBSV | 4750 | 8 | 2651 | 1146 | 284 | 161 | 420 | 72 |
|  |  | SVDSS | 31196 | 18099 | 7703 | 2936 | 664 | 404 | 1151 | 239 |
| NGMLR | total | Sniffles | 18215 | 1030 | 10795 | 4109 | 1007 | 545 | 1284 | 504 |
|  |  | CuteSV | 20638 | 0 | 12383 | 4728 | 1151 | 568 | 1610 | 387 |
|  |  | SVIM | 18502 | 8 | 12435 | 4553 | 1088 | 547 | 1215 | 201 |
|  |  | PBSV | 19577 | 683 | 10985 | 3803 | 1118 | 623 | 1745 | 351 |
|  |  | SVDSS | 62482 | 39691 | 15161 | 4293 | 884 | 518 | 1130 | 1 |
|  | 30X | Sniffles | 15483 | 773 | 9069 | 3702 | 842 | 470 | 1063 | 360 |
|  |  | CuteSV | 17095 | 0 | 11132 | 4240 | 1006 | 497 | 1354 | 306 |
|  |  | SVIM | 15625 | 4 | 10556 | 3676 | 889 | 484 | 925 | 151 |
|  |  | PBSV | 17345 | 437 | 10146 | 3262 | 964 | 556 | 1508 | 272 |
|  |  | SVDSS | 37876 | 24721 | 9102 | 2649 | 468 | 297 | 638 | 1 |
|  | 20X | Sniffles | 10655 | 525 | 6104 | 2686 | 637 | 328 | 685 | 229 |
|  |  | CuteSV | 11944 | 0 | 7666 | 3005 | 709 | 360 | 879 | 186 |
|  |  | SVIM | 10537 | 0 | 7074 | 2406 | 600 | 314 | 593 | 80 |
|  |  | PBSV | 12218 | 179 | 7520 | 2222 | 656 | 399 | 1033 | 155 |
|  |  | SVDSS | 42354 | 27536 | 10008 | 2764 | 553 | 312 | 727 | 1 |
|  | 10X | Sniffles | 3006 | 292 | 1667 | 828 | 184 | 100 | 175 | 52 |
|  |  | CuteSV | 3296 | 0 | 2020 | 884 | 178 | 96 | 205 | 37 |
|  |  | SVIM | 2860 | 0 | 1866 | 672 | 151 | 83 | 132 | 19 |
|  |  | PBSV | 2860 | 22 | 2218 | 638 | 173 | 110 | 247 | 19 |
|  |  | SVDSS | 22160 | 14548 | 5178 | 1491 | 300 | 164 | 365 | 0 |
| Pbmm2 | total | Sniffles | 21198 | 21 | 12789 | 4626 | 1068 | 582 | 1696 | 437 |
|  |  | CuteSV | 20649 | 0 | 8094 | 3385 | 806 | 451 | 1303 | 253 |
|  |  | SVIM | 21942 | 8 | 13641 | 4730 | 1069 | 620 | 1579 | 346 |
|  |  | PBSV | 20645 | 60 | 12314 | 4389 | 1008 | 583 | 1673 | 375 |
|  |  | SVDSS | 24017 | 27470 | 9459 | 3407 | 657 | 366 | 1085 | 179 |
|  | 30X | Sniffles | 17736 | 17 | 10569 | 4111 | 880 | 498 | 1363 | 316 |
|  |  | CuteSV | 19209 | 0 | 11798 | 4221 | 945 | 538 | 1429 | 365 |
|  |  | SVIM | 17805 | 2 | 10979 | 4012 | 830 | 515 | 1243 | 254 |
|  |  | PBSV | 18183 | 54 | 11061 | 3898 | 854 | 494 | 1359 | 306 |
|  |  | SVDSS | 30740 | 19694 | 6683 | 2483 | 452 | 255 | 758 | 123 |
|  | 20X | Sniffles | 13037 | 8 | 7654 | 3153 | 731 | 361 | 935 | 203 |
|  |  | CuteSV | 14126 | 0 | 8542 | 3225 | 742 | 387 | 1034 | 258 |
|  |  | SVIM | 12802 | 0 | 7776 | 2996 | 647 | 361 | 867 | 175 |
|  |  | PBSV | 13709 | 35 | 8385 | 3001 | 646 | 354 | 972 | 225 |
|  |  | SVDSS | 21532 | 13802 | 4621 | 1781 | 328 | 173 | 536 | 91 |
|  | 10X | Sniffles | 3776 | 3 | 2160 | 1030 | 240 | 100 | 201 | 45 |
|  |  | CuteSV | 4078 | 0 | 2429 | 1031 | 224 | 105 | 255 | 58 |
|  |  | SVIM | 3606 | 0 | 2141 | 955 | 188 | 100 | 196 | 39 |
|  |  | PBSV | 4102 | 14 | 2586 | 921 | 194 | 96 | 229 | 46 |
|  |  | SVDSS | 8500 | 5366 | 1845 | 746 | 140 | 70 | 214 | 39 |

**Table. S9** The distribution of SV count in different length groups for different aligners (Minimap2, LRA, Ngmlr and Pbmm2) with different SV callers (CuteSV, Sniffles, SVIM, PBSV and SVDSS) across various sequencing coverages for sample SI00001

| Aligner | Coverage | SV caller | Total No. | <50 | 50-250 | 251-500 | 501-750 | 751-1000 | 1000-5000 | >5000 |
| --- | --- | --- | --- | --- | --- | --- | --- | --- | --- | --- |
| Minimap2 | Total | Sniffles | 9756 | 115 | 406 | 205 | 977 | 3137 | 3658 | 1673 |
|  |  | CuteSV | 8971 | 0 | 425 | 208 | 1002 | 3193 | 3678 | 1636 |
|  |  | SVIM | 9164 | 1 | 389 | 192 | 826 | 2873 | 3707 | 1365 |
|  |  | PBSV | 4604 | 1 | 170 | 84 | 456 | 1419 | 1874 | 696 |
|  |  | SVDSS | 6909 | 525 | 426 | 136 | 587 | 1915 | 2541 | 779 |
|  | 30X | Sniffles | 7092 | 48 | 298 | 171 | 731 | 2350 | 2702 | 1086 |
|  |  | CuteSV | 7266 | 0 | 360 | 173 | 785 | 2496 | 2870 | 1089 |
|  |  | SVIM | 6764 | 0 | 310 | 161 | 637 | 2188 | 2805 | 806 |
|  |  | PBSV | 2932 | 0 | 131 | 67 | 320 | 895 | 1188 | 394 |
|  |  | SVDSS | 4223 | 0 | 279 | 98 | 408 | 1283 | 1682 | 473 |
|  | 20X | Sniffles | 3240 | 28 | 222 | 112 | 339 | 1019 | 1216 | 481 |
|  |  | CuteSV | 3480 | 0 | 291 | 134 | 392 | 1146 | 1337 | 492 |
|  |  | SVIM | 3063 | 0 | 240 | 114 | 294 | 955 | 1237 | 308 |
|  |  | PBSV | 1080 | 0 | 95 | 40 | 126 | 275 | 452 | 127 |
|  |  | SVDSS | 2494 | 0 | 157 | 65 | 233 | 769 | 1000 | 270 |
|  | 10X | Sniffles | 292 | 7 | 81 | 33 | 45 | 46 | 63 | 38 |
|  |  | CuteSV | 328 | 0 | 122 | 41 | 45 | 54 | 89 | 33 |
|  |  | SVIM | 242 | 0 | 98 | 35 | 17 | 45 | 64 | 13 |
|  |  | PBSV | 110 | 0 | 30 | 9 | 16 | 12 | 43 | 16 |
|  |  | SVDSS | 868 | 0 | 67 | 32 | 76 | 272 | 343 | 78 |
| LRA | total | Sniffles | 10122 | 4 | 359 | 225 | 982 | 3190 | 4058 | 1650 |
|  |  | CuteSV | 9215 | 0 | 334 | 172 | 939 | 3161 | 3701 | 1443 |
|  |  | SVIM | 9280 | 0 | 348 | 178 | 807 | 2897 | 3737 | 1315 |
|  |  | PBSV | 8550 | 1 | 315 | 166 | 804 | 2770 | 3505 | 1031 |
|  |  | SVDSS | 11011 | 10827 | 4654 | 1673 | 324 | 206 | 579 | 131 |
|  | 30X | Sniffles | 7633 | 1 | 265 | 173 | 791 | 2529 | 3108 | 1028 |
|  |  | CuteSV | 7043 | 0 | 274 | 148 | 753 | 2510 | 2844 | 928 |
|  |  | SVIM | 7044 | 0 | 269 | 135 | 635 | 2281 | 2901 | 824 |
|  |  | PBSV | 6789 | 2 | 254 | 132 | 644 | 2221 | 2787 | 781 |
|  |  | SVDSS | 7595 | 10827 | 4654 | 1673 | 324 | 206 | 579 | 131 |
|  | 20X | Sniffles | 2677 | 0 | 163 | 96 | 310 | 850 | 1071 | 291 |
|  |  | CuteSV | 3244 | 0 | 193 | 98 | 372 | 1144 | 1291 | 335 |
|  |  | SVIM | 3252 | 0 | 183 | 89 | 316 | 1037 | 1326 | 301 |
|  |  | PBSV | 3261 | 1 | 191 | 86 | 322 | 1058 | 1314 | 308 |
|  |  | SVDSS | 5228 | 0 | 32 | 4 | 57 | 190 | 213 | 31 |
|  | 10X | Sniffles | 305 | 1 | 54 | 35 | 50 | 36 | 117 | 24 |
|  |  | CuteSV | 362 | 0 | 77 | 40 | 46 | 50 | 81 | 14 |
|  |  | SVIM | 255 | 0 | 72 | 35 | 19 | 44 | 72 | 13 |
|  |  | PBSV | 294 | 0 | 82 | 35 | 27 | 46 | 82 | 27 |
|  |  | SVDSS | 2184 | 18099 | 7703 | 2936 | 664 | 404 | 1151 | 239 |
| NGMLR | total | Sniffles | 9256 | 43 | 205 | 126 | 891 | 3100 | 3641 | 1608 |
|  |  | CuteSV | 8972 | 0 | 241 | 146 | 934 | 3231 | 3704 | 1583 |
|  |  | SVIM | 8527 | 0 | 250 | 131 | 794 | 2873 | 3666 | 1129 |
|  |  | PBSV | 8431 | 0 | 190 | 99 | 746 | 2720 | 3474 | 1202 |
|  |  | SVDSS | 7480 | 1034 | 613 | 135 | 679 | 2249 | 2770 | 0 |
|  | 30X | Sniffles | 7207 | 31 | 162 | 111 | 707 | 2512 | 2905 | 1072 |
|  |  | CuteSV | 7104 | 0 | 210 | 126 | 748 | 2643 | 2991 | 1072 |
|  |  | SVIM | 6286 | 0 | 200 | 112 | 622 | 2232 | 2733 | 635 |
|  |  | PBSV | 6587 | 0 | 172 | 83 | 607 | 2183 | 2735 | 807 |
|  |  | SVDSS | 4519 | 24721 | 9102 | 2649 | 468 | 297 | 638 | 1 |
|  | 20X | Sniffles | 3591 | 13 | 123 | 75 | 359 | 1244 | 1447 | 513 |
|  |  | CuteSV | 3548 | 0 | 162 | 83 | 386 | 1309 | 1501 | 474 |
|  |  | SVIM | 2806 | 0 | 150 | 74 | 301 | 994 | 1218 | 207 |
|  |  | PBSV | 2438 | 0 | 131 | 43 | 237 | 808 | 1028 | 191 |
|  |  | SVDSS | 2835 | 27536 | 10008 | 2764 | 553 | 312 | 727 | 1 |
|  | 10X | Sniffles | 163 | 2 | 39 | 19 | 16 | 37 | 42 | 20 |
|  |  | CuteSV | 181 | 0 | 57 | 22 | 14 | 42 | 48 | 17 |
|  |  | SVIM | 132 | 0 | 53 | 17 | 9 | 27 | 30 | 5 |
|  |  | PBSV | 159 | 0 | 59 | 11 | 10 | 38 | 40 | 1 |
|  |  | SVDSS | 1122 | 14548 | 5178 | 1491 | 300 | 164 | 365 | 0 |
| Pbmm2 | total | Sniffles | 2470 | 7 | 104 | 60 | 331 | 926 | 1020 | 156 |
|  |  | CuteSV | 3973 | 0 | 118 | 77 | 426 | 1370 | 1543 | 730 |
|  |  | SVIM | 3806 | 0 | 124 | 73 | 356 | 1250 | 1536 | 514 |
|  |  | PBSV | 3102 | 0 | 201 | 80 | 436 | 1503 | 1883 | 970 |
|  |  | SVDSS | 1895 | 172 | 90 | 30 | 178 | 600 | 710 | 115 |
|  | 30X | Sniffles | 1541 | 5 | 84 | 45 | 227 | 604 | 603 | 68 |
|  |  | CuteSV | 2294 | 0 | 98 | 61 | 271 | 799 | 821 | 451 |
|  |  | SVIM | 2047 | 0 | 94 | 61 | 227 | 690 | 780 | 230 |
|  |  | PBSV | 2327 | 0 | 95 | 62 | 259 | 742 | 857 | 450 |
|  |  | SVDSS | 1020 | 19694 | 6683 | 2483 | 452 | 255 | 758 | 123 |
|  | 20X | Sniffles | 468 | 2 | 57 | 22 | 77 | 178 | 155 | 15 |
|  |  | CuteSV | 706 | 0 | 74 | 23 | 87 | 238 | 213 | 171 |
|  |  | SVIM | 571 | 0 | 63 | 24 | 73 | 193 | 173 | 60 |
|  |  | PBSV | 722 | 0 | 67 | 22 | 95 | 216 | 211 | 180 |
|  |  | SVDSS | 527 | 13802 | 4621 | 1781 | 328 | 173 | 536 | 91 |
|  | 10X | Sniffles | 35 | 0 | 21 | 5 | 2 | 2 | 7 | 1 |
|  |  | CuteSV | 53 | 0 | 26 | 4 | 7 | 3 | 13 | 9 |
|  |  | SVIM | 39 | 0 | 23 | 5 | 4 | 2 | 5 | 2 |
|  |  | PBSV | 59 | 0 | 22 | 4 | 15 | 3 | 10 | 10 |
|  |  | SVDSS | 172 | 5366 | 1845 | 746 | 140 | 70 | 214 | 36 |

**Table S10** The precision, recall, and F-score values for SV calling for the NA24385 sample with Sniffles, SVIM, CuteSV, PBSV and SVDSS following Alignment with Minimap2 at different depths of coverage across the SV length groups

| Aligner | Coverage | SV caller | SV length groups | Precision | Recall | F1-score |
| --- | --- | --- | --- | --- | --- | --- |
| Minimap2 | total | Sniffles | 50-250 | 56.96% | 59.87% | 58.38% |
|  |  |  | 251-500 | 61.60% | 56.29% | 58.82% |
|  |  |  | 501-750 | 64.66% | 77.21% | 70.38% |
|  |  |  | 751-1000 | 70.45% | 76.35% | 73.28% |
|  |  |  | 1000-5000 | 72.80% | 62.97% | 67.53% |
|  |  |  | >5000 | 47.01% | 38.14% | 42.11% |
|  |  | Cutesv | 50-250 | 92.58% | 95.19% | 93.87% |
|  |  |  | 251-500 | 92.24% | 94.63% | 93.42% |
|  |  |  | 501-750 | 92.73% | 97.45% | 95.03% |
|  |  |  | 751-1000 | 93.42% | 96.29% | 94.83% |
|  |  |  | 1000-5000 | 94.40% | 94.73% | 94.56% |
|  |  |  | >5000 | 82.98% | 95.28% | 88.71% |
|  |  | SVIM | 50-250 | 56.19% | 65.91% | 60.66% |
|  |  |  | 251-500 | 61.25% | 67.27% | 64.12% |
|  |  |  | 501-750 | 68.31% | 81.09% | 74.16% |
|  |  |  | 751-1000 | 74.18% | 79.22% | 76.62% |
|  |  |  | 1000-5000 | 79.80% | 70.33% | 74.77% |
|  |  |  | >5000 | 83.02% | 70.49% | 76.24% |
|  |  | PBSV | 50-250 | 80.60% | 79.99% | 80.29% |
|  |  |  | 251-500 | 83.13% | 77.54% | 80.24% |
|  |  |  | 501-750 | 83.37% | 89.37% | 86.27% |
|  |  |  | 751-1000 | 85.40% | 91.83% | 88.50% |
|  |  |  | 1000-5000 | 89.96% | 85.97% | 87.92% |
|  |  |  | >5000 | 84.98% | 77.76% | 81.21% |
|  |  | SVDSS | 50-250 | 58.97% | 62.07% | 60.48% |
|  |  |  | 251-500 | 64.54% | 59.29% | 61.80% |
|  |  |  | 501-750 | 64.96% | 78.93% | 71.26% |
|  |  |  | 751-1000 | 70.88% | 77.94% | 74.24% |
|  |  |  | 1000-5000 | 79.91% | 71.06% | 75.23% |
|  |  |  | >5000 | 91.84% | 75.88% | 83.10% |
|  | 30X | Sniffles | 50-250 | 65.82% | 65.48% | 65.65% |
|  |  |  | 251-500 | 67.62% | 61.22% | 64.26% |
|  |  |  | 501-750 | 71.96% | 80.20% | 75.86% |
|  |  |  | 751-1000 | 76.10% | 79.00% | 77.52% |
|  |  |  | 1000-5000 | 81.12% | 69.28% | 74.74% |
|  |  |  | >5000 | 66.22% | 52.83% | 58.77% |
|  |  | Cutesv | 50-250 | 93.89% | 95.61% | 94.74% |
|  |  |  | 251-500 | 93.19% | 94.98% | 94.08% |
|  |  |  | 501-750 | 94.34% | 97.58% | 95.93% |
|  |  |  | 751-1000 | 95.29% | 96.61% | 95.95% |
|  |  |  | 1000-5000 | 95.39% | 95.43% | 95.41% |
|  |  |  | >5000 | 84.48% | 96.09% | 89.91% |
|  |  | SVIM | 50-250 | 65.97% | 69.86% | 67.86% |
|  |  |  | 251-500 | 68.77% | 70.18% | 69.47% |
|  |  |  | 501-750 | 75.96% | 82.88% | 79.27% |
|  |  |  | 751-1000 | 80.37% | 82.08% | 81.22% |
|  |  |  | 1000-5000 | 85.97% | 75.92% | 80.63% |
|  |  |  | >5000 | 88.91% | 77.76% | 82.96% |
|  |  | PBSV | 50-250 | 84.41% | 81.83% | 83.10% |
|  |  |  | 251-500 | 90.50% | 82.72% | 86.43% |
|  |  |  | 501-750 | 82.04% | 88.99% | 85.37% |
|  |  |  | 751-1000 | 82.05% | 88.23% | 85.03% |
|  |  |  | 1000-5000 | 86.73% | 85.10% | 85.91% |
|  |  |  | >5000 | 92.85% | 87.47% | 90.08% |
|  |  | SVDSS | 50-250 | 69.77% | 70.35% | 70.06% |
|  |  |  | 251-500 | 73.55% | 65.84% | 69.48% |
|  |  |  | 501-750 | 74.50% | 83.13% | 78.58% |
|  |  |  | 751-1000 | 77.46% | 82.71% | 80.00% |
|  |  |  | 1000-5000 | 85.13% | 76.75% | 80.73% |
|  |  |  | >5000 | 93.25% | 81.94% | 87.23% |
|  | 20X | Sniffles | 50-250 | 76.53% | 74.79% | 75.65% |
|  |  |  | 251-500 | 75.80% | 69.92% | 72.74% |
|  |  |  | 501-750 | 77.38% | 84.91% | 80.97% |
|  |  |  | 751-1000 | 84.63% | 85.26% | 84.94% |
|  |  |  | 1000-5000 | 88.70% | 78.64% | 83.37% |
|  |  |  | >5000 | 77.67% | 63.75% | 70.02% |
|  |  | Cutesv | 50-250 | 95.76% | 96.75% | 96.25% |
|  |  |  | 251-500 | 94.86% | 95.98% | 95.42% |
|  |  |  | 501-750 | 95.14% | 98.41% | 96.75% |
|  |  |  | 751-1000 | 96.64% | 97.67% | 97.15% |
|  |  |  | 1000-5000 | 96.49% | 97.00% | 96.74% |
|  |  |  | >5000 | 85.83% | 96.36% | 90.79% |
|  |  | SVIM | 50-250 | 79.20% | 79.19% | 79.20% |
|  |  |  | 251-500 | 79.75% | 77.99% | 78.86% |
|  |  |  | 501-750 | 83.20% | 88.61% | 85.82% |
|  |  |  | 751-1000 | 89.07% | 88.97% | 89.02% |
|  |  |  | 1000-5000 | 91.72% | 84.71% | 88.08% |
|  |  |  | >5000 | 93.60% | 86.79% | 90.07% |
|  |  | PBSV | 50-250 | 87.61% | 85.37% | 86.48% |
|  |  |  | 251-500 | 90.07% | 83.44% | 86.63% |
|  |  |  | 501-750 | 90.25% | 92.55% | 91.39% |
|  |  |  | 751-1000 | 92.48% | 93.96% | 93.21% |
|  |  |  | 1000-5000 | 95.79% | 91.34% | 93.51% |
|  |  |  | >5000 | 97.60% | 93.26% | 95.38% |
|  |  | SVDSS | 50-250 | 78.23% | 77.87% | 78.05% |
|  |  |  | 251-500 | 81.19% | 72.87% | 76.81% |
|  |  |  | 501-750 | 81.44% | 87.40% | 84.31% |
|  |  |  | 751-1000 | 83.67% | 87.49% | 85.54% |
|  |  |  | 1000-5000 | 89.51% | 81.64% | 85.40% |
|  |  |  | >5000 | 95.83% | 86.66% | 91.01% |
|  | 10X | Sniffles | 50-250 | 93.13% | 92.44% | 92.78% |
|  |  |  | 251-500 | 91.87% | 90.27% | 91.06% |
|  |  |  | 501-750 | 89.99% | 94.97% | 92.41% |
|  |  |  | 751-1000 | 95.11% | 94.91% | 95.01% |
|  |  |  | 1000-5000 | 97.05% | 94.24% | 95.63% |
|  |  |  | >5000 | 93.66% | 79.65% | 86.09% |
|  |  | Cutesv | 50-250 | 98.92% | 99.12% | 99.02% |
|  |  |  | 251-500 | 98.53% | 98.63% | 98.58% |
|  |  |  | 501-750 | 98.86% | 99.75% | 99.30% |
|  |  |  | 751-1000 | 99.47% | 98.94% | 99.20% |
|  |  |  | 1000-5000 | 99.23% | 99.37% | 99.30% |
|  |  |  | >5000 | 90.56% | 99.60% | 94.87% |
|  |  | SVIM | 50-250 | 95.08% | 94.79% | 94.93% |
|  |  |  | 251-500 | 95.03% | 93.55% | 94.28% |
|  |  |  | 501-750 | 95.10% | 97.64% | 96.36% |
|  |  |  | 751-1000 | 98.60% | 97.03% | 97.81% |
|  |  |  | 1000-5000 | 98.86% | 97.00% | 97.92% |
|  |  |  | >5000 | 97.72% | 98.25% | 97.98% |
|  |  | PBSV | 50-250 | 98.07% | 97.37% | 97.72% |
|  |  |  | 251-500 | 98.85% | 96.71% | 97.77% |
|  |  |  | 501-750 | 98.05% | 98.98% | 98.51% |
|  |  |  | 751-1000 | 99.26% | 99.68% | 99.47% |
|  |  |  | 1000-5000 | 99.65% | 98.88% | 99.26% |
|  |  |  | >5000 | 99.73% | 99.19% | 99.46% |
|  |  | SVDSS | 50-250 | 90.60% | 90.15% | 90.37% |
|  |  |  | 251-500 | 91.82% | 86.77% | 89.22% |
|  |  |  | 501-750 | 91.97% | 93.38% | 92.67% |
|  |  |  | 751-1000 | 91.93% | 94.17% | 93.03% |
|  |  |  | 1000-5000 | 95.20% | 91.34% | 93.23% |
|  |  |  | >5000 | 98.46% | 94.74% | 96.57% |

**Table S11** The precision, recall, and F-score values for SV calling for the NA24385 sample with Sniffles, SVIM, CuteSV, PBSV and SVDSS following Alignment with LRA at different depths of coverage across the SV length groups.

| Aligner | Coverage | SV caller | SV length groups | Precision | Recall | F1-score |
| --- | --- | --- | --- | --- | --- | --- |
| LRA | total | Sniffles | 50-250 | 89.25% | 42.66% | 57.73% |
|  |  |  | 251-500 | 93.95% | 29.17% | 44.51% |
|  |  |  | 501-750 | 79.66% | 39.90% | 53.17% |
|  |  |  | 751-1000 | 83.33% | 42.47% | 56.26% |
|  |  |  | 1000-5000 | 95.37% | 33.41% | 49.48% |
|  |  |  | >5000 | 98.14% | 43.29% | 60.08% |
|  |  | Cutesv | 50-250 | 62.30% | 69.92% | 65.89% |
|  |  |  | 251-500 | 60.56% | 67.69% | 63.93% |
|  |  |  | 501-750 | 60.85% | 79.76% | 69.04% |
|  |  |  | 751-1000 | 62.50% | 78.47% | 69.58% |
|  |  |  | 1000-5000 | 62.11% | 68.20% | 65.01% |
|  |  |  | >5000 | 68.91% | 64.82% | 66.81% |
|  |  | SVIM | 50-250 | 64.65% | 55.65% | 59.82% |
|  |  |  | 251-500 | 63.47% | 51.36% | 56.78% |
|  |  |  | 501-750 | 60.39% | 70.66% | 65.12% |
|  |  |  | 751-1000 | 60.53% | 69.78% | 64.83% |
|  |  |  | 1000-5000 | 63.99% | 57.56% | 60.60% |
|  |  |  | >5000 | 79.19% | 57.95% | 66.93% |
|  |  | PBSV | 50-250 | 67.39% | 61.13% | 64.11% |
|  |  |  | 251-500 | 67.15% | 56.32% | 61.26% |
|  |  |  | 501-750 | 63.91% | 75.62% | 69.27% |
|  |  |  | 751-1000 | 61.42% | 74.44% | 67.31% |
|  |  |  | 1000-5000 | 52.83% | 62.23% | 57.15% |
|  |  |  | >5000 | 78.28% | 60.24% | 68.09% |
|  |  | SVDSS | 50-250 | 58.97% | 62.07% | 60.48% |
|  |  |  | 251-500 | 64.54% | 59.29% | 61.80% |
|  |  |  | 501-750 | 64.96% | 78.93% | 71.26% |
|  |  |  | 751-1000 | 70.88% | 77.94% | 74.24% |
|  |  |  | 1000-5000 | 79.91% | 71.06% | 75.23% |
|  |  |  | >5000 | 91.84% | 75.88% | 83.10% |
|  | 30X | Sniffles | 50-250 | 92.75% | 53.16% | 67.58% |
|  |  |  | 251-500 | 95.61% | 31.43% | 47.30% |
|  |  |  | 501-750 | 83.39% | 43.46% | 57.14% |
|  |  |  | 751-1000 | 84.13% | 43.56% | 57.40% |
|  |  |  | 1000-5000 | 100.00% | 100.00% | 100.00% |
|  |  |  | >5000 | 98.42% | 51.23% | 67.39% |
|  |  | Cutesv | 50-250 | 64.99% | 71.34% | 68.01% |
|  |  |  | 251-500 | 62.74% | 68.58% | 65.53% |
|  |  |  | 501-750 | 63.46% | 80.71% | 71.06% |
|  |  |  | 751-1000 | 63.78% | 79.00% | 70.58% |
|  |  |  | 1000-5000 | 66.12% | 70.23% | 68.11% |
|  |  |  | >5000 | 74.59% | 68.06% | 71.18% |
|  |  | SVIM | 50-250 | 70.61% | 58.93% | 64.24% |
|  |  |  | 251-500 | 67.78% | 53.27% | 59.65% |
|  |  |  | 501-750 | 64.94% | 72.63% | 68.57% |
|  |  |  | 751-1000 | 64.67% | 71.05% | 67.71% |
|  |  |  | 1000-5000 | 69.13% | 60.49% | 64.52% |
|  |  |  | >5000 | 84.17% | 63.07% | 72.11% |
|  |  | PBSV | 50-250 | 70.10% | 62.81% | 66.25% |
|  |  |  | 251-500 | 69.47% | 57.80% | 63.10% |
|  |  |  | 501-750 | 66.76% | 77.47% | 71.71% |
|  |  |  | 751-1000 | 65.60% | 74.23% | 69.65% |
|  |  |  | 1000-5000 | 62.57% | 63.77% | 63.16% |
|  |  |  | >5000 | 80.80% | 62.94% | 70.76% |
|  |  | SVDSS | 50-250 | 87.24% | 87.55% | 87.39% |
|  |  |  | 251-500 | 88.97% | 86.28% | 87.61% |
|  |  |  | 501-750 | 87.71% | 92.68% | 90.13% |
|  |  |  | 751-1000 | 88.01% | 91.09% | 89.53% |
|  |  |  | 1000-5000 | 91.38% | 88.80% | 90.07% |
|  |  |  | >5000 | 94.50% | 88.01% | 91.14% |
|  | 20X | Sniffles | 50-250 | 94.26% | 39.37% | 55.54% |
|  |  |  | 251-500 | 97.62% | 45.72% | 62.28% |
|  |  |  | 501-750 | 89.85% | 51.10% | 65.15% |
|  |  |  | 751-1000 | 88.99% | 55.34% | 68.24% |
|  |  |  | 1000-5000 | 100.00% | 100.00% | 100.00% |
|  |  |  | >5000 | 99.19% | 67.40% | 80.26% |
|  |  | Cutesv | 50-250 | 74.65% | 78.10% | 76.34% |
|  |  |  | 251-500 | 71.22% | 74.62% | 72.88% |
|  |  |  | 501-750 | 69.67% | 85.55% | 76.80% |
|  |  |  | 751-1000 | 72.35% | 84.09% | 77.78% |
|  |  |  | 1000-5000 | 75.35% | 78.64% | 76.96% |
|  |  |  | >5000 | 86.62% | 77.63% | 81.88% |
|  |  | SVIM | 50-250 | 81.85% | 69.30% | 75.06% |
|  |  |  | 251-500 | 78.83% | 62.61% | 69.79% |
|  |  |  | 501-750 | 73.68% | 78.42% | 75.98% |
|  |  |  | 751-1000 | 73.19% | 77.31% | 75.19% |
|  |  |  | 1000-5000 | 79.21% | 70.99% | 74.88% |
|  |  |  | >5000 | 93.47% | 75.20% | 83.35% |
|  |  | PBSV | 50-250 | 78.19% | 70.69% | 74.25% |
|  |  |  | 251-500 | 78.18% | 65.58% | 71.33% |
|  |  |  | 501-750 | 73.13% | 80.71% | 76.73% |
|  |  |  | 751-1000 | 74.45% | 79.11% | 76.71% |
|  |  |  | 1000-5000 | 77.19% | 72.88% | 74.97% |
|  |  |  | >5000 | 89.13% | 72.91% | 80.21% |
|  |  | SVDSS | 50-250 | 99.86% | 99.96% | 99.91% |
|  |  |  | 251-500 | 99.95% | 99.98% | 99.97% |
|  |  |  | 501-750 | 96.50% | 100.00% | 98.22% |
|  |  |  | 751-1000 | 83.23% | 100.00% | 90.85% |
|  |  |  | 1000-5000 | 93.16% | 99.86% | 96.39% |
|  |  |  | >5000 | 95.99% | 100.00% | 97.95% |
|  | 10X | Sniffles | 50-250 | 94.91% | 12.27% | 21.73% |
|  |  |  | 251-500 | 99.33% | 80.80% | 89.11% |
|  |  |  | 501-750 | 96.27% | 78.95% | 86.75% |
|  |  |  | 751-1000 | 95.54% | 82.19% | 88.37% |
|  |  |  | 1000-5000 | 100.00% | 100.00% | 100.00% |
|  |  |  | >5000 | 99.70% | 92.05% | 95.73% |
|  |  | Cutesv | 50-250 | 92.40% | 93.01% | 92.71% |
|  |  |  | 251-500 | 89.89% | 90.29% | 90.09% |
|  |  |  | 501-750 | 86.44% | 95.35% | 90.68% |
|  |  |  | 751-1000 | 89.28% | 94.49% | 91.81% |
|  |  |  | 1000-5000 | 91.23% | 92.95% | 92.08% |
|  |  |  | >5000 | 97.09% | 94.47% | 95.77% |
|  |  | SVIM | 50-250 | 95.98% | 90.04% | 92.92% |
|  |  |  | 251-500 | 94.73% | 85.94% | 90.13% |
|  |  |  | 501-750 | 89.83% | 91.66% | 90.74% |
|  |  |  | 751-1000 | 89.74% | 91.83% | 90.78% |
|  |  |  | 1000-5000 | 93.96% | 90.68% | 92.29% |
|  |  |  | >5000 | 98.16% | 93.67% | 95.86% |
|  |  | PBSV | 50-250 | 93.84% | 89.61% | 91.67% |
|  |  |  | 251-500 | 93.66% | 86.98% | 90.19% |
|  |  |  | 501-750 | 89.53% | 92.49% | 90.98% |
|  |  |  | 751-1000 | 90.68% | 91.83% | 91.25% |
|  |  |  | 1000-5000 | 93.11% | 90.99% | 92.04% |
|  |  |  | >5000 | 96.37% | 93.13% | 94.72% |
|  |  | SVDSS | 50-250 | 78.02% | 77.00% | 77.51% |
|  |  |  | 251-500 | 78.86% | 72.78% | 75.70% |
|  |  |  | 501-750 | 75.80% | 84.53% | 79.93% |
|  |  |  | 751-1000 | 76.00% | 82.93% | 79.31% |
|  |  |  | 1000-5000 | 80.60% | 76.58% | 78.54% |
|  |  |  | >5000 | 87.28% | 78.57% | 82.70% |

**Table S12** The precision, recall, and F-score values for SV calling for the NA24385 sample with Sniffles, SVIM, CuteSV, PBSV and SVDSS following Alignment with NGMLR at different depths of coverage across the SV length groups

| Aligner | Coverage | SV caller | SV length groups | Precision | Recall | F1-score |
| --- | --- | --- | --- | --- | --- | --- |
| NGMLR | total | Sniffles | 50-250 | 57.15% | 60.16% | 58.62% |
|  |  |  | 251-500 | 85.69% | 33.93% | 48.61% |
|  |  |  | 501-750 | 71.26% | 51.78% | 59.98% |
|  |  |  | 751-1000 | 74.80% | 50.41% | 60.23% |
|  |  |  | 1000-5000 | 99.05% | 98.47% | 98.76% |
|  |  |  | >5000 | 93.43% | 54.52% | 68.86% |
|  |  | Cutesv | 50-250 | 62.07% | 68.70% | 65.22% |
|  |  |  | 251-500 | 60.51% | 66.77% | 63.49% |
|  |  |  | 501-750 | 60.53% | 76.83% | 67.71% |
|  |  |  | 751-1000 | 66.67% | 77.84% | 71.82% |
|  |  |  | 1000-5000 | 71.50% | 69.53% | 70.50% |
|  |  |  | >5000 | 75.29% | 70.22% | 72.66% |
|  |  | SVIM | 50-250 | 64.13% | 58.12% | 60.98% |
|  |  |  | 251-500 | 65.37% | 54.96% | 59.72% |
|  |  |  | 501-750 | 63.00% | 71.87% | 67.14% |
|  |  |  | 751-1000 | 69.21% | 74.13% | 71.58% |
|  |  |  | 1000-5000 | 82.92% | 69.98% | 75.90% |
|  |  |  | >5000 | 98.04% | 74.26% | 84.51% |
|  |  | PBSV | 50-250 | 68.92% | 61.96% | 65.25% |
|  |  |  | 251-500 | 71.84% | 62.27% | 66.71% |
|  |  |  | 501-750 | 61.67% | 74.67% | 67.55% |
|  |  |  | 751-1000 | 64.00% | 73.70% | 68.51% |
|  |  |  | 1000-5000 | 69.68% | 64.01% | 66.73% |
|  |  |  | >5000 | 80.83% | 65.90% | 72.61% |
|  |  | SVDSS | 50-250 | 58.21% | 59.23% | 58.71% |
|  |  |  | 251-500 | 70.80% | 61.11% | 65.60% |
|  |  |  | 501-750 | 70.15% | 78.55% | 74.11% |
|  |  |  | 751-1000 | 71.90% | 76.25% | 74.01% |
|  |  |  | 1000-5000 | 84.31% | 75.01% | 79.39% |
|  |  |  | >5000 | 100.00% | 99.87% | 99.93% |
|  | 30X | Sniffles | 50-250 | 92.33% | 63.00% | 74.90% |
|  |  |  | 251-500 | 89.01% | 37.50% | 52.77% |
|  |  |  | 501-750 | 77.40% | 54.67% | 64.08% |
|  |  |  | 751-1000 | 79.84% | 53.15% | 63.82% |
|  |  |  | 1000-5000 | 98.61% | 97.90% | 98.25% |
|  |  |  | >5000 | 95.48% | 57.81% | 72.01% |
|  |  | Cutesv | 50-250 | 65.80% | 70.56% | 68.10% |
|  |  |  | 251-500 | 64.59% | 68.47% | 66.47% |
|  |  |  | 501-750 | 64.58% | 78.23% | 70.75% |
|  |  |  | 751-1000 | 70.95% | 78.47% | 74.52% |
|  |  |  | 1000-5000 | 76.97% | 72.57% | 74.70% |
|  |  |  | >5000 | 81.38% | 74.80% | 77.95% |
|  |  | SVIM | 50-250 | 70.77% | 61.43% | 65.77% |
|  |  |  | 251-500 | 74.48% | 60.46% | 66.74% |
|  |  |  | 501-750 | 69.47% | 75.62% | 72.42% |
|  |  |  | 751-1000 | 72.65% | 76.35% | 74.46% |
|  |  |  | 1000-5000 | 88.88% | 74.76% | 81.21% |
|  |  |  | >5000 | 98.67% | 79.92% | 88.31% |
|  |  | PBSV | 50-250 | 71.80% | 63.94% | 67.64% |
|  |  |  | 251-500 | 77.28% | 65.71% | 71.03% |
|  |  |  | 501-750 | 66.37% | 77.02% | 71.30% |
|  |  |  | 751-1000 | 68.08% | 74.87% | 71.31% |
|  |  |  | 1000-5000 | 74.63% | 66.95% | 70.58% |
|  |  |  | >5000 | 86.39% | 71.83% | 78.44% |
|  |  | SVDSS | 50-250 | 74.83% | 74.31% | 74.57% |
|  |  |  | 251-500 | 83.66% | 73.94% | 78.50% |
|  |  |  | 501-750 | 83.20% | 88.61% | 85.82% |
|  |  |  | 751-1000 | 84.48% | 84.31% | 84.39% |
|  |  |  | 1000-5000 | 92.62% | 84.15% | 88.19% |
|  |  |  | >5000 | 98.02% | 97.63% | 97.82% |
|  | 20X | Sniffles | 50-250 | 86.97% | 67.67% | 76.12% |
|  |  |  | 251-500 | 93.85% | 53.16% | 67.87% |
|  |  |  | 501-750 | 83.37% | 62.99% | 71.76% |
|  |  |  | 751-1000 | 86.97% | 67.67% | 76.12% |
|  |  |  | 1000-5000 | 97.90% | 97.48% | 97.69% |
|  |  |  | >5000 | 98.83% | 95.85% | 97.32% |
|  |  | Cutesv | 50-250 | 76.34% | 78.19% | 77.26% |
|  |  |  | 251-500 | 75.21% | 75.79% | 75.50% |
|  |  |  | 501-750 | 73.47% | 84.60% | 78.64% |
|  |  |  | 751-1000 | 78.21% | 84.52% | 81.24% |
|  |  |  | 1000-5000 | 85.39% | 81.57% | 83.43% |
|  |  |  | >5000 | 89.67% | 84.23% | 86.87% |
|  |  | SVIM | 50-250 | 82.35% | 72.03% | 76.85% |
|  |  |  | 251-500 | 86.07% | 71.89% | 78.34% |
|  |  |  | 501-750 | 79.26% | 82.24% | 80.72% |
|  |  |  | 751-1000 | 82.49% | 83.46% | 82.97% |
|  |  |  | 1000-5000 | 93.54% | 83.42% | 88.19% |
|  |  |  | >5000 | 99.55% | 89.49% | 94.25% |
|  |  | PBSV | 50-250 | 80.11% | 72.16% | 75.93% |
|  |  |  | 251-500 | 86.67% | 74.81% | 80.30% |
|  |  |  | 501-750 | 76.65% | 82.75% | 79.58% |
|  |  |  | 751-1000 | 77.03% | 80.38% | 78.67% |
|  |  |  | 1000-5000 | 83.49% | 76.96% | 80.09% |
|  |  |  | >5000 | 92.38% | 84.91% | 88.48% |
|  |  | SVDSS | 50-250 | 71.81% | 70.24% | 71.01% |
|  |  |  | 251-500 | 82.90% | 71.42% | 76.73% |
|  |  |  | 501-750 | 80.76% | 85.23% | 82.94% |
|  |  |  | 751-1000 | 82.97% | 83.67% | 83.32% |
|  |  |  | 1000-5000 | 90.73% | 82.02% | 86.16% |
|  |  |  | >5000 | 93.46% | 92.59% | 93.02% |
|  | 10X | Sniffles | 50-250 | 74.80% | 51.21% | 60.80% |
|  |  |  | 251-500 | 99.20% | 84.10% | 91.02% |
|  |  |  | 501-750 | 96.42% | 86.76% | 91.33% |
|  |  |  | 751-1000 | 95.22% | 87.40% | 91.14% |
|  |  |  | 1000-5000 | 98.69% | 98.05% | 98.37% |
|  |  |  | >5000 | 100.00% | 93.15% | 96.45% |
|  |  | Cutesv | 50-250 | 94.05% | 93.74% | 93.89% |
|  |  |  | 251-500 | 93.69% | 91.71% | 92.69% |
|  |  |  | 501-750 | 92.33% | 96.56% | 94.40% |
|  |  |  | 751-1000 | 94.53% | 95.23% | 94.88% |
|  |  |  | 1000-5000 | 97.09% | 95.36% | 96.21% |
|  |  |  | >5000 | 98.49% | 96.63% | 97.55% |
|  |  | SVIM | 50-250 | 96.31% | 91.88% | 94.04% |
|  |  |  | 251-500 | 97.52% | 91.22% | 94.26% |
|  |  |  | 501-750 | 94.98% | 95.10% | 95.04% |
|  |  |  | 751-1000 | 96.53% | 94.27% | 95.39% |
|  |  |  | 1000-5000 | 98.85% | 96.16% | 97.49% |
|  |  |  | >5000 | 100.00% | 97.44% | 98.70% |
|  |  | PBSV | 50-250 | 94.92% | 91.40% | 93.13% |
|  |  |  | 251-500 | 97.64% | 91.64% | 94.55% |
|  |  |  | 501-750 | 93.78% | 95.04% | 94.40% |
|  |  |  | 751-1000 | 94.14% | 93.64% | 93.89% |
|  |  |  | 1000-5000 | 96.20% | 94.52% | 95.35% |
|  |  |  | >5000 | 98.26% | 98.92% | 98.59% |
|  |  | SVDSS | 50-250 | 85.52% | 83.69% | 84.59% |
|  |  |  | 251-500 | 92.35% | 82.77% | 87.29% |
|  |  |  | 501-750 | 89.16% | 92.17% | 90.64% |
|  |  |  | 751-1000 | 91.16% | 90.77% | 90.97% |
|  |  |  | 1000-5000 | 95.65% | 90.51% | 93.01% |
|  |  |  | >5000 | 87.39% | 92.72% | 89.97% |

**Table S13** The precision, recall, and F-score values for SV calling for the NA24385 sample with Sniffles, SVIM, CuteSV, PBSV and SVDSS following Alignment with Pbmm2 at different depths of coverage across the SV length groups

| Aligner | Coverage | SV caller | SV length groups | Precision | Recall | F1-score |
| --- | --- | --- | --- | --- | --- | --- |
| Pbmm2 | total | Sniffles | 50-250 | 85.26% | 40.90% | 55.28% |
|  |  |  | 251-500 | 91.99% | 29.41% | 44.57% |
|  |  |  | 501-750 | 82.20% | 43.12% | 56.57% |
|  |  |  | 751-1000 | 83.33% | 46.58% | 59.75% |
|  |  |  | 1000-5000 | 92.91% | 87.10% | 89.91% |
|  |  |  | >5000 | 87.86% | 49.59% | 63.40% |
|  |  | Cutesv | 50-250 | 74.65% | 78.10% | 76.34% |
|  |  |  | 251-500 | 71.22% | 74.62% | 72.88% |
|  |  |  | 501-750 | 69.67% | 85.55% | 76.80% |
|  |  |  | 751-1000 | 72.35% | 84.09% | 77.78% |
|  |  |  | 1000-5000 | 75.35% | 78.64% | 76.96% |
|  |  |  | >5000 | 86.62% | 77.63% | 81.88% |
|  |  | SVIM | 50-250 | 60.33% | 54.14% | 57.07% |
|  |  |  | 251-500 | 63.69% | 51.94% | 57.22% |
|  |  |  | 501-750 | 63.63% | 72.37% | 67.72% |
|  |  |  | 751-1000 | 64.80% | 71.05% | 67.78% |
|  |  |  | 1000-5000 | 74.06% | 64.36% | 68.87% |
|  |  |  | >5000 | 83.07% | 64.82% | 72.82% |
|  |  | PBSV | 50-250 | 63.89% | 60.87% | 62.34% |
|  |  |  | 251-500 | 66.39% | 56.50% | 61.05% |
|  |  |  | 501-750 | 64.75% | 77.40% | 70.51% |
|  |  |  | 751-1000 | 66.26% | 74.97% | 70.35% |
|  |  |  | 1000-5000 | 71.14% | 64.08% | 67.43% |
|  |  |  | >5000 | 80.21% | 62.26% | 70.11% |
|  |  | SVDSS | 50-250 | 73.47% | 71.64% | 72.54% |
|  |  |  | 251-500 | 76.40% | 67.53% | 71.69% |
|  |  |  | 501-750 | 76.49% | 84.28% | 80.19% |
|  |  |  | 751-1000 | 78.23% | 83.46% | 80.76% |
|  |  |  | 1000-5000 | 83.54% | 75.64% | 79.39% |
|  |  |  | >5000 | 94.18% | 80.73% | 86.94% |
|  | 30X | Sniffles | 50-250 | 91.25% | 46.44% | 61.56% |
|  |  |  | 251-500 | 94.42% | 34.59% | 50.63% |
|  |  |  | 501-750 | 87.88% | 49.24% | 63.11% |
|  |  |  | 751-1000 | 87.91% | 51.78% | 65.17% |
|  |  |  | 1000-5000 | 92.78% | 94.73% | 93.75% |
|  |  |  | >5000 | 99.57% | 63.01% | 77.18% |
|  |  | Cutesv | 50-250 | 63.47% | 70.78% | 66.93% |
|  |  |  | 251-500 | 64.11% | 70.42% | 67.12% |
|  |  |  | 501-750 | 65.90% | 82.30% | 73.20% |
|  |  |  | 751-1000 | 67.64% | 81.34% | 73.86% |
|  |  |  | 1000-5000 | 74.04% | 73.86% | 73.95% |
|  |  |  | >5000 | 77.91% | 71.29% | 74.45% |
|  |  | SVIM | 50-250 | 69.80% | 59.11% | 64.01% |
|  |  |  | 251-500 | 71.81% | 55.90% | 62.86% |
|  |  |  | 501-750 | 71.63% | 76.00% | 73.75% |
|  |  |  | 751-1000 | 71.19% | 72.85% | 72.01% |
|  |  |  | 1000-5000 | 80.77% | 70.65% | 75.37% |
|  |  |  | >5000 | 89.14% | 73.05% | 80.30% |
|  |  | PBSV | 50-250 | 68.13% | 63.04% | 65.48% |
|  |  |  | 251-500 | 71.49% | 59.74% | 65.09% |
|  |  |  | 501-750 | 69.95% | 78.55% | 74.00% |
|  |  |  | 751-1000 | 71.68% | 76.78% | 74.14% |
|  |  |  | 1000-5000 | 77.70% | 69.18% | 73.19% |
|  |  |  | >5000 | 85.69% | 66.98% | 75.19% |
|  |  | SVDSS | 50-250 | 81.38% | 79.22% | 80.29% |
|  |  |  | 251-500 | 83.70% | 74.92% | 79.07% |
|  |  |  | 501-750 | 83.60% | 87.91% | 85.70% |
|  |  |  | 751-1000 | 84.87% | 88.02% | 86.41% |
|  |  |  | 1000-5000 | 89.01% | 82.51% | 85.64% |
|  |  |  | >5000 | 96.52% | 85.85% | 90.87% |
|  | 20X | Sniffles | 50-250 | 95.41% | 59.26% | 73.11% |
|  |  |  | 251-500 | 97.15% | 48.26% | 64.48% |
|  |  |  | 501-750 | 90.86% | 57.39% | 70.34% |
|  |  |  | 751-1000 | 91.20% | 62.47% | 74.15% |
|  |  |  | 1000-5000 | 97.48% | 92.75% | 95.06% |
|  |  |  | >5000 | 78.47% | 75.89% | 77.16% |
|  |  | Cutesv | 50-250 | 73.08% | 77.44% | 75.20% |
|  |  |  | 251-500 | 72.36% | 76.29% | 74.28% |
|  |  |  | 501-750 | 71.67% | 86.95% | 78.57% |
|  |  |  | 751-1000 | 75.53% | 86.43% | 80.61% |
|  |  |  | 1000-5000 | 80.84% | 81.88% | 81.36% |
|  |  |  | >5000 | 85.18% | 79.78% | 82.39% |
|  |  | SVIM | 50-250 | 80.73% | 68.68% | 74.22% |
|  |  |  | 251-500 | 81.60% | 65.11% | 72.43% |
|  |  |  | 501-750 | 77.75% | 80.97% | 79.33% |
|  |  |  | 751-1000 | 79.65% | 80.91% | 80.27% |
|  |  |  | 1000-5000 | 87.13% | 79.13% | 82.93% |
|  |  |  | >5000 | 93.18% | 81.00% | 86.66% |
|  |  | PBSV | 50-250 | 76.73% | 70.81% | 73.65% |
|  |  |  | 251-500 | 79.60% | 67.89% | 73.28% |
|  |  |  | 501-750 | 76.84% | 83.64% | 80.10% |
|  |  |  | 751-1000 | 79.41% | 83.03% | 81.18% |
|  |  |  | 1000-5000 | 84.90% | 77.35% | 80.95% |
|  |  |  | >5000 | 89.70% | 76.28% | 82.45% |
|  |  | SVDSS | 50-250 | 87.04% | 85.56% | 86.29% |
|  |  |  | 251-500 | 88.75% | 81.49% | 84.97% |
|  |  |  | 501-750 | 88.18% | 90.71% | 89.43% |
|  |  |  | 751-1000 | 89.45% | 91.73% | 90.58% |
|  |  |  | 1000-5000 | 92.22% | 87.75% | 89.93% |
|  |  |  | >5000 | 97.36% | 89.62% | 93.33% |
|  | 10X | Sniffles | 50-250 | 99.22% | 87.16% | 92.80% |
|  |  |  | 251-500 | 99.54% | 82.73% | 90.36% |
|  |  |  | 501-750 | 97.65% | 84.55% | 90.63% |
|  |  |  | 751-1000 | 98.20% | 89.59% | 93.70% |
|  |  |  | 1000-5000 | 89.93% | 89.45% | 89.69% |
|  |  |  | >5000 | 86.80% | 97.26% | 91.73% |
|  |  | Cutesv | 50-250 | 92.40% | 93.08% | 92.74% |
|  |  |  | 251-500 | 91.56% | 91.54% | 91.55% |
|  |  |  | 501-750 | 90.02% | 96.44% | 93.12% |
|  |  |  | 751-1000 | 92.49% | 96.71% | 94.56% |
|  |  |  | 1000-5000 | 95.33% | 95.39% | 95.36% |
|  |  |  | >5000 | 97.25% | 95.28% | 96.26% |
|  |  | SVIM | 50-250 | 96.10% | 90.37% | 93.14% |
|  |  |  | 251-500 | 95.61% | 88.30% | 91.81% |
|  |  |  | 501-750 | 93.73% | 94.14% | 93.93% |
|  |  |  | 751-1000 | 94.30% | 94.80% | 94.55% |
|  |  |  | 1000-5000 | 97.43% | 95.18% | 96.29% |
|  |  |  | >5000 | 98.35% | 96.23% | 97.28% |
|  |  | PBSV | 50-250 | 93.64% | 90.51% | 92.05% |
|  |  |  | 251-500 | 95.41% | 89.12% | 92.16% |
|  |  |  | 501-750 | 92.83% | 94.84% | 93.83% |
|  |  |  | 751-1000 | 94.71% | 94.91% | 94.81% |
|  |  |  | 1000-5000 | 96.65% | 94.62% | 95.63% |
|  |  |  | >5000 | 97.80% | 95.82% | 96.80% |
|  |  | SVDSS | 50-250 | 94.73% | 94.41% | 94.57% |
|  |  |  | 251-500 | 95.58% | 92.06% | 93.79% |
|  |  |  | 501-750 | 95.07% | 95.80% | 95.43% |
|  |  |  | 751-1000 | 95.41% | 97.03% | 96.21% |
|  |  |  | 1000-5000 | 96.98% | 95.25% | 96.11% |
|  |  |  | >5000 | 99.44% | 95.55% | 97.46% |


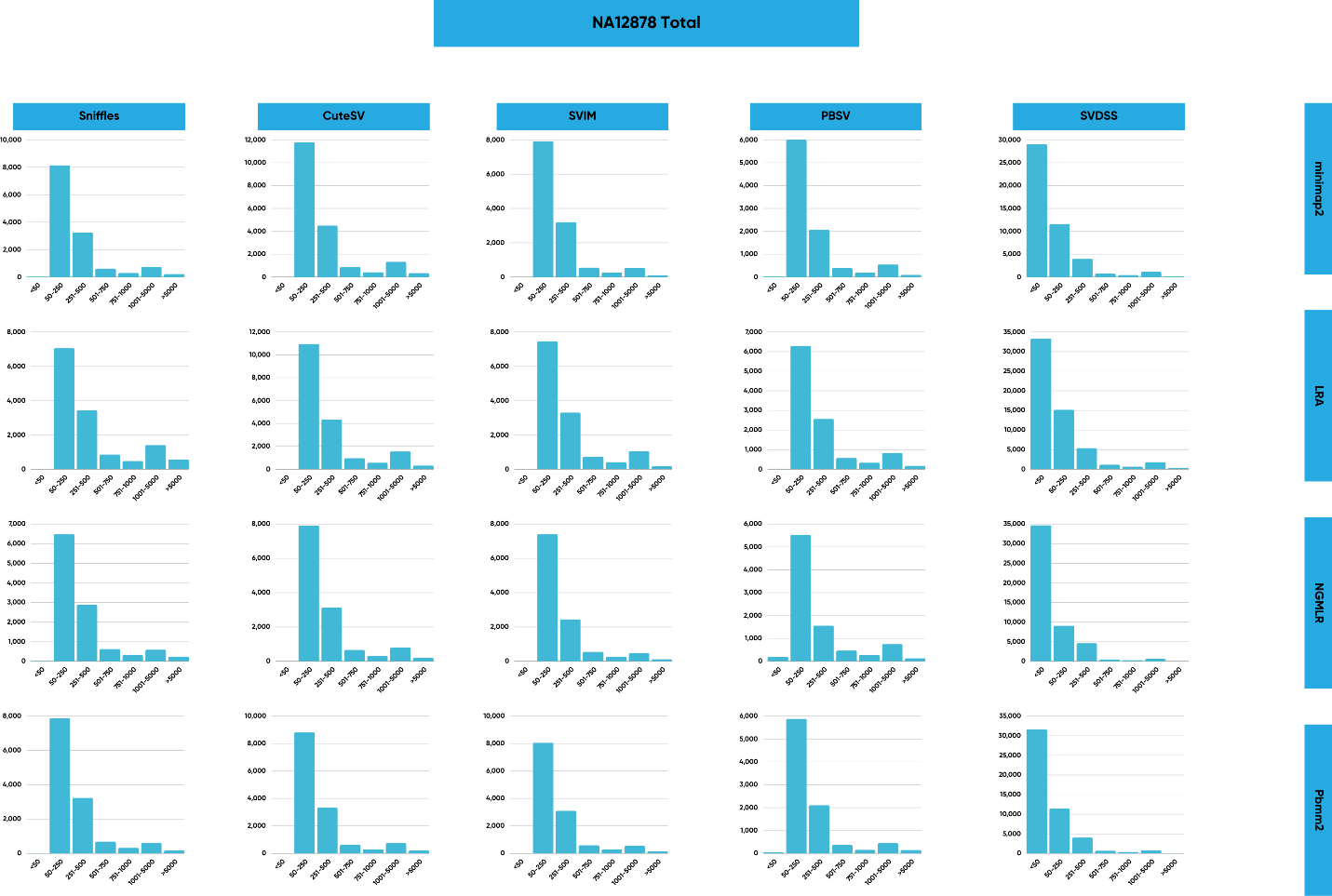


**Figure S1.** The chart bar represents the SV count for sample NA12878 at total coverage for all the SV callers (CuteSV, Sniffles, SVIM, PBSV and SVDSS) with the different aligners (Minimap2, LRA, Ngmlr and Pbmm2)


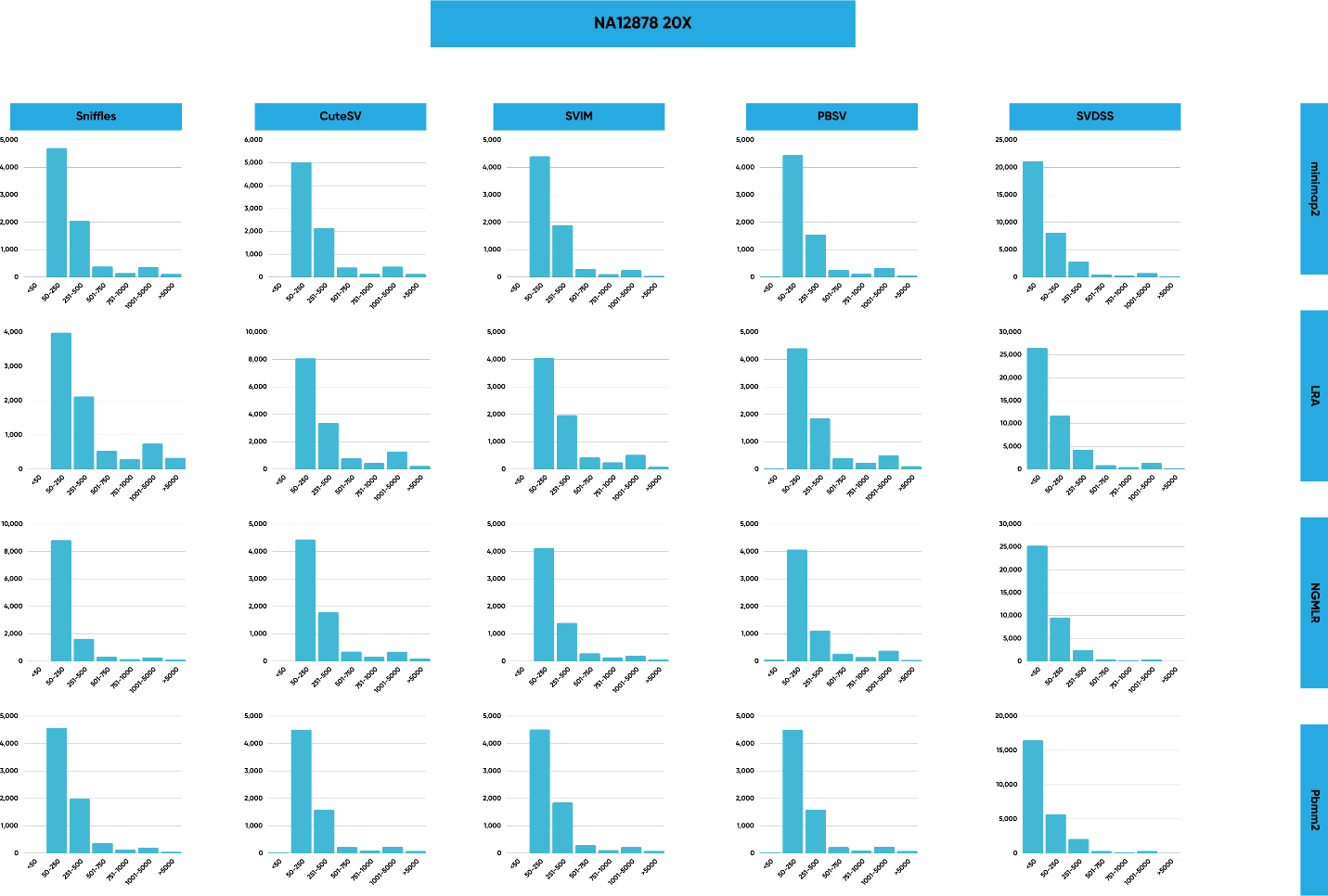


**Figure S2**. The chart bar represents the SV count for sample NA12878 at 20X coverage for all the SV callers (CuteSV, Sniffles, SVIM, PBSV and SVDSS) with the different aligners (Minimap2, LRA, Ngmlr and Pbmm2)


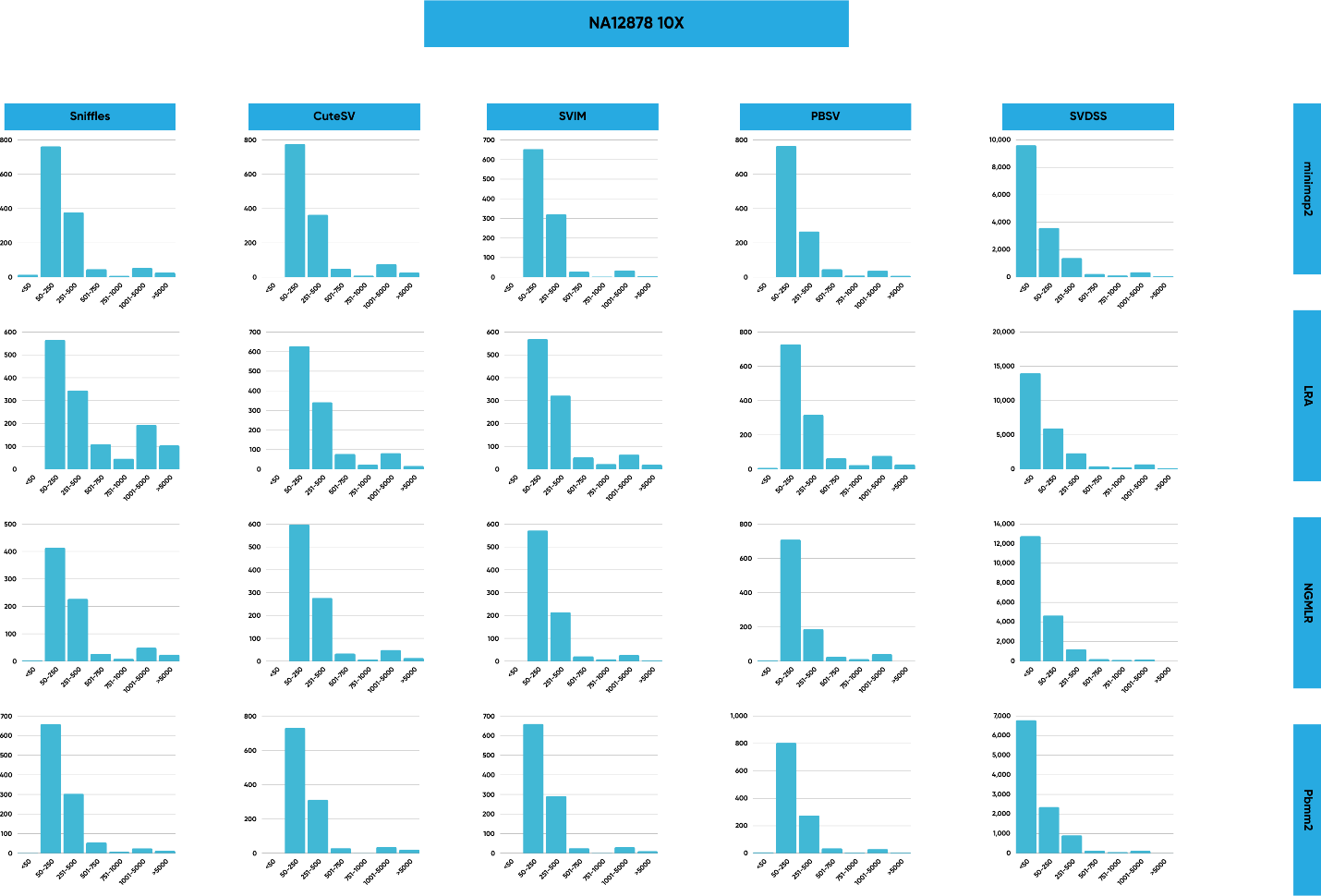


**Figure S3.** The chart bar represents the SV count for sample NA12878 at 10X coverage for all the SV callers (CuteSV, Sniffles, SVIM, PBSV and SVDSS) with the different aligners (Minimap2, LRA, Ngmlr and Pbmm2)


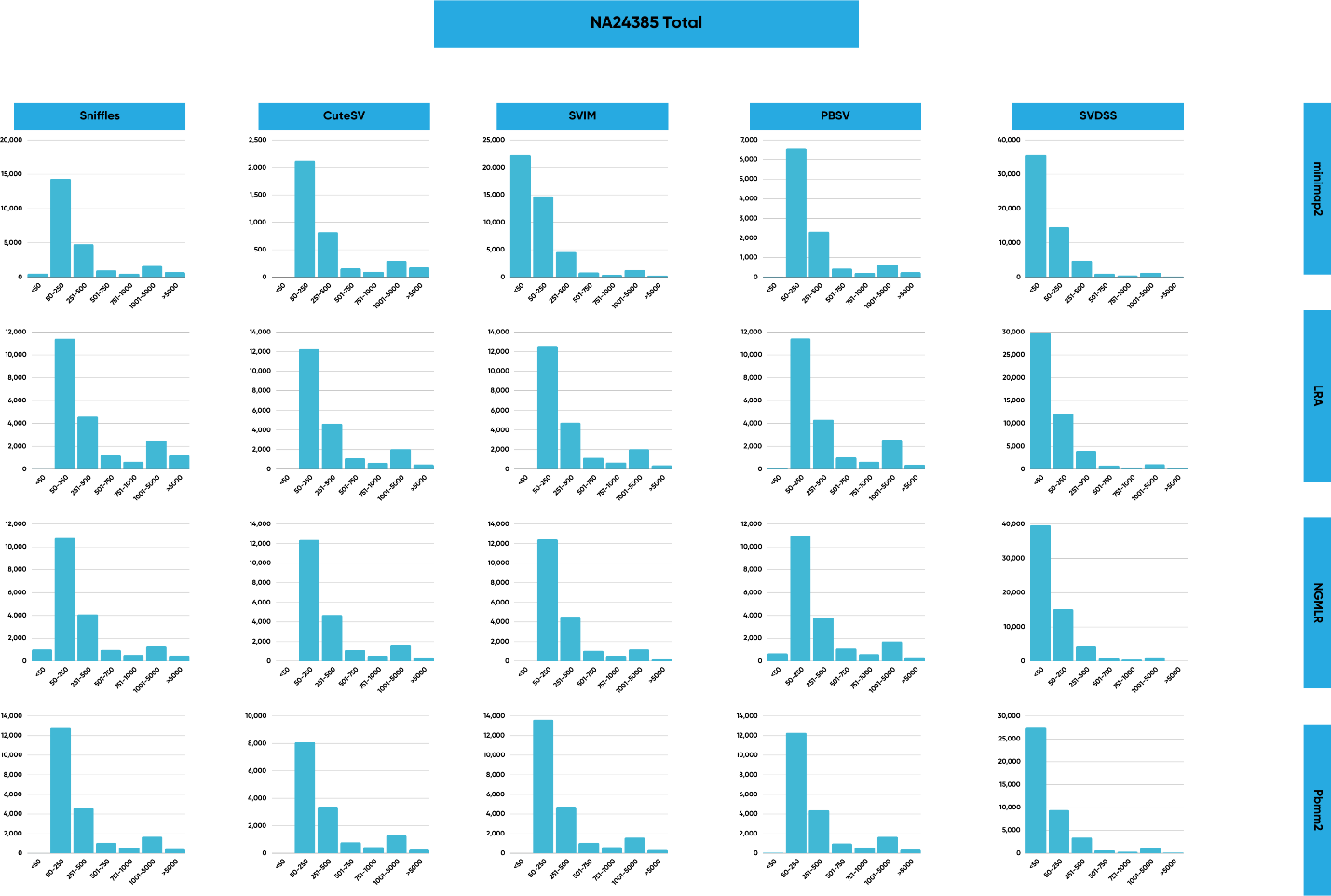


**Figure S4**. The chart bar represents the SV count for sample NA24385 at total coverage for all the SV callers (CuteSV, Sniffles, SVIM, PBSV and SVDSS) with the different aligners (Minimap2, LRA, Ngmlr and Pbmm2)


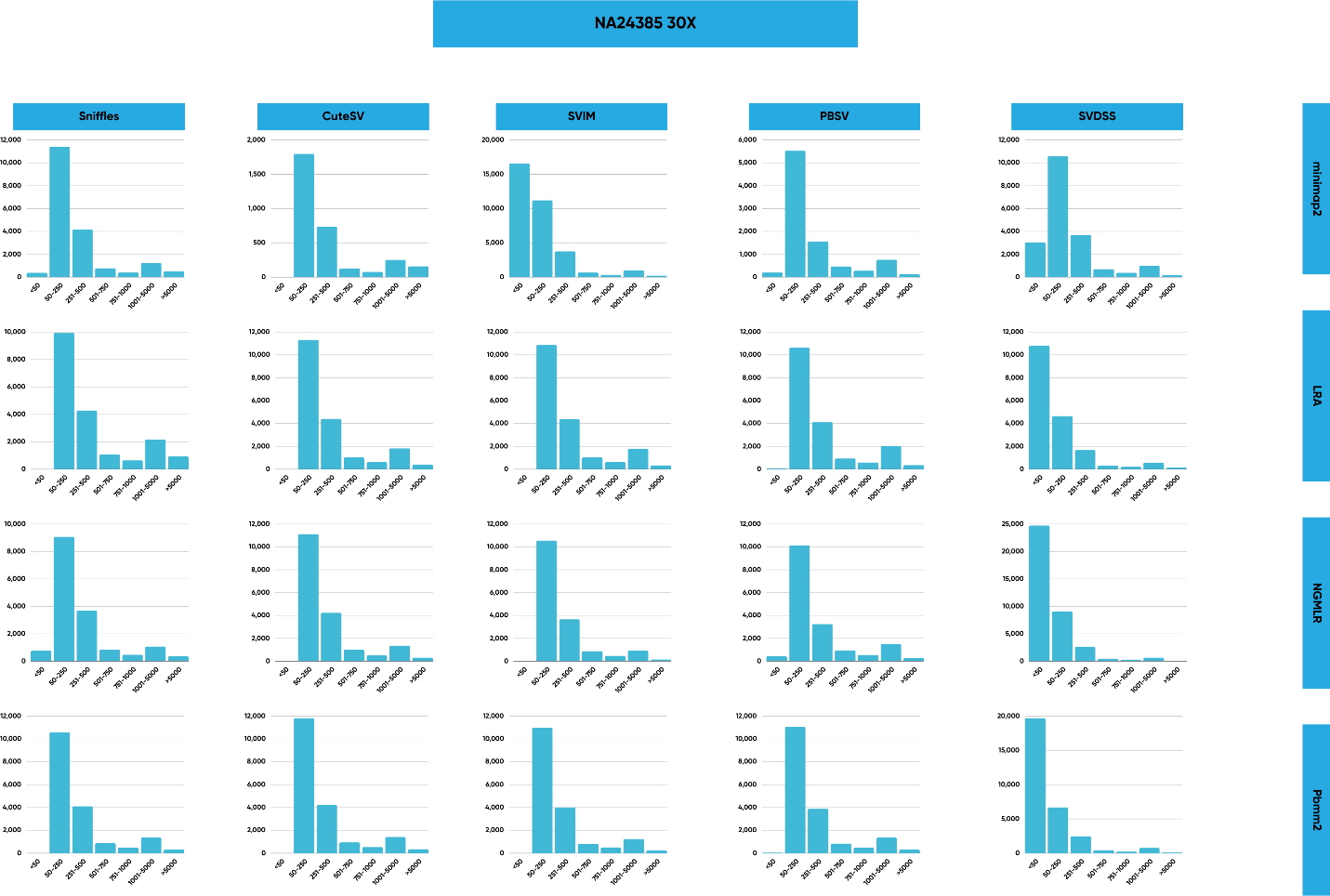


**Figure S5.** The chart bar represents the SV count for sample NA24385 at 30X coverage for all the SV callers (CuteSV, Sniffles, SVIM, PBSV and SVDSS) with the different aligners (Minimap2, LRA, Ngmlr and Pbmm2)


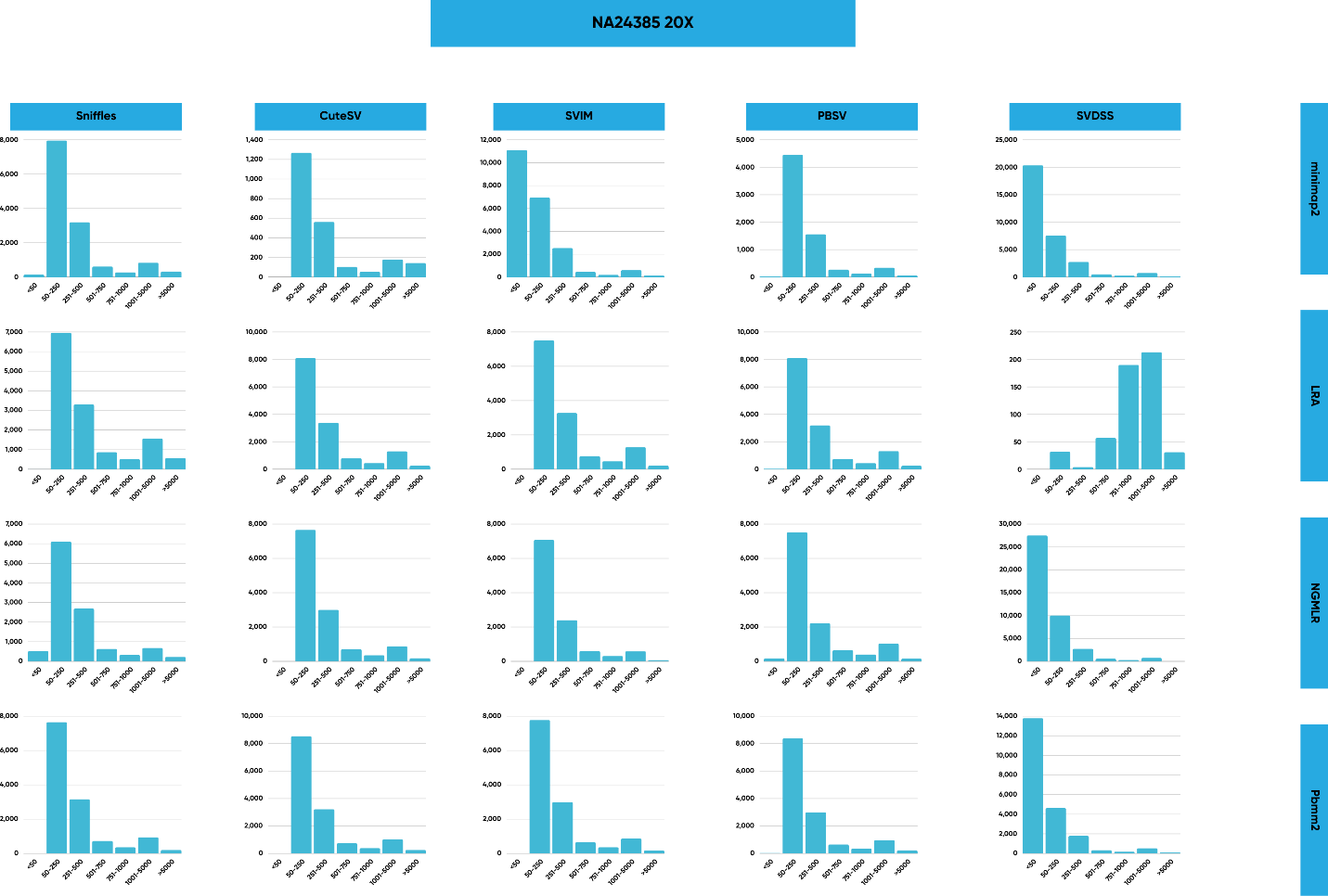


**Figure S6**. The chart bar represents the SV count for sample NA24385 at 20X coverage for all the SV callers (CuteSV, Sniffles, SVIM, PBSV and SVDSS) with the different aligners (Minimap2, LRA, Ngmlr and Pbmm2)


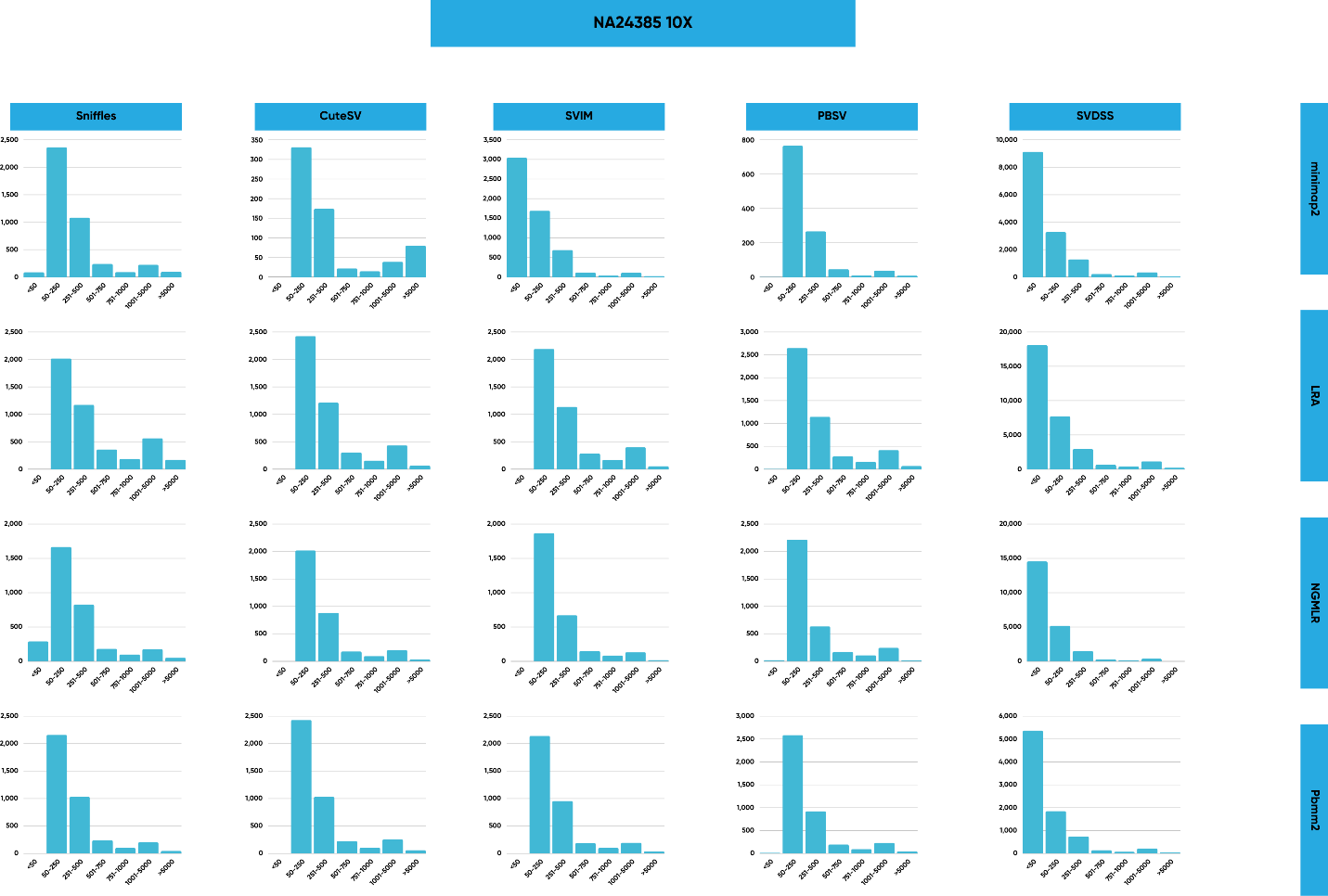


**Figure S7**. The chart bar represents the SV count for sample NA24385 at 10X coverage for all the SV callers (CuteSV, Sniffles, SVIM, PBSV and SVDSS) with the different aligners (Minimap2, LRA, Ngmlr and Pbmm2)


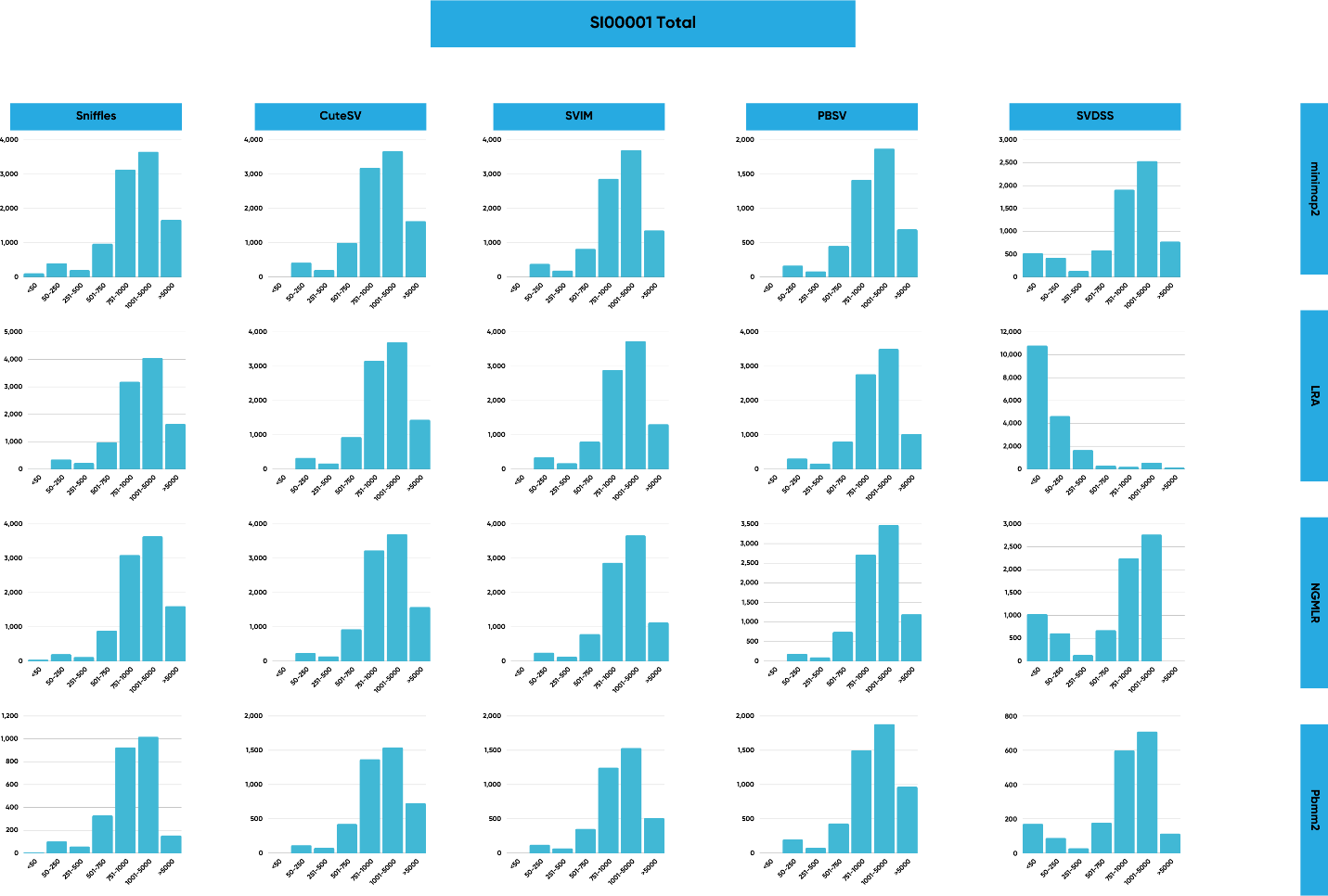


**Figure S8**. The chart bar represents the SV count for sample SI00001 at total coverage for all the SV callers (CuteSV, Sniffles, SVIM, PBSV and SVDSS) with the different aligners (Minimap2, LRA, Ngmlr and Pbmm2)


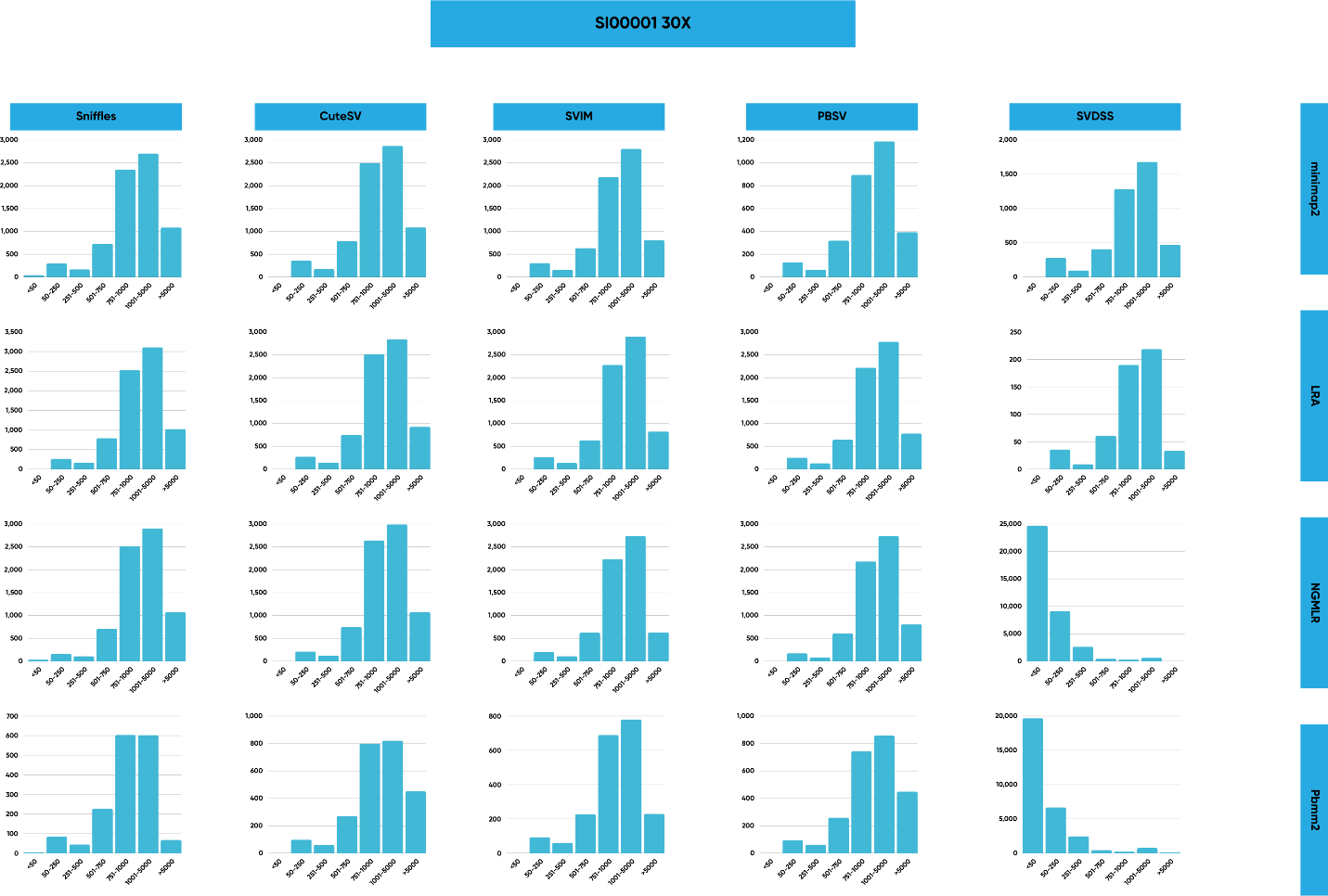


**Figure S9.** The chart bar represents the SV count for sample SI00001 at 30X coverage for all the SV callers (CuteSV, Sniffles, SVIM, PBSV and SVDSS) with the different aligners (Minimap2, LRA, Ngmlr and Pbmm2)


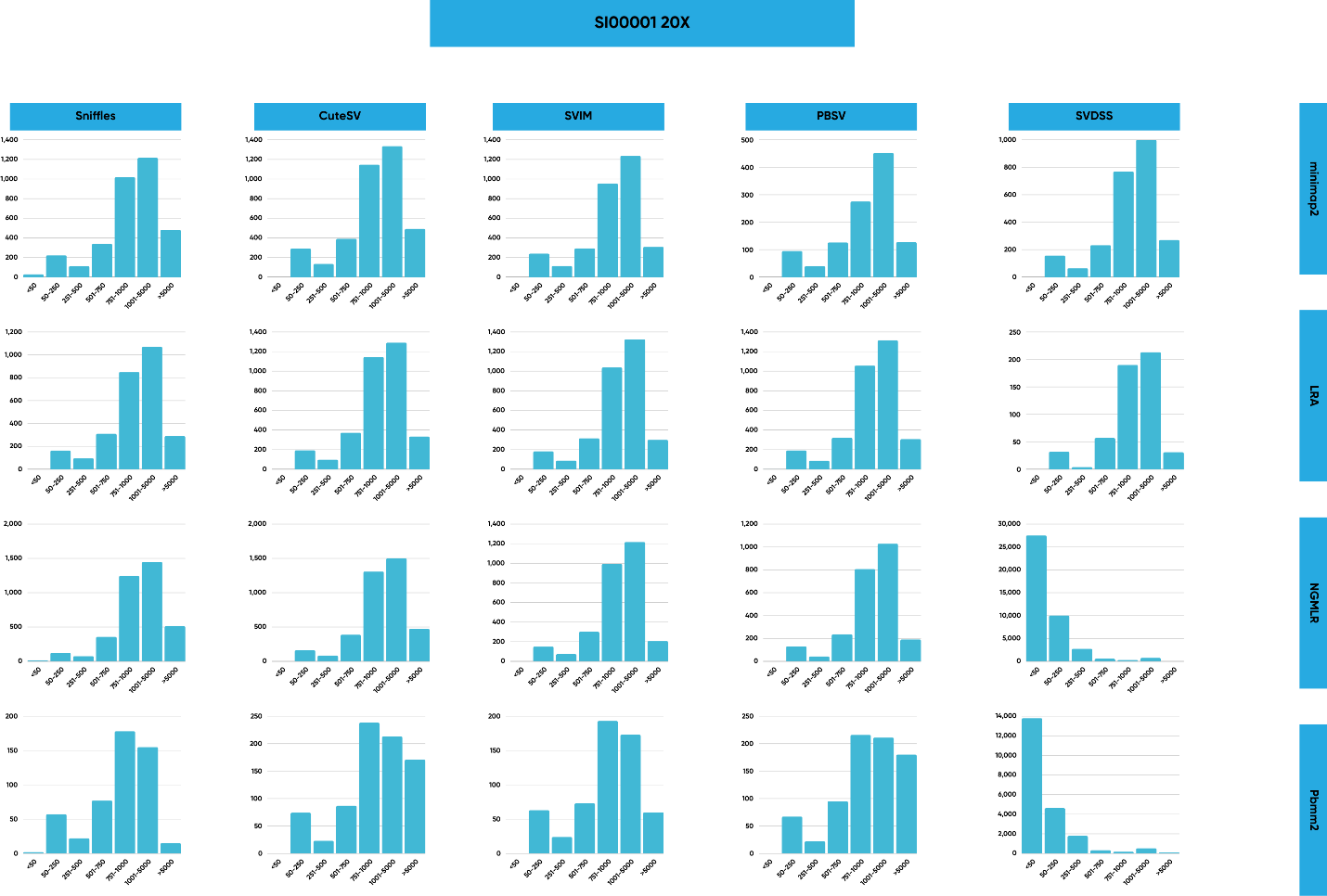


**Figure S10.** The chart bar represents the SV count for sample SI00001 at 20X coverage for all the SV callers (CuteSV, Sniffles, SVIM, PBSV and SVDSS) with the different aligners (Minimap2, LRA, Ngmlr and Pbmm2)


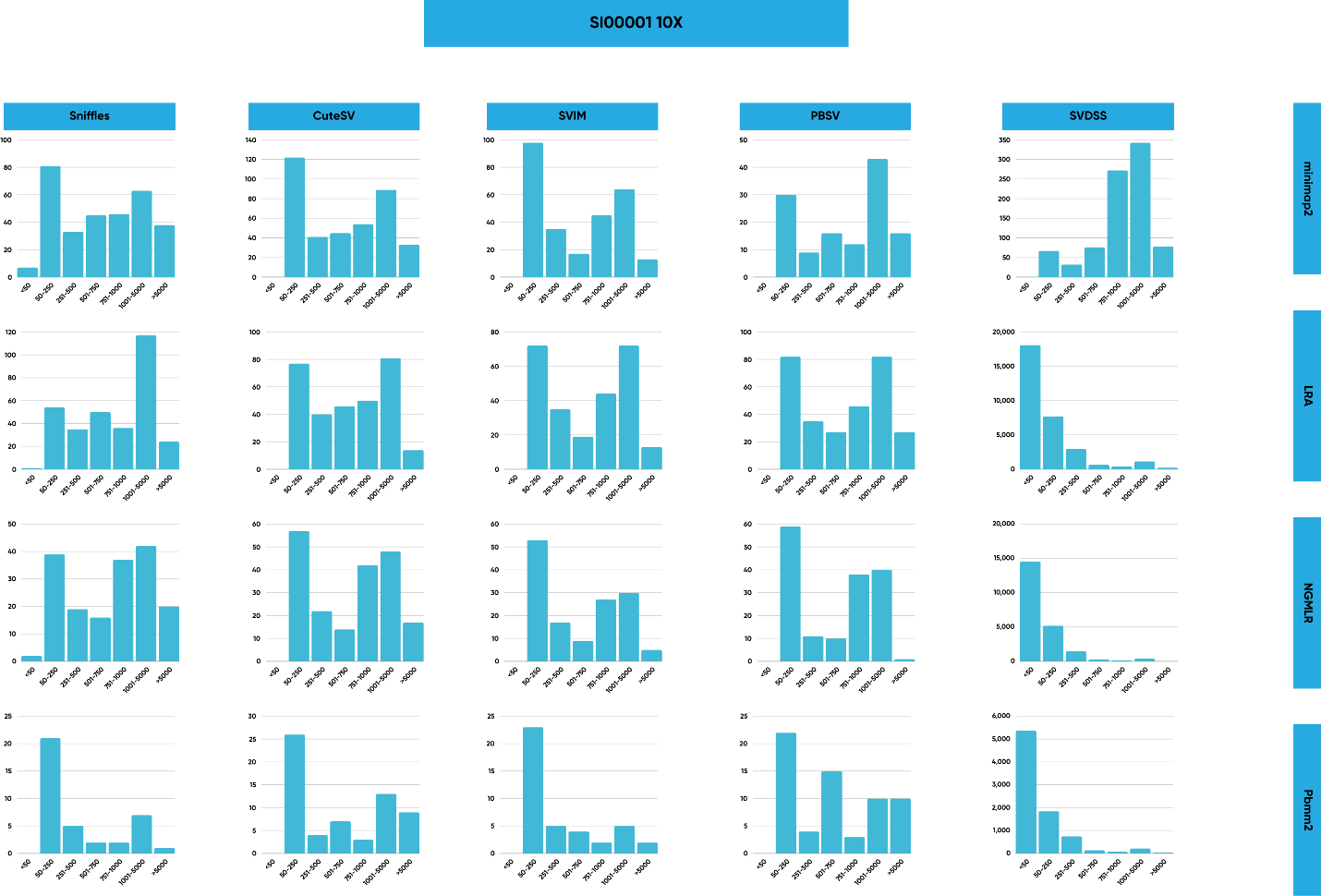


**Figure S11.** The chart bar represents the SV count for sample SI00001 at 10X coverage for all the SV callers (CuteSV, Sniffles, SVIM, PBSV and SVDSS) with the different aligners (Minimap2, LRA, Ngmlr and Pbmm2)
